# Muscle peripheral circadian clock drives nocturnal protein degradation via raised Ror/Rev-erb balance and prevents premature sarcopenia

**DOI:** 10.1073/pnas.2422446122

**Published:** 2025-05-05

**Authors:** Jeffrey J. Kelu, Simon M. Hughes

**Affiliations:** ^a^Randall Centre for Cell and Molecular Biophysics, School of Basic and Medical Biosciences, Faculty of Life Sciences and Medicine, King’s College London, London SE1 1UL, United Kingdom

**Keywords:** circadian clock, muscle, proteasome, autophagy, mTOR

## Abstract

How central and peripheral circadian clocks regulate protein metabolism and affect tissue mass homeostasis has been unclear. Circadian shifts in the balance between anabolism and catabolism control muscle growth rate in young zebrafish independent of behavioral cycles. Here, we show that the ubiquitin–proteasome system (UPS) and autophagy, which mediate muscle protein degradation, are each upregulated at night under the control of the muscle peripheral clock. Perturbation of the muscle transcriptional molecular clock disrupts nocturnal proteolysis, increases muscle growth measured over 12 h, and compromises muscle function. Mechanistically, the shifting circadian balance of Ror and Rev-erb regulates nocturnal UPS, autophagy, and muscle growth through altered TORC1 activity. Although environmental zeitgebers initially mitigate defects, lifelong muscle clock inhibition reduces muscle size and growth rate, accelerating aging-related loss of muscle mass and function. Circadian misalignment such as shift work, sleep deprivation, or dementia may thus unsettle muscle proteostasis, contributing to muscle wasting and sarcopenia.

## Significance

Circadian disruption, occurring in shift work, sleep deprivation, or dementia, increases the risk of sarcopenia, the age-related loss of muscle mass and strength. The cell-intrinsic and extrinsic effects of circadian disruptions, and the mechanistic basis of sarcopenia, remain unclear. We demonstrated that Ror and Rev-erb, two important components of the muscle peripheral clock, are crucial for regulating nocturnal protein clearance via the ubiquitin–proteasome system and autophagy. In animals lacking a functional muscle clock, Ror and Rev-erb imbalances lead to altered circadian muscle growth and reduced muscle function. Notably, muscle clock disruption alone causes premature sarcopenia. Our findings highlight the potential of targeting Ror and Rev-erb to mitigate muscle health issues caused by circadian disruption.

Weakening circadian rhythms in older people are associated with worse aging prognosis, suggesting that intrinsic circadian clocks promote longevity (1). In model organisms, disruption of the transcriptional circadian clock leads to premature aging (2, 3). Moreover, extrinsic factors like time-restricted feeding (TRF) can extend lifespan in wild type (WT) animals, but not in clock mutants (4). As clock disruption leads to behavioral changes, altered drive from the central brain clock, and defective peripheral tissue clocks, it has been difficult to determine whether aging changes arise from cell-intrinsic or extrinsic effects. A recent advance found that both brain and muscle clocks interact to prevent premature muscle aging by maintaining behavioral rhythms such as feeding patterns (5).

Like all peripheral tissues, skeletal muscle possesses an autonomous circadian clock important for its physiology, metabolism, and growth (6). Questions remain, however, about which daily variations are truly muscle fiber autonomous and truly circadian (as opposed to driven by zeitgebers like light or feeding rhythms). Studies utilizing muscle-specific clock mutant (*Bmal1* knockout) mice suggest the necessity of the muscle clock in the circadian regulation of lipid, glucose, and protein metabolism (7–9); whereas studies utilizing clock-disrupted mice with reinstated *Bmal1* expression in muscle indicate a partial sufficiency of the muscle clock in the maintenance of glucose metabolism and muscle homeostasis (5, 10). Nevertheless, the muscle-intrinsic circadian mechanisms remain unclear.

We recently showed that zebrafish display circadian variation in muscle growth rate, even in the absence of physical activity and feeding (11). A striking observation was that proteasome inhibition could enhance muscle growth specifically at night, but only if the circadian clock was unperturbed (11). In addition, genes that code for muscle-specific E3 ligases (12) are rhythmically expressed with their peak levels at night in zebrafish (11, 13) and humans (14), as in the fasting/inactive phase in mice (8, 15–17). These findings raise the hypothesis that circadian protein degradation through ubiquitin–proteasome system (UPS) controls the rate of muscle growth. Macroautophagy (hereafter autophagy) also degrades proteins in muscle and regulates muscle mass (18, 19), and undergoes circadian/diel variation (8, 16). The role of autophagy in circadian muscle growth and its dependence on the muscle intrinsic clock without external zeitgebers, such as light and nutrition, remains unclear.

To test the role of the muscle peripheral clock in regulating growth, we expressed dominant-negative Clock protein specifically in muscle fibers (mΔCLK), disrupting clock output. mΔCLK removed the circadian difference in muscle growth by enhancing growth during subjective night, indicating a cell autonomous role of the muscle clock in limiting nocturnal muscle growth. This effect was linked to reduced protein ubiquitination and autophagy via suppression of MuRF and Ulk1 activity, respectively, at night. Inhibiting MuRF, autophagy, or the muscle clock increased muscle size but reduced maximal contractions. Mechanistically, autophagy is activated at night by relief of TORC1 inhibition, with its circadian regulation requiring the balanced actions of Rev-erbα/β (hereafter Nr1d1/2) and Rora/c. mΔCLK disturbed this balance, but pharmacological inhibition of Nr1d1/2 returned growth to normal. Although mΔCLK fish grew normally under environmental zeitgebers, with age they failed to maintain muscle mass or thrive, suggesting that the muscle clock prevents sarcopenia, defined herein as aging-related loss of muscle mass and strength, by promoting nocturnal proteostasis through ubiquitination and autophagy.

## Results

### Inhibition of Muscle Clock Perturbs Muscle Clock-Controlled Genes and Alters Circadian Muscle Growth.

**Fig. 1.**
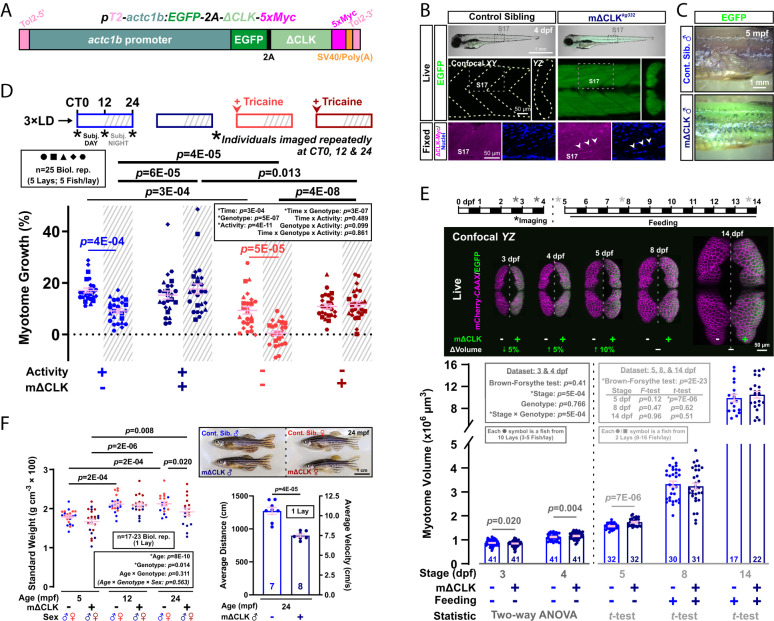
Inhibition of the muscle peripheral clock disrupts muscle clock output and alters circadian muscle growth. (*A*) Design of the muscle-specific dominant negative clock (mΔCLK) construct. (*B*) Brightfield/EGFP overlay (top row) showing the gross morphology of control and mΔCLK siblings at 4 dpf. Confocal *XY* (parasagittal) and *YZ* (transverse) slices of trunk musculature (boxes) showing uniform EGFP (live; middle row) and Myc-tagged-ΔCLK in myonuclei counterstained with Hoechst 33342 (fixed; bottom row). Arrowheads indicate Myc-positive myonuclei. (*C*) Brightfield/EGFP overlay showing persistence of EGFP in skinned myotome of mΔCLK but none in control sibling (Cont. Sib.) fish at 5 mpf. (*D*) Circadian muscle growth of myotome 17 from 3 to 4 dpf (% change) measured with *actc1b:mCherryCAAX* in control and mΔCLK siblings. Muscle growth of individual larvae (3xLD entrained) was tracked every 12 h (*) under free-run conditions (constant light) between 3 and 4 dpf as shown in schematics. Sibling control (light color symbols in all Figures) and mΔCLK transgenic (dark color symbols) were either unanesthetized (active; blue symbols) or anesthetized (inactive, arrowhead; red symbols). CT is circadian time. CT0-12 and CT12-24 are subjective (subj.) day and night, respectively. Statistics are three-way ANOVA (Time/Treatment/Genotype) with Bonferroni’s post hoc test. (*E*) Myotome volume measured at 3, 4, 5, 8, and 14 dpf in control and mΔCLK siblings that were raised under LD. Single confocal *YZ* (transverse) slices show muscle cross-sectional area visualized with *actc1b:mCherryCAAX* without (*Left*) or with mΔCLK (right; EGFP marker). Schematic above shows imaging and feeding timeline; break indicates separate biological replicates at 3 and 4 dpf from 5, 8, and 14 dpf. Statistics are two-way ANOVA (Stage/Genotype) with Bonferroni’s post hoc test for the 3 and 4 dpf dataset, and unpaired *t* test for the individual 5, 8, and 14 dpf datasets (unequal variance shown with Brown–Forsythe and *F*-tests precluded ANOVA). (*F*) Brightfield images (*Top*) showing the gross morphology of control and mΔCLK siblings at 24 mpf. Standard weight of male (blue) and female (red) control and mΔCLK siblings, measured at 5, 12, and 24 mpf. Statistics are two-way ANOVA (Age/Genotype) and three-way ANOVA (Age/Genotype/Sex) with Bonferroni’s post hoc test. Average swimming distance and velocity measured at 24 mpf; statistics are unpaired *t* test.

To test the cell autonomous role of the muscle peripheral clock, we created a transgenic line expressing EGFP and Myc-tagged dominant negative Clock protein (ΔCLK) under a muscle actin promoter, named *Tg(actc1b:EGFP-2A-ΔCLK-5xMyc)^kg333^* (hereafter mΔCLK; Fig. 1*A* and *SI Appendix*, Fig. S1 *A* and *B*). ΔCLK was exclusively expressed in muscle (Fig. 1 *B* and *C*), and abolished the rhythmicity of mRNAs encoding muscle clock-controlled genes (CCGs) in whole mΔCLK larvae entrained in 12 h:12 h light:dark (LD) regime until 3 d postfertilization (dpf) and then reared under “free-run” conditions (constant light; *SI Appendix*, Fig. S1 *C* and *D*). Thus, ΔCLK overexpression efficiently inhibits the muscle clock output.

To assess mΔCLK’s effect on muscle core clock components, RNA was collected from trunk/tail, which is mostly muscle tissue, between 3 and 4 dpf. The expression of clock genes was lower in the trunk than the whole body (*SI Appendix*, Fig. S2*A*), consistent with in situ hybridization results (20). In mΔCLK trunk, the truncated (*Δclk)* form of *clocka* mRNA was expressed evenly at all times of day and at ~50-fold higher level, contrasting with the circadian oscillation of endogenous *clocka* mRNA in nontransgenic control trunk (*SI Appendix*, Fig. S2*B*). mRNA oscillations of Bmal1:Clock target genes were reduced in mΔCLK trunk/tail, while minimal changes in whole larvae indicated intact circadian rhythms in the rest of the mΔCLK fish body (*SI Appendix*, Figs. S1*D* and S2*B*). These findings, confirmed in adult muscle (*SI Appendix*, Fig. S2*C*) and another transgenic line *kg332Tg* (*SI Appendix*, Fig. S3 *A*–*C*), indicate that mΔCLK disrupts muscle clock with minimal impact on non–muscle tissue clocks.

Next, we measured circadian changes in myotome volume, in the presence or absence of ΔCLK in the muscle, by repeatedly imaging individual larva every 12 h between 3 and 4 dpf under free-run (Fig. 1*D* and *SI Appendix*, Fig. S3*D*). At this age, larvae grow twice as fast in the day as at night (11). We showed that mΔCLK muscle grew faster than control at night by ~twofold (both in absolute terms and as % increase, *P* ≤ 6E-05) with no difference in day-time growth, abolishing the circadian growth difference (Fig. 1*D* and *SI Appendix*, Fig. S3*D*). This specific nocturnal growth enhancement was confirmed in the *kg332Tg* line (*SI Appendix*, Fig. S3*C*), but did not occur in a muscle EGFP control line (*SI Appendix*, Fig. S3 *E*–*G*). It was also independent of physical activity, as it persisted under anesthetic Tricaine (absolute and %, *P* ≤ 4E-08; Fig. 1*D* and *SI Appendix*, Fig. S3*D*). Physical activity nevertheless enhanced muscle growth, at least at night, both in control (absolute and %, *P* ≤ 4E-05) and mΔCLK fish (absolute and %, *P* ≤ 0.013; Fig. 1*D* and *SI Appendix*, Fig. S3*D*). Despite altered growth, gross muscle architecture appeared normal in mΔCLK larvae (*SI Appendix*, Fig. S3 *H–J*). Thus, the muscle peripheral clock normally limits muscle growth at night in zebrafish larvae.

### mΔCLK Disrupts Muscle Mass Homeostasis in Prefeeding Larvae and During Aging.

At 3 dpf, mΔCLK muscle was ~5% smaller than control (*P* = 0.020; Fig. 1*E*), a difference not observed in EGFP-only muscle (*SI Appendix*, Fig. S3*G*). By 4 dpf, mΔCLK (but not EGFP-only) muscle became ~10% larger than control muscle (*P* = 0.004; Fig. 1*E* and *SI Appendix*, Fig. S3*G*), reflecting the nocturnal growth enhancement (Fig. 1*D* and *SI Appendix*, Fig. S3*D*). When two distinct cohorts of fish were each measured at 5, 8, and 14 dpf, an overall increase in muscle size was observed with age (Fig. 1*E*). mΔCLK muscle remained larger than control at 5 dpf (by ~10%, *P* = 7E-06), but such difference disappeared in feeding larvae/juveniles at 8 and 14 dpf (Fig. 1*E*). We conclude that the muscle clock is required for muscle mass homeostasis in prefeeding larvae, but that growth normalizes thereafter, perhaps due to zeitgebers like light, food, or activity.

In fish, growth is continuous and up to 70% of adult body weight is muscle (21). As expected, in sibling control fish, both body weight and length increased successively between 5, 12, and 24 months postfertilization (mpf; *SI Appendix*, Fig. S4). In contrast, mΔCLK fish grew normally until 12 mpf but showed no further growth by 24 mpf (*SI Appendix*, Fig. S4). Whereas body sizes were indistinguishable between mΔCLK and siblings prior to 12 mpf, by 24 mpf, the same mΔCLK fish were lighter, shorter, and skinnier, with reduced standard weight and body mass index (BMI; *P* ≤ 0.02), indicating compromised muscle growth (Fig. 1*F* and *SI Appendix*, Fig. S4). This growth retardation was age- but not sex-dependent. mΔCLK fish were also less active than siblings at 24 mpf (*P* = 4E-05; Fig. 1*F*), which could arise either directly from defective muscle or through an effect of the muscle clock on other tissues such as the nervous system. Thus, the muscle intrinsic clock is required for maintaining muscle mass and function during aging.

### MuRF-Mediated Ubiquitination Is Higher at Night, Contributes to Nocturnal Muscle Protein Degradation and Limits Muscle Growth.

**Fig. 2.**
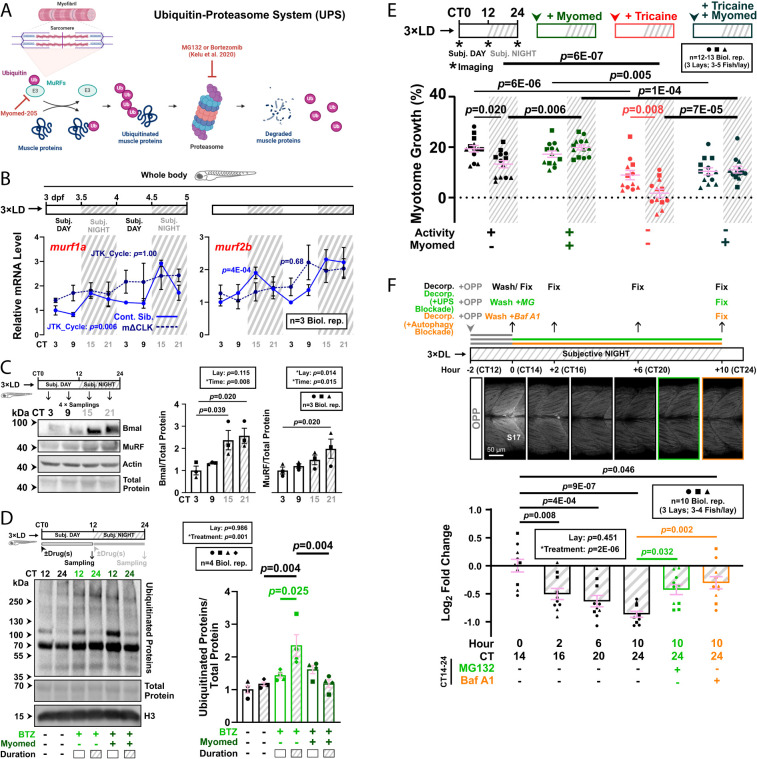
MuRF accumulation at night limits nocturnal muscle growth. (*A*) The UPS pathway highlighting relevant proteins and inhibitors (red). (*B*) Circadian mRNA profiles of muscle-specific E3 ligase transcripts *murf1a/trim63a* and *murf2b/trim55b* in control (blue, solid line) and mΔCLK siblings (dark blue, dotted line). RNA collected from 3xLD entrained fish under free-run was analyzed by RT-qPCR (*SI Appendix*, Fig. S1*C*). Statistics from JTK_Cycle. (*C*) Circadian accumulation of MuRF and Bmal proteins under free-run between 3 and 4 dpf analyzed by western blotting. Note that MuRF antibody may detect the products of more than one zebrafish MuRF gene and that protein quantifications are relative to total protein in the sample. Statistics are two-way ANOVA (Lay/Time) with Bonferroni’s post hoc test. (*D*) Circadian variations in proteasomal degradation of ubiquitinated proteins. Larvae under free-run between 3 and 4 dpf were treated with either DMSO, bortezomib (BTZ) alone, or BTZ and Myomed during either CT0-12 or CT12-24, and protein analyzed by western blotting. Statistics are two-way ANOVA (Lay/Treatment) with Bonferroni’s post hoc test. (*E*) Circadian muscle growth after MuRF inhibition in larvae under free-run between 3 and 4 dpf that were either active or inactive and treated with DMSO or Myomed. Statistics are two-way ANOVA (Time/Treatment) with Bonferroni’s post hoc test. (*F*) Nocturnal protein degradation in muscle measured by OPP decorporation. Newly synthesized proteins in DL-entrained larvae at 3 dpf were labeled with OPP between CT12 and 14 and allowed to degrade overnight under free-run. Fish fixed at 0, 2, 6, and 10 h post OPP-washout were imaged in single confocal *XY* parasagittal slices and OPP signal in S17 visualized with fluorescent Click-iT chemistry and quantified after background subtraction (*SI Appendix*, Fig. S5 *D* and *E*). MG132 (green symbols) or bafilomycin A1 (orange symbols) treatment after OPP-washout reduced decorporation. Log_2_ fold change in fluorescence signal relative to control samples at 0 h. Statistics are two-way ANOVA (Lay/Treatment) with Bonferroni’s post hoc test.

To study how the muscle clock regulates muscle growth, we focused on the larval model because of its amenability for true circadian analysis and our evidence implicating the UPS protein degradation pathway (11) (Fig. 2*A*). We observed mRNA and protein oscillations of muscle-specific E3 ligase MuRF in entrained WT larvae under free-run (Fig. 2 *B* and *C*), with peak expression observed in the subjective night, coinciding with the slower rate of muscle growth (Fig. 1*D*). MuRF oscillations, at least for protein, were genuinely circadian as they were entrainable to a reversed LD cycle (DL; *SI Appendix*, Fig. S5*A*). To examine E3 ligase activity, we analyzed ubiquitinated proteins (Fig. 2*D*). Although their level did not differ between late subjective day and night, when their degradation was blocked using bortezomib (Fig. 2*A*), ubiquitinated proteins accumulated by over twofold during the subjective night (CT12-24; *P* = 0.004), but not during the subjective day (CT0-12; Fig. 2*D*). We conclude that the enhanced nocturnal E3 ligase levels correlate with a higher rate of on-going ubiquitination.

To determine whether MuRFs drive ubiquitination within muscle, larvae were cotreated with a MuRF inhibitor, Myomed-205 (hereafter Myomed; Fig. 2*A* and *SI Appendix*, Fig. S5*B*). Nocturnal ubiquitinated protein abundance was reduced by Myomed (*P* = 0.004), whereas daytime abundance appeared unaffected (Fig. 2*D*). Thus, MuRFs drive more abundant protein ubiquitination at night. As MuRFs are only expressed in skeletal and cardiac muscle (22, 23) and the heart is small, the observed twofold whole body increase in ubiquitinated proteins after MuRF inhibition is likely primarily in skeletal muscle. Functional cardiac assays revealed no effect of Myomed treatment on heart rate or interbeat interval (*SI Appendix*, Fig. S5*B*).

Muscle size increased slightly after MuRF inhibition (*P* = 0.029; *SI Appendix*, Fig. S5*C*), consistent with rodent MuRF-knockout models (24, 25). Circadian muscle growth analysis showed that Myomed enhanced growth by ~twofold in subjective night (*P* = 0.006), independent of activity (*P* = 7E-05), but had no effect during the subjective day (Fig. 2*E*). Slowing of heart rate with either tricaine or the K^+^-channel blocker terfenadine neither mimics nor prevents the effects of Myomed on skeletal muscle (Fig. 2*E* and *SI Appendix*, Fig. S5*B*). These data show that MuRFs act in a circadian manner upstream of the proteasome to induce nocturnal muscle protein degradation, and thereby restricting muscle growth at night.

### UPS Contributes to Nascent Protein Turnover in Muscle.

Assessing protein turnover through ubiquitinated protein accumulation requires inhibitors and does not measure absolute rates. To overcome this, we analyzed nascent muscle protein turnover in vivo and studied their route of degradation (Fig. 2*F* and *SI Appendix*, Fig. S5 *D* and *E*). Newly synthesized peptides in muscle exhibited exponential decay (*R^2^* = 0.60) with a half-life of ~13 h (*SI Appendix*, Fig. S5 *D* and *E*). Thus, only around 50% of the nascent peptides were lost overnight (Fig. 2*F*), suggesting that degradation rates are saturable and insufficient to match synthesis, consistent with net muscle growth even at night in these young larvae. The overnight loss of OPP signal was attenuated by UPS inhibitor MG132 (*P* = 0.032; Fig. 2 *A* and *F*), indicating that UPS contributes to the degradation of nascent muscle proteins in vivo.

### Muscle ΔCLK Expression Compromises Proteasomal Degradation by Attenuating Murf-Mediated Ubiquitination.

**Fig. 3.**
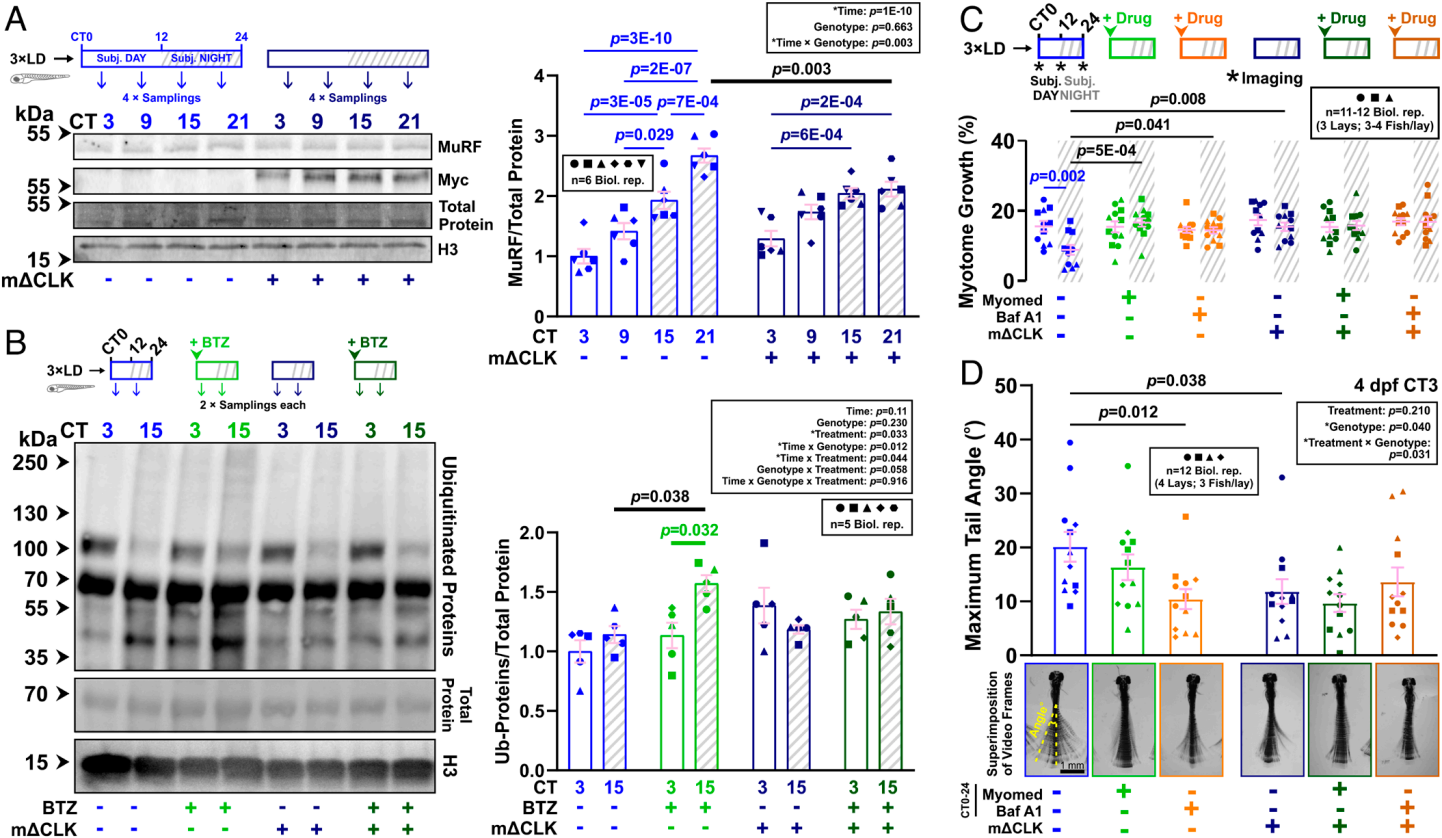
Inhibition of the muscle clock disrupts circadian variations in proteasomal flux in the muscle. (*A*) Effect of muscle clock inhibition on circadian accumulation of MuRF in control and mΔCLK sibling larvae under free-run between 3 and 4 dpf. MuRF and Myc-tagged ΔCLK abundance were normalized to total protein. Statistics are two-way ANOVA (Time/Genotype) with Bonferroni’s post hoc test. (*B*) Effect of muscle clock inhibition on circadian variations in proteasomal degradation of ubiquitinated proteins in control and mΔCLK sibling larvae under free-run between 3 and 4 dpf treated from CT0 with either DMSO or bortezomib (BTZ), and protein analyzed by western blotting at CT3 and CT15. Statistics are three-way ANOVA (Time/Genotype/Treatment) with Bonferroni’s post hoc test. (*C* and *D*) Circadian myotome growth between 3 and 4 dpf (*C*) or tail displacement at 4 dpf (*D*) measured in control and mΔCLK sibling larvae under free-run that were treated from CT0-24 with either DMSO, Myomed, or bafilomycin A1 (Baf A1). Images (*D*) are superimposition of video frames. Statistics are two-way ANOVA (Time/Treatment) with Bonferroni’s post hoc test.

Muscle clock inhibition abolished MuRF transcript oscillations (Fig. 2*B*). Nevertheless, MuRF proteins still accumulated in the subjective night in mΔCLK fish, though their peak abundance at CT21 was reduced by ~20% (*P* = 0.003; Fig. 3*A*). This reduction was paralleled by a lower nocturnal rate of on-going protein ubiquitination (Fig. 3*B*) and a loss/drop in circadian variation in ubiquitinated protein abundance in mΔCLK fish (*SI Appendix*, Fig. S6*A*). Proteasome activity, which peaked in late subjective night in control fish (*P* = 0.030), remained largely unaltered in whole mΔCLK fish (*SI Appendix*, Fig. S6*B*). These data suggest that the muscle clock regulates proteasomal degradation through ubiquitination rather than proteasome activity.

Additionally, Myomed and mΔCLK showed a nonadditive effect on promoting muscle growth at night (Fig. 3*C*), strongly suggesting that muscle clock and MuRFs act on the same pathway to enhance nocturnal protein degradation and restrict growth. Although both mΔCLK and Myomed treatment increased muscle growth (Fig. 1*E* and *SI Appendix*, Fig. S5*C*), neither increased muscle function (Fig. 3*D*), indicating compromised specific force and maladaptation. These findings suggest that muscle clock-driven UPS-mediated protein turnover is essential for muscle health.

### Autophagic Degradation Limits Muscle Growth at Night.

**Fig. 4.**
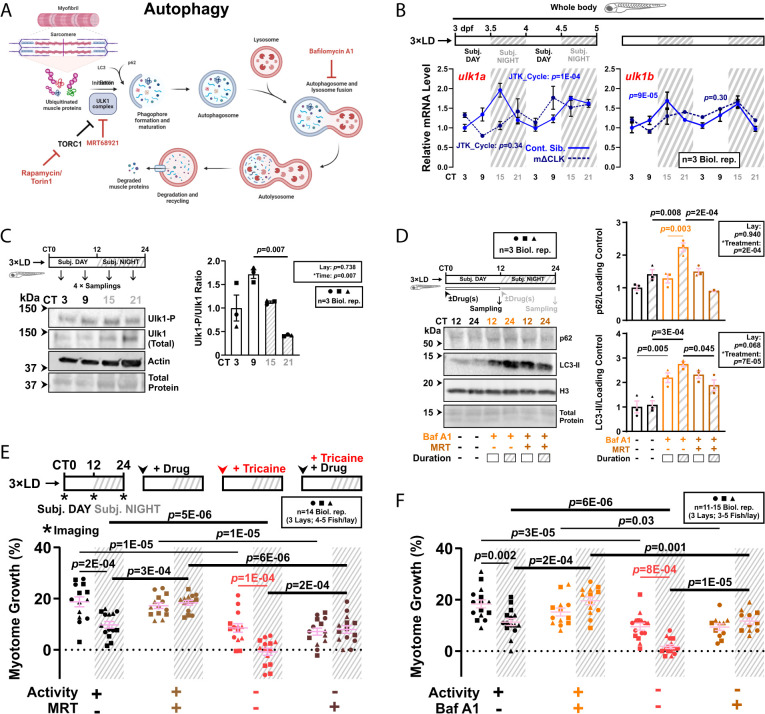
Ulk1-dependent autophagy activation at night limits nocturnal muscle growth. (*A*) The autophagy pathway highlighting relevant proteins and inhibitors (red). (*B*) Circadian mRNA levels of *ulk1a* and *ulk1b* in 3xLD entrained whole larvae of control and mΔCLK siblings under free-run analyzed by RT-qPCR (*SI Appendix*, Fig. S1*C*). Statistics from JTK_Cycle. (*C*) Circadian oscillation in Ulk1^Ser757^ phosphorylation in larvae under free-run between 3 and 4 dpf assessed by western blotting. Statistics are two-way ANOVA (Lay/Time) with Bonferroni’s post hoc test. (*D*) Circadian variations in autophagic degradation of p62 and LC3-II in larvae under free-run treated with DMSO, bafilomycin A1 (Baf A1) alone, or both Baf A1 and MRT68921 (MRT) during either CT0-12 or CT12-24 and analyzed by western blotting. Statistics are two-way ANOVA (Lay/Treatment) with Bonferroni’s post hoc test. (*E* and *F*) Circadian myotome growth after autophagy inhibition in entrained active or inactive larvae treated under free-run between 3 and 4 dpf with DMSO, MRT68921 (*E*), or bafilomycin A1 (*F*). Statistics are two-way ANOVA (Time/Treatment) with Bonferroni’s post hoc test.

As autophagy also disposes of ubiquitinated proteins, we next assessed its role in regulating circadian muscle growth. Autophagy is canonically initiated through the activation of Ulk1, which is inhibited when phosphorylated at Ser757 by the TORC1 complex (Fig. 4*A*). The transcripts of *ulk1a* and *ulk1b*, along with other autophagy-related genes (*SI Appendix*, Fig. S7*A*), displayed circadian oscillation (JTK_Cycle: *P* < 0.01) in entrained whole larvae (Fig. 4*B*) and trunk/tail under free-run (*SI Appendix*, Fig. S7*B*), peaking during subjective night. At the protein level, Ulk1 also oscillated and accumulated during subjective night (Fig. 4*C*, *SI Appendix*, Fig. S7*C*), with a phase delay of ~6 h compared to its mRNA rhythm (compare Fig. 4*B* and *SI Appendix*, Fig. S7*C*). The Ulk1^Ser757^-P/Ulk1 ratio was the highest in the late subjective day and lowest in the late subjective night (Fig. 4*C*). This reversed under DL (*SI Appendix*, Fig. S7*D*). We conclude that autophagy initiation by Ser757-dephosphorylated Ulk1 is clock-driven, high nocturnally, and likely suppressed by TORC1 in the day.

To study circadian autophagic degradation, we treated larvae with bafilomycin A1, which prevents the fusion of lysosomes to autophagosomes (Fig. 4*A* and *SI Appendix*, Fig. S5*B*), at either CT0-12 or CT12-24 (Fig. 4*D*). Blockade of autophagic degradation caused accumulation of p62 in subjective night (*P* = 0.008) and LC3-II in both subjective day (*P* = 0.005) and night *P* = 3E-04), indicating on-going autophagy but higher nocturnal flux (Fig. 4*D*). When larvae were treated with MRT68921, a small molecule inhibitor of Ulk1/2 (Fig. 4*A* and *SI Appendix*, Fig. S5*B*), the accumulation of p62 (*P* = 2E-04) and LC3-II (*P* = 0.045) were specifically reduced in subjective night (Fig. 4*D*). This suggested that Ulk1/2-mediated autophagic flux is more prominent at night. Congruently, MRT68921 enhanced circadian muscle growth during subjective night (*P* = 3E-04), independent of physical activity (*P* = 2E-04), but not during subjective day (Fig. 4*E*). Similar effects on growth were caused by the downstream autophagy inhibitor bafilomycin A1 (Figs. 3*C* and 4*F*). Bafilomycin A1 also attenuated the degradation of newly synthesized peptides in the muscle (*P* = 0.002; Fig. 2*F*) as well as reducing maximal tail displacement (*P* = 0.012; Fig. 3*D*). We conclude that autophagy contributes to muscle protein degradation and limits larval muscle growth at night, and its inhibition compromises muscle function.

Notably, no additive effect was observed when UPS and autophagy pathways were blocked simultaneously (compare *SI Appendix*, Fig. S7*E* with Figs. 2*E*, 3*C* and 4 *E* and *F*, and Fig. 4 *B* and *C* in ref. 7), and there was no evidence for cross inhibition of UPS by autophagy inhibitors (*SI Appendix*, Fig. S8 *A* and *B*) or vice versa (*SI Appendix*, Fig. S8 *B* and *C*). We, therefore, propose a model in which muscle proteins are normally degraded at night by both the UPS and autophagy pathways, and that suppression of either catabolic pathway removes a limit on nocturnal muscle growth.

### Muscle Clock Inhibition Activates TORC1 and Suppresses Autophagy at Night.

**Fig. 5.**
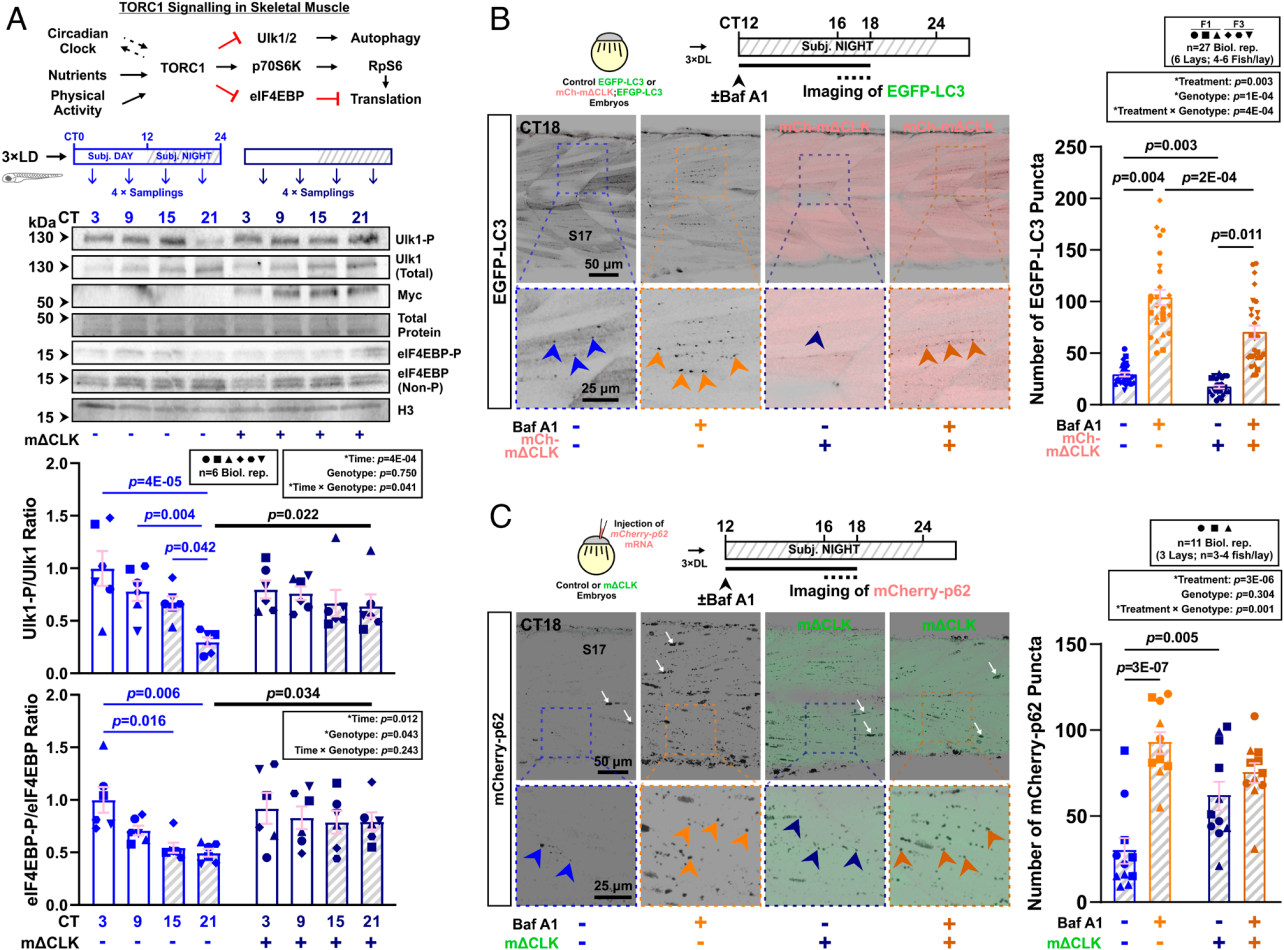
Inhibition of the muscle clock reduces autophagosome formation and autophagic activity in muscle at night. (*A*) The TORC1 signaling pathway in skeletal muscle highlighting relevant downstream effectors. Effect of muscle clock inhibition on circadian oscillation of Ulk1^Ser757^ and eIF4EBP^Thr37/46^ phosphorylation in entrained larvae under free-run between 3 and 4 dpf in control and mΔCLK siblings. Statistics are two-way ANOVA (Time/Genotype) with Bonferroni’s post hoc test. (*B*) Autophagic flux in muscle measured in *Tg(CMV:EGFP-map1lc3b)*^zf155^ control and sibling *Tg(CMV:EGFP-map1lc3b)^zf155^;mCherry-mΔCLK^kg334Tg^* DL-entrained larvae under free-run between 3 and 4 dpf treated with DMSO or bafilomycin A1 (Baf A1) at CT12 for 4 to 6 h. Confocal parasagittal images at CT16-18 visualized the number of EGFP-LC3 puncta (arrowheads) in *S17*. Statistics are two-way ANOVA (Treatment/Genotype) with Bonferroni’s post hoc test. F1/F3, filial generation. (*C*) Autophagic flux in muscle measured with *mCherry-p62* mRNA injection at one-cell stage into sibling control and *EGFP-mΔCLK^kg333Tg^* transgenic embryos, which were entrained and under free-run treated with DMSO or bafilomycin A1 (Baf A1) at CT12 for 4 to 6 h. Confocal parasagittal images at CT16-18 visualized the number of mCherry-p62 puncta (arrowheads) in sarcoplasm in *S17*. Some signals also associated with nuclei (white arrows), as previously reported (28, 29). Statistics are two-way ANOVA (Treatment/Genotype) with Bonferroni’s post hoc test.

After muscle clock inhibition, the oscillations of *ulk1a* and *ulk1b* were perturbed (Fig. 4*B* and *SI Appendix*, Fig. S7*B*). Nonetheless, Ulk1 protein remained cyclic in mΔCLK fish (Fig. 5*A* and *SI Appendix*, Fig. S9*A*). The discrepancy between mRNA and protein rhythms, as seen in other studies (26, 27), prompted further investigation of posttranslational regulation. Phosphorylation of Ulk1 at Ser757 was increased threefold (*P* = 0.022) during late subjective night in mΔCLK fish, abolishing the circadian changes in Ulk1^Ser757^-P/Ulk1 ratio (Fig. 5*A*). Consistent with the circadian changes in Ulk1^Ser757^-P, phosphorylation of eIF4EBP^Thr37/46^, another direct TORC1 target (Fig. 5*A* and *SI Appendix*, Fig. S10), was low at night in controls (*P* = 0.006) but elevated during late subjective night in mΔCLK fish (*P* = 0.034, Fig. 5*A*), abolishing its oscillation. These findings suggest TORC1 is activated at night after muscle clock inhibition to suppress autophagy. Indeed, although bafilomycin A1 still induced accumulation of LC3-II during subjective night in mΔCLK larvae (*P* = 0.035), the induction appeared less strong when compared to control siblings (*SI Appendix*, Fig. S9*B*), suggesting reduced autophagic flux. To assess autophagic flux in muscle, we used an EGFP-LC3 fusion protein to measure autophagosome formation microscopically at CT16-18. We observed ~50% fewer EGFP-LC3 puncta (*P* = 0.003) in mΔCLK muscle (using a new mΔCLK*^kg334^* line with an mCherry reporter; hereafter mCh-mΔCLK; *SI Appendix*, Fig. S9 *C* and *D*) compared to control muscle (Fig. 5*B* and *SI Appendix*, Fig. S9*E*), suggesting a reduction in autophagosome formation. When autophagic degradation was blocked with bafilomycin A1, EGFP-LC3 puncta still accumulated in mCh-mΔCLK muscle (*P* = 0.011), but the accumulation was significantly attenuated (*P* = 2E-04, Fig. 5*B* and *SI Appendix*, Fig. S9*E*), indicating reduced flux. We conclude that muscle clock inhibition hinders autophagy induction during the subjective night.

Consistent with an autophagy-deficient phenotype in mΔCLK muscle, we found that autophagic substrate mCherry-p62 failed to be cleared, and thus accumulated (*P* = 0.005; Fig. 5*C* and *SI Appendix*, Fig. S9*F*). Similar accumulation of p62 was previously observed in autophagy-deficient muscle fibers in mice (30, 31). Given that autophagic flux fails to rise at night in mΔCLK fish, we hypothesized that pharmacological inhibition of autophagy would not alter muscle growth or function in mΔCLK. Indeed, muscle growth and maximal tail displacement in mΔCLK larvae were insensitive to bafilomycin A1 (Fig. 3 *C* and *D*). The nonadditivity caused by clock blockade and autophagy inhibition suggests that these processes act on the same pathway to limit nocturnal muscle growth and function.

### Rorc/Nr1d1 Clock Activator/Repressor Ratio Controls Nocturnal Muscle Growth.

**Fig. 6.**
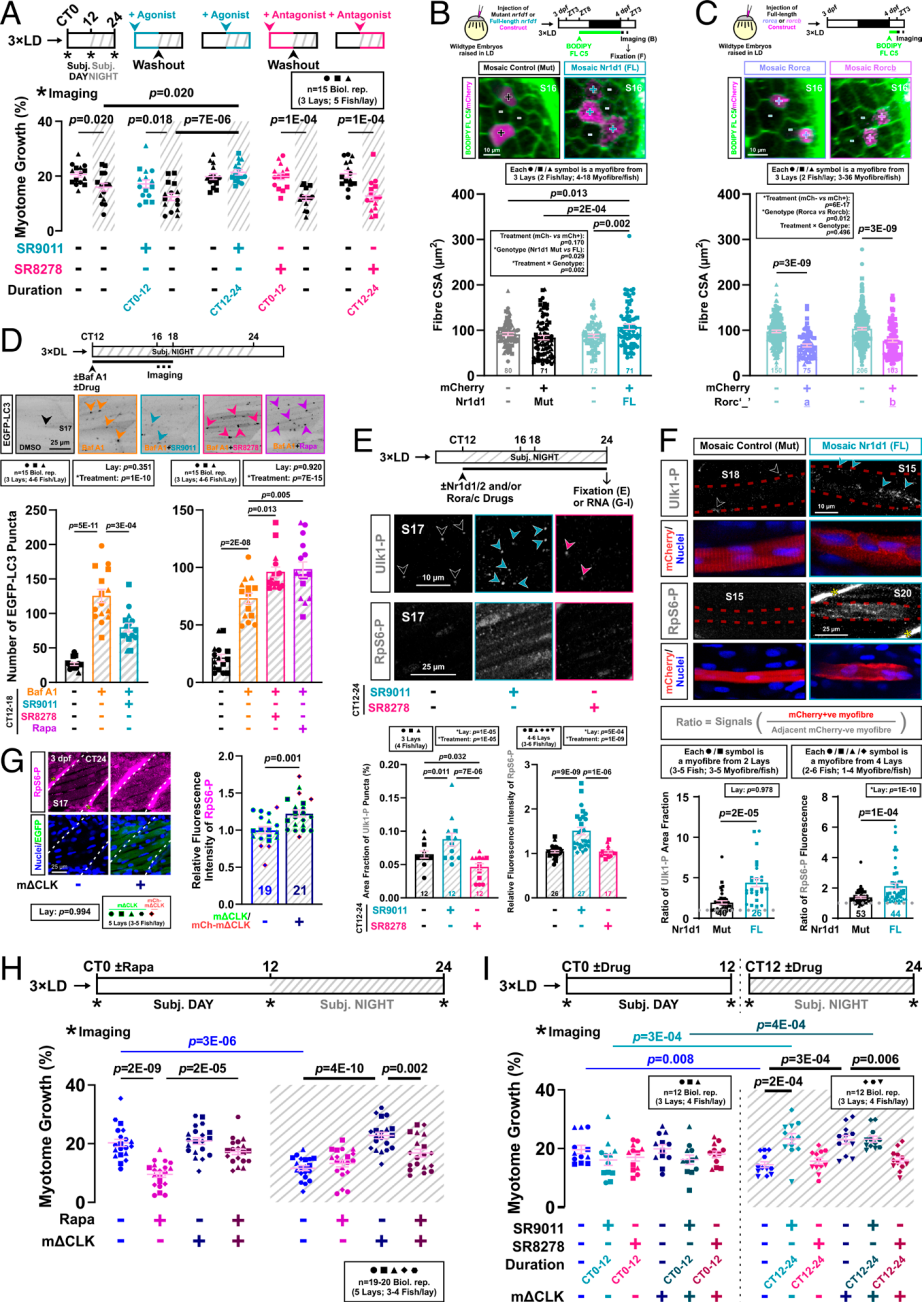
Nr1d1 promotes muscle growth by suppressing autophagy via TORC1 activation. (*A*) Circadian muscle growth after Nr1d1/2 activation (SR9011) or inhibition (SR8278) during subjective day (CT0-12) or night (CT12-24) measured in entrained *actc1b:mCherryCAAX* larvae under free-run treated with drug or DMSO control. Statistics are two-way ANOVA (Time/Treatment) with Bonferroni’s post hoc test. (*B* and *C*) Effect of Nr1d1 (*B*) or Rorca/b (*C*) mosaic overexpression on myofiber growth in embryos injected at one-cell stage with DNA encoding either mutant (Mut) or full-length (FL) mCherry-2A-Nr1d1 (*B*; see also *SI Appendix*, Fig. S11*B*), or full-length mCherry-2A-Rorca/b (see *SI Appendix*, Fig. S11*E*). Entrained larvae were labeled with BODIPY-Ceramide and single confocal transverse slices at 4 dpf at ZT3 permitted quantification of cross-sectional area of transgenic (+) and nontransgenic (−) myofibers in *S15*−*S18*. Statistics are two-way ANOVA (Treatment/Genotype) with Bonferroni’s post hoc test. ZT, zeitgeber time. (*D*) Effect of Nr1d1/2 agonism or antagonism on nocturnal autophagic flux measured with Tg(*CMV:EGFP-map1lc3b)* in DL-entrained larvae under free-run between 3 and 4 dpf treated from CT12 with either DMSO, bafilomycin A1 (Baf A1), or Baf A1 and SR9011, SR8278, or rapamycin. Single confocal parasagittal slices between CT16 and 18 permitted quantification of EGFP-LC3 puncta (arrowheads). Statistics are two-way ANOVA (Lay/Treatment) with Bonferroni’s post hoc test. (*E*) Effects of Nr1d1/2 agonism or antagonism on TORC1-mediated phosphorylation of Ulk1^Ser757^ in muscle. Entrained larvae under free-run between 3 and 4 dpf were treated from CT12 with either DMSO, SR9011, or SR8278 and then fixed at CT24. After immunodetection, phosphorylated Ulk1^Ser757^ puncta (arrowheads) and RpS6^Ser240/244^ intensity were quantified in single confocal parasagittal slices in *S17*. Statistics are two-way ANOVA (Lay/Treatment) with Bonferroni’s post hoc test. (*F*) Effects of Nr1d1 mosaic overexpression on TORC1-mediated phosphorylation of Ulk1^Ser757^ and RpS6^Ser240/244^ in myofibers in entrained 4 dpf larvae from embryos injected at the one-cell stage with either Mut or FL mCherry-2A-Nr1d1. After immunodetection and nuclear counterstain, phosphorylated Ulk1^Ser757^ puncta (arrowheads) and RpS6^Ser240/244^ intensity were quantified in single confocal parasagittal slices in mCherry-expressing myofibers (red dashes) and their neighbors in *S10*−*S22*. Asterisks denote unspecific labeling of RpS6-P^Ser240/244^ at myosepta (*SI Appendix*, Fig. S10*B*). The ratio of fluorescent signals between pairs of neighboring mCherry-positive and -negative myofibers was calculated as indicated in inset. Statistics are two-way ANOVA (Lay/Treatment) with Bonferroni’s post hoc test. (*G*) Effect of muscle clock inhibition on TORC1 activity in the muscle in sibling control and mΔCLK entrained larvae under free-run fixed at CT24. After immunodetection and nuclear counterstain, phosphorylated RpS6^Ser240/244^ intensity was quantified in single confocal parasagittal slices in *S17* (white dashes). Asterisks denote unspecific labeling of RpS6^Ser240/244^-P at myosepta (*SI Appendix*, Fig. S10*B*). EGFP reflects ΔCLK expression. Statistics are two-way ANOVA (Lay/Genotype) with Bonferroni’s post hoc test (symbol colors indicate lays from EGFP-mΔCLK and mCherry-mΔCLK outcrosses). (*H*) Effect of TORC1 inhibition on circadian muscle growth measured in sibling control and mΔCLK entrained larvae under free-run between 3 and 4 dpf treated from CT0-24 with either DMSO or rapamycin. Statistics are three-way ANOVA (Time/Genotype/Treatment) with Bonferroni’s post hoc test. (*I*) Circadian muscle growth measured in sibling control and mΔCLK entrained larvae under free-run between 3 and 4 dpf treated with either DMSO, SR9011, or SR8278 during either CT0-12 or CT12-24. Statistics are two-way ANOVA (Treatment/Genotype) with Bonferroni’s post hoc test. Due to the size of the experiment, different lays were used at CT0-12 and CT12-24.

We next asked how the muscle clock limits muscle growth at night. In mice, several direct Bmal1:Clock targets within the transcription–translation feedback loop (*SI Appendix*, Fig. S1*A*) are essential for normal myogenesis and muscle regeneration (*SI Appendix*, Table S1). Our focus turned to the stabilization loop, as *rorca/b* show substantial gene expression changes in mΔCLK muscle (*SI Appendix*, Fig. S2*B*). Notably, *Rev-erbα/Nr1d1* has been reported to increase muscle mass by suppressing autophagy (*SI Appendix*, Table S1). To explore the role of Nr1d1 in the regulation of muscle growth in zebrafish, we first utilized two small molecules, SR9011 and SR8278 which, though potent Nr1d1/2 agonist and antagonist, respectively, do not perturb gross morphology or health (*SI Appendix*, Fig. S11*A*). Agonist SR9011 specifically increased muscle growth during the subjective night (*P* = 0.020), but had no effect during subjective day (Fig. 6*A*). The effect was indistinguishable from that occurring in mΔCLK fish or by blockade of autophagy (compare Figs. 6*A* with 1*D*, 3*C*, and 4 *E* and *F*). In contrast, the antagonist SR8278 had no effect at either time of day (Fig. 6*A*). To rule out potential off-target effects of the Nr1d1 drugs (32), we recapitulated the effects of Nr1d1 agonism by injecting plasmid DNA expressing mCherry and Nr1d1 from the *actc1b* promoter (*SI Appendix*, Fig. S11 *B* and *C*). Fibers overexpressing full-length Nr1d1 became larger than their nonexpressing neighbors (*P* = 0.002), whereas fibers expressing a control Nr1d1 deletion mutant fragment did not (Fig. 6*B*). Nr1d1-expressing myofibers were significantly larger than controls (*P* = 2E-04; Fig. 6*B*). Taken together, these data show that Nr1d1 drives fiber hypertrophy at night.

Rorc acts as a competitive transcriptional activator opposing the repressing function of Nr1d1/2 (33). Consistent with such a competitive regulatory model, mosaic overexpression of Rorca/b in myofibers reduced their size (Fig. 6*C* and *SI Appendix*, Fig. S11 *E* and *F*). Thus, Rorc/Nr1d1 balance appears to regulate muscle fiber growth rate.

### Rorc/Nr1d1 Ratio Activates TORC1 Signaling and Suppresses Autophagy in Muscle.

After Nr1d1 activation, no clear promyogenic signature was observed at the mRNA level (*SI Appendix*, Fig. S11*G*). Instead, fiber hypertrophy resulting from Nr1d1 activation/overexpression could occur through the suppression of autophagy, as previously implicated (*SI Appendix*, Table S1). Congruently, SR9011 decreased (*P* = 3E-04) and SR8278 increased (*P* = 0.013) nocturnal autophagy flux (Fig. 6*D*). These effects on autophagy were likely mediated via TORC1, as muscle Ulk1 phosphorylation at Ser757 was elevated and suppressed by the agonist and antagonist, respectively (Fig. 6*E*). Additionally, a modest increase in phosphorylation of the indirect TORC1-target, ribosomal protein S6 (RpS6) at Ser240/244 (*SI Appendix*, Fig. S10), was detected in agonist-treated muscle fibers (Fig. 6*E*). Similar increase in Ulk1^Ser757^-P and RpS6^Ser240/244^-P was observed in the mosaic fibers overexpressing Nr1d1 when compared to fibers overexpressing the nonfunctional mutant control (Fig. 6*F*). Together, these data indicate that Nr1d1 represses autophagy by stimulating TORC1 in muscle.

We next asked how the transcriptional repressor Nr1d1 activates TORC1. Several proteins, including Gator1 and the Tsc family, are known inhibitors of TORC1 (34). The Tsc1 gene has been reported to be downregulated in C2C12 myotubes transfected with Nr1d1 (35). Zebrafish Tsc genes contain putative Nr1d1/2 and Rora/c binding sites near their transcriptional start sites (*SI Appendix*, Fig. S12*A*). These genes undergo circadian mRNA cycling in larval and adult muscle, similar to murine muscle (16), but became arrhythmic in mΔCLK (*SI Appendix*, Fig. S12 *B* and *C*). Furthermore, treatment with the Nr1d1/2 agonist during CT12-24 reduced the mRNA levels of *tsc1a*, *tsc1b,* and *tsc2* (*P* ≤ 0.041; *SI Appendix*, Fig. S12*D*). These results are consistent with increased TORC1 signaling and reduced autophagic flux after Nr1d1/2 activation/overexpression (Fig. 6 *D*–*F*). Conversely, the Nr1d1/2 antagonist induced a small rise in *tsc2* mRNA (*P* = 0.018; *SI Appendix*, Fig. S12*D*). Similarly, the flavone Nobiletin that activates Rora/c proteins, upregulated Tsc genes (*P* ≤ 0.015; *SI Appendix*, Figs. S11*D* and 12*D*). Moreover, Nr1d1/2 inhibition and Rora/c activation cooperate to increase nocturnal mRNA levels of Tsc and target genes (*SI Appendix*, Fig. S12 *A*, *D*, and *E*). Thus, increasing the Rora/c to Nr1d1/2 ratio may suppress muscle TORC1 activity at night to promote autophagy via enhanced Tsc levels.

### The Muscle Circadian Clock Restricts Growth at Night Through an Nr1d1–TORC1 Axis.

Supporting the role of TORC1 in nocturnal muscle growth enhancement via autophagy suppression, TORC1 activity was specifically elevated in mΔCLK muscle at night (Fig. 6*G*). Treatment with rapamycin reduced the extra growth during the subjective night in mΔCLK fish but had no such effect on control siblings (Fig. 6*H*). In contrast, during the subjective day, when rapamycin strongly reduces growth in siblings (11), it had little if any effect in mΔCLK fish (Fig. 6*H*). Together, these data strongly suggest mΔCLK represses autophagy via TORC1 activation at night in muscle.

In zebrafish muscle, *rorca/b* and *nr1d1/2* mRNAs rise in the late subjective day and late subjective night, respectively (*SI Appendix*, Fig. S2*B*), suggesting the activator protein will be high at night while the repressor protein will be high in the day (assuming a phase delay of ~6 h, as in murine muscle in ref.^6^). This aligns with the nocturnal peak and diurnal trough of Tsc mRNA levels in the muscle (*SI Appendix*, Fig. S12 *B* and *C*). After muscle clock inhibition, the *rorca/b* mRNA failed to rise in the late day (*SI Appendix*, Fig. S2*B*), reducing Rorc-mediated Tsc induction. In this situation, even a slight nocturnal increase in Nr1d1/2 mRNA/protein (*SI Appendix*, Fig. S2*B*) may be sufficient to suppress Tsc gene expression and partially activate TORC1.

We therefore tested whether altering Nr1d1/2 activity in mΔCLK fish could affect their abnormally high growth rate during the subjective night. While the Nr1d1 agonist had no effect, the Nr1d1 antagonist returned growth to its normal level at night (Fig. 6*I*). We conclude that the circadian variation in autophagy, and hence muscle growth, are maintained by the balance between Rora/c and Nr1d1/2 of the muscle peripheral clock (*SI Appendix*, Fig. S13).

## Discussion

The current study reports three major findings. First, that the muscle peripheral clock is required to promote nocturnal autophagy and UPS activity that initially limit larval muscle growth. Second, that this nocturnal catabolism is driven by the changing balance of Ror and Rev-erb transcription factors from the muscle clock stabilization loop, which act directly on CCGs and suppress TORC1 activity at night. Third, while zeitgebers such as feeding appear to mask the effects of loss of muscle clock function in later larval and young adult life, as fish with a defective muscle clock age their muscle homeostasis and continued growth falters. We conclude that the muscle peripheral clock is essential for the lifelong maintenance of muscle mass and function, at least in part by regulating proteolysis through MuRF and autophagy.

### The Muscle Peripheral Clock Promotes Nocturnal Protein Turnover That Limits Early Growth.

Before beginning to feed at 5 to 6 dpf, zebrafish larvae grow fast, increasing myotome volume by ~30%/day (11, 36). Nevertheless, a ~twofold increase in nocturnal muscle growth occurs when either the muscle peripheral clock, the UPS, or autophagy is inhibited, showing these processes each limit growth. Moreover, all three processes act even in anesthetized fish, showing that the increased nocturnal growth is not caused by altered muscle use, a confounder hitherto difficult to eliminate in other systems.

Mechanistically, the muscle clock activates both UPS and autophagy at night. Muscle exhibits a highly conserved catabolic signature with increased autophagy and proteasomal degradation in the fasting/inactive phase (4, 8, 11, 13–17), suggesting that the muscle peripheral clock may regulate this process throughout vertebrates. By eliminating movement and nutritional effects, our work reveals the direct function of the muscle clock in activating nocturnal protein turnover.

UPS and autophagy each contributed around half of de novo peptide degradation in fish larvae at night. Congruently, proteasomes and lysosomes are each responsible for 40 to 50% of protein breakdown in C2C12 myotubes in vitro (37). Strikingly, we observed no additive effect on growth when both pathways were blocked simultaneously, suggesting potential cross-talk between the pathways. Alternatively or additionally, homeostatic tissue scaling, as observed previously (38, 39), may prevent muscle outgrowing neighboring tissues.

How does the muscle clock control UPS? Proteasome number is not rate limiting for proteasomal degradation (40). Instead, the muscle clock drives proteasomal degradation, at least in part, through MuRFs. MuRFs are abundant muscle-specific E3 ubiquitin ligases that target sarcomeric proteins like actin and myosin for ubiquitination (41–43) and serve as biomarkers for muscle wasting (12). MuRF inhibition using Myomed reduced the abundance of ubiquitinated proteins at night and led to a nocturnal growth increase similar to that caused by proteasome blockade (11). As zebrafish contain, in addition to *murf1a* and *murf2b,* four other Murf genes, the expression of which are muscle-specific (22) but not characterized in detail, and as the specificity of Myomed and the anti-MuRF antibody to each MuRF protein are unknown, the MuRF/s involved remain to be determined. While other E3 ligases, such as Atrogin1 and Ubr4, are important for muscle UPS (12), it is clear that MuRF activity is rate limiting for growth in larvae.

Autophagy is equally rate limiting for growth, as inhibition of Ulk1 reduces autophagic flux and simultaneously increases nocturnal growth in larval muscle. Moreover, this Ulk1-dependent autophagy is also muscle clock dependent. Mechanistically, the loss of Ulk1^Ser757^ phosphorylation rhythm suggests that TORC1-mediated posttranslational modification of Ulk1 underpins the circadian regulation of autophagy. Supporting this, nocturnal inhibition of TORC1 reversed the effect of mΔCLK on growth by inducing autophagy. We previously showed that TORC1 signaling was diurnally activated in zebrafish larvae (11), an observation confirmed here under free-run independent of feeding. The TORC1 and autophagic rhythms are antiphasic and their reciprocity helps explain why larval muscle grows twice as fast in the active as in the inactive phase.

Global suppression of the circadian clock via *Δclk* mRNA injection resulted in reduced muscle growth during the day and increased growth at night (11). In contrast, muscle-specific clock inhibition solely enhanced muscle growth at night, suggesting that daytime muscle growth may be regulated by clocks in nonmuscle tissues. Further investigation is warranted to understand the role of different tissue clocks in the regulation of muscle growth.

### High Muscle Ror/Rev-erb Ratio Upregulates Nocturnal UPS and Autophagy.

Kinetic delays in the muscle clock stabilization loop appear to control the timing of nocturnal catabolism. As Rorc and Nr1d1/2 are dominant at night and day, respectively, their targets will be nocturnally activated and diurnally repressed. In mΔCLK larvae, the nocturnal dominance of Rorc activity may be lost due to decreased expression, leading to reduced nocturnal catabolism, a finding confirmed by reciprocal manipulation of the activity of Nr1d1 that reversed the effect of mΔCLK on nocturnal growth.

The circadian clock has been proposed to regulate autophagy rhythms through rhythmic transcription of *Ulk1* (44, 45). While zebrafish muscle also exhibits a circadian rhythm in *Ulk1* mRNA level that is muscle clock-dependent, Ulk1 protein continues to cycle in mΔCLK larvae. Instead, we find autophagy is cyclically regulated posttranslationally by TORC1, even under free-run conditions. In mΔCLK, Ulk1 is heavily phosphorylated at night, leading to reduced autophagy and increased growth.

How does the muscle clock regulate TORC1 activity? Rhythmic TORC1 signaling, peaking in the active phase, is highly conserved across species and cell types (11, 46–49). However, the cell-autonomous mechanisms driving circadian TORC1 activity are incompletely understood. One possibility is that circadian TORC1 activity is regulated via the TORC1 inhibitors Tsc1/2. We observed truly circadian, antiphasic oscillations in *Tsc1/2* mRNAs and TORC1 activity. While this observation is purely correlative, previous studies showed that overexpression of Rev-erbα decreases Tsc1 mRNA and protein levels while activating TORC1, whereas Rev-erbα knockdown elicited the opposite, in both cultured myotubes and hepatocytes (35, 50). The tumor suppressor genes *Tsc1/2* show haploinsufficient phenotypes (51–53), suggesting that even small quantitative changes in *Tsc1/2* mRNAs, as observed in both our fish and the mouse studies, could significantly impact TORC1 activity. Our current model proposes (*SI Appendix*, Fig. S13) that TORC1 activation suppresses protein degradation following muscle clock inhibition. Whether elevated TORC1 activity also enhances protein synthesis remains to be determined. However, we previously showed that TORC1 activity is normally not required for the muscle growth at night in WT larvae (11).

The core clock protein Per2 functions as a scaffold, tethering Tsc1 to suppress TORC1 activity in the mouse liver (54). As *per1b/2* mRNA rhythms were dampened after muscle clock inhibition, this dysregulation may contribute to ectopic activation of TORC1 in mΔCLK muscle. Although Tsc1/2 involvement is plausible, other TORC1 regulators, such as Akt and AMPK, which can also show circadian/diel rhythms in abundance/activity (46, 55–57), cannot be ruled out.

With respect to UPS, the clock controls *Murf1* transcription via Rev-erbα in mice (8, 58). Similarly, in zebrafish larvae, Nr1d1 antagonism and Rorc agonism cooperatively increased MuRF mRNAs, suggesting nocturnal MuRF induction is driven by a high Rorc/Nr1d1 balance at night. Alternatively or additionally, TORC1 activity reduces Atrogin1 and MuRF1 abundance (59) and protein ubiquitination (60). Thus, the circadian regulation of UPS and autophagy in muscle might converge at the level of TORC1 signaling.

### Muscle Clock Is Required to Prevent Premature Onset of Faltering Muscle Size and Function.

So far, our discussion has focused upon our findings in early larvae, in which the true effects of the muscle clock such as increased nocturnal muscle growth accompanied by reduced motile function, are revealed by our ability to remove potential zeitgebers such as diel variations in light, temperature, activity, and nutrition. We now consider the effects of muscle clock blockade in the presence of these zeitgebers.

Zebrafish embryos require 3 d of entrainment to establish transcriptional clock oscillation (61). Without entrainment, muscle size is reduced by 15% at 3 dpf (11). In mΔCLK embryos, an initial 5% reduction in myotome size at 3 dpf suggests that nonmuscle clocks support early myogenesis. This early defect in mΔCLK may reflect either Bmal1:Clock functions beyond circadian regulation (62–66), or a lack of entrainment in muscle precursor cells, which require the muscle clock for efficient terminal differentiation during adult muscle regeneration (67, 68). Once established in mature fibers, our data argue that the muscle clock autonomously restricts growth when nuclear addition is minimal (38).

When independent feeding commences at 5 to 6 dpf, the growth advantage of mΔCLK larvae is rapidly lost, suggesting that the combination of central clock entrainment and zeitgebers such as light and nutritional inputs restore diel variation in growth. Indeed, young mΔCLK fish grow normally in competition with their cohabiting WT siblings. Zebrafish grow throughout life, although their rate of growth decelerates with age (69, 70). Nonetheless, mΔCLK fish ceased to grow between age 12 to 24 months, and showed an absolute reduction in both standard weight, a parameter primarily controlled by muscle mass, and swimming speed (71, 72). These reductions conform to the definition of human sarcopenia as aging-related loss of muscle mass and function (73).

How might the lack of muscle clock cause such delayed defects in muscle function? Nocturnal protein degradation is not unique to developmental muscle growth, as it also occurs under homeostatic adult conditions, likely facilitating fiber repair or remodeling in response to changing use (17, 74, 75). As blocking the muscle clock and reducing protein degradation have similar effects, it is possible that accumulation of defective proteins in mΔCLK muscle leads to muscle dysfunction. In addition, the reduced growth of older mΔCLK fish compared to cohabiting WT siblings may partly result from feeding disadvantages due to impaired body or head muscle function. Similarly, young adult mice with muscle-specific deletion of Bmal1 exhibited reduced specific force, though premature sarcopenia was initially unreported (7, 76). However, sarcopenia manifests under free-run (DD) conditions (77), and mice entirely lacking *Bmal1* show severe premature muscle aging (3), which can be mitigated by *Bmal1* reconstitution in both brain and muscle but not muscle alone, likely due to disrupted feeding patterns (5). As a) TRF can restore muscle function in aging mice (5), b) autophagy-driven clearance of defective proteins dependent on the circadian clock is essential for lifelong health of muscle and other tissues and TRF-mediated longevity (4, 78, 79), and c) synchronous diurnal rhythms in autophagic and proteasomal activities are observed in mouse liver but remain unproven as direct circadian outputs due to persistence of zeitgebers (80), we hypothesize that reduced defective protein clearance contributes to aging-related muscle dysfunction.

To conclude, we establish a role of the muscle peripheral clock in the regulation of muscle protein degradation, further understanding of which may help mitigate the loss of muscle strength and mass that is associated with circadian disruptions resulted from sleep deprivation, night-shift, travel, and aging (81–84). As disease conditions often manifest in a time-dependent manner, and likewise kinetics of medications vary according to circadian rhythmicity, our work may contribute to the development of chronomedicine to treat debilitating muscle conditions with temporal precision to benefit patients.

### Limitations of Study.

The less pronounced changes in core clock gene mRNA rhythms observed in mΔCLK fish compared to null mice with a reconstituted brain clock (5) could have several possible explanations. a) The trunk/tail tissue we assayed contains nonmuscle tissues that likely maintain mRNA cycling. b) The mΔCLK transgene may not fully inhibit Bmal1:Clock function (85). c) Entrainment cues from brain and nonbrain clocks may combine to sustain transcriptional rhythms in larval zebrafish muscle. d) We have not interfered with muscle-intrinsic nontranscriptional rhythms (86). Our data thus reveal the minimal role for the transcriptional muscle clock.

Validation of our Ror/Nr1d1 balance model requires determination of how the levels and activity of Ror and Nr1d1/2 proteins correspond to those of their cycling mRNAs. Additionally, whereas both genetic and pharmacological manipulation of Ror/Nr1d1 balance mimics the effect of mΔCLK on nocturnal muscle growth, other muscle clock outputs may contribute. In future, it will be desirable to perform genetic tests of the cell autonomous mechanisms acting downstream of Ror/Nr1d1, perhaps through the use of tissue-specific CRISPants.

Although we report defective muscle function in both larvae and adult fish with a compromised muscle clock, the causes of muscle contractile dysfunction remain unclear; direct evidence of a molecular defect in sarcomeric proteins or metabolism in mΔCLK fish muscle is needed. We note that metabolomic changes dependent on the muscle clock have been found in mice (see Introduction). As marked diel cycles in Rorc/Nr1d1 ratio and *tsc1a/2* mRNAs present in muscle are reduced or lost in adult mΔCLK fish, it remains likely that gradual accumulation of defective proteins leads to the muscle dysfunction. However, we cannot eliminate the possibility of a “developmental” defect that predisposes mΔCLK fish to sarcopenia. Nevertheless, the reduction in standard weight suggests a specific muscle defect; this effect was most pronounced in males within the analyzed cohort, which is strikingly congruent with our recent discovery of greater muscle mass decline in males in the UK population (87).

## Methods

Details of materials (*SI Appendix*, Table S2), most methods and associated justifications, are included within *SI Appendix* on the journal website.

### Zebrafish Experimentation.

Zebrafish embryos (injected or not) were entrained on a 12/12 h light/dark cycle before entering experiments at 3 dpf (11), unless otherwise specified. We distinguish Zeitgeber Time (ZT) when zeitgebers are present from Circadian Time (CT) when, after entrainment, zeitgebers are removed and fish are reared under constant conditions. Larvae were treated and analyzed as described in *SI Appendix*, *Supplementary Materials and Methods*. All experiments were performed in accordance with licenses held under the UK Animals (Scientific Procedures) Act 1986.

### RNA and Protein Detection.

RNA and protein were collected, prepared, and detected as previously described (11, 72). See replicate western blots in *SI Appendix*, Figs. S14–S22.

### Statistical Analyses.

Statistical analyses were conducted using GraphPad Prism, R, and RStudio. A significance threshold of *P* < 0.05 was used to reject null hypotheses. *P*-values smaller than 0.05 were supplemented with False Positive Risk (88) calculation using the R package “pcal” (*SI Appendix*, Table S3 and Figs. S23–S40).

## Supplementary Material

Appendix 01 (PDF)

## Data Availability

All study data are included in the article and/or *SI Appendix* and numerical data were deposited on Figshare accessible at https://doi.org/10.6084/m9.figshare.28841054 (89).

## References

[1] E. N. C. Manoogian, S. Panda, Circadian rhythms, time-restricted feeding, and healthy aging. Ageing Res. Rev. **39**, 59–67 (2017). 28017879 10.1016/j.arr.2016.12.006 PMC5814245

[2] A. Klarsfeld, F. Rouyer, Effects of circadian mutations and LD periodicity on the life span of *Drosophila melanogaster*. J. Biol. Rhythms **13**, 471–478 (1998). 9850008 10.1177/074873098129000309

[3] R. V. Kondratov, A. A. Kondratova, V. Y. Gorbacheva, O. V. Vykhovanets, M. P. Antoch, Early aging and age-related pathologies in mice deficient in BMAL1, the core componentof the circadian clock. Genes Dev. **20**, 1868–1873 (2006). 16847346 10.1101/gad.1432206 PMC1522083

[4] M. Ulgherait , Circadian autophagy drives iTRF-mediated longevity. Nature **598**, 353–358 (2021). 34588695 10.1038/s41586-021-03934-0 PMC9395244

[5] A. Kumar , Brain-muscle communication prevents muscle aging by maintaining daily physiology. Science **384**, 563–572 (2024). 38696572 10.1126/science.adj8533

[6] J. J. Kelu, "Circadian Rhythms in Muscle Health and Diseases" in International Review of Cell and Molecular Biology (Academic Press, 2024). 10.1016/bs.ircmb.2024.10.002 40390463

[7] K. A. Dyar , Muscle insulin sensitivity and glucose metabolism are controlled by the intrinsic muscle clock. Mol. Metab. **3**, 29–41 (2014). 24567902 10.1016/j.molmet.2013.10.005 PMC3929910

[8] K. A. Dyar , Transcriptional programming of lipid and amino acid metabolism by the skeletal muscle circadian clock. PLoS Biol. **16**, e2005886 (2018). 30096135 10.1371/journal.pbio.2005886 PMC6105032

[9] B. D. Harfmann , Muscle-specific loss of leads to disrupted tissue glucose metabolism and systemic glucose homeostasis. Skelet. Muscle **6**, s13395 (2016). 10.1186/s13395-016-0082-x PMC4969979 27486508

[10] J. G. Smith , Liver and muscle circadian clocks cooperate to support glucose tolerance in mice. Cell Rep. **42**, 112588 (2023). 37267101 10.1016/j.celrep.2023.112588 PMC10592114

[11] J. J. Kelu, T. G. Pipalia, S. M. Hughes, Circadian regulation of muscle growth independent of locomotor activity. Proc. Natl. Acad. Sci. U.S.A. **117**, 31208–31218 (2020). 33229575 10.1073/pnas.2012450117 PMC7733834

[12] D. C. Hughes, C. A. Goodman, L. M. Baehr, P. Gregorevic, S. C. Bodine, A critical discussion on the relationship between E3 ubiquitin ligases, protein degradation, and skeletal muscle wasting: It’s not that simple. Am. J. Physiol. Cell Physiol. **325**, C1567–C1582 (2023). 37955121 10.1152/ajpcell.00457.2023 PMC10861180

[13] I. P. Amaral, I. A. Johnston, Circadian expression of clock and putative clock-controlled genes in skeletal muscle of the zebrafish. Am. J. Physiol. Regul Integr Comp. Physiol. **302**, R193–206 (2012). 22031781 10.1152/ajpregu.00367.2011

[14] L. Perrin , Transcriptomic analyses reveal rhythmic and CLOCK-driven pathways in human skeletal muscle. eLife **7**, e34114 (2018). 29658882 10.7554/eLife.34114 PMC5902165

[15] K. A. Dyar , Atlas of circadian metabolism reveals system-wide coordination and communication between clocks. Cell **174**, 1571–1585.e1511 (2018). 30193114 10.1016/j.cell.2018.08.042 PMC6501776

[16] C. A. Wolff , Defining the age-dependent and tissue-specific circadian transcriptome in male mice. Cell Rep. **42**, 111982 (2023). 36640301 10.1016/j.celrep.2022.111982 PMC9929559

[17] M. Wirianto , The GSK-3beta-FBXL21 axis contributes to circadian TCAP degradation and skeletal muscle function. Cell Rep. **32**, 108140 (2020). 32937135 10.1016/j.celrep.2020.108140 PMC8299398

[18] R. Sartori, V. Romanello, M. Sandri, Mechanisms of muscle atrophy and hypertrophy: Implications in health and disease. Nat. Commun. **12**, 330 (2021). 33436614 10.1038/s41467-020-20123-1 PMC7803748

[19] J. P. Leduc-Gaudet , MYTHO is a novel regulator of skeletal muscle autophagy and integrity. Nat. Commun. **14**, 965–967 (2023). 36864049 10.1038/s41467-023-36817-1 PMC9981687

[20] B. Thisse, C. Thisse, Fast Release Clones: A High Throughput Expression Analysis. ZFIN Direct Data Submission (http://zfin.org/ZDB-PUB-040907-1#summary). Accessed 30 October 2024.

[21] S. M. Kisia, Structure of Fish Locomotory Muscle. Ed. 1 (1996).

[22] B. Li, S. Li, Q. He, S. Du, Generation of MuRF-GFP transgenic zebrafish models for investigating murf gene expression and protein localization in Smyd1b and Hsp90alpha1 knockdown embryos. Comp. Biochem. Physiol. B Biochem. Mol. Biol. **240**, 110368 (2020). 31669374 10.1016/j.cbpb.2019.110368 PMC6934922

[23] H. Shimizu , The Calcineurin-FoxO-MuRF1 signaling pathway regulates myofibril integrity in cardiomyocytes. eLife **6**, e27955 (2017) 28826496 10.7554/eLife.27955 PMC5576919

[24] J. Fielitz , Myosin accumulation and striated muscle myopathy result from the loss of muscle RING finger 1 and 3. J. Clin. Invest. **117**, 2486–2495 (2007). 17786241 10.1172/JCI32827 PMC1957544

[25] C. C. Witt , Cooperative control of striated muscle mass and metabolism by MuRF1 and MuRF2. EMBO J. **27**, 350–360 (2008). 18157088 10.1038/sj.emboj.7601952 PMC2168395

[26] D. Mauvoisin, F. Gachon, Proteomics in circadian biology. J. Mol. Biol. **432**, 3565–3577 (2020). 31843517 10.1016/j.jmb.2019.12.004

[27] A. Stangherlin, E. Seinkmane, J. S. O’Neill, Understanding circadian regulation of mammalian cell function, protein homeostasis, and metabolism. Curr. Opin. Syst. Biol. **28**, 34950808 (2021). 10.1016/j.coisb.2021.100391 PMC8660647 34950808

[28] M. Chivet , Polyglutamine-expanded androgen receptor alteration of skeletal muscle homeostasis and myonuclear aggregation are affected by sex age and muscle metabolism. Cells **9**, 325 (2020). 32019272 10.3390/cells9020325 PMC7072234

[29] H. Doi , p62/SQSTM1 differentially removes the toxic mutant androgen receptor via autophagy and inclusion formation in a spinal and bulbar muscular atrophy mouse model. J. Neurosci. **33**, 7710–7727 (2013). 23637164 10.1523/JNEUROSCI.3021-12.2013 PMC6618982

[30] I. Nemazanyy , Defects of Vps15 in skeletal muscles lead to autophagic vacuolar myopathy and lysosomal disease. EMBO Mol. Med. **5**, 870–890 (2013). 23630012 10.1002/emmm.201202057 PMC3779449

[31] E. Masiero , Autophagy is required to maintain muscle mass. Cell Metab. **10**, 507–515 (2009). 19945408 10.1016/j.cmet.2009.10.008

[32] P. Dierickx , SR9009 has REV-ERB-independent effects on cell proliferation and metabolism. Proc. Natl. Acad. Sci. U.S.A. **116**, 12147–12152 (2019). 31127047 10.1073/pnas.1904226116 PMC6589768

[33] B. M. Forman , Cross-talk among ROR alpha 1 and the Rev-erb family of orphan nuclear receptors. Mol. Endocrinol. **8**, 1253–1261 (1994). 7838158 10.1210/mend.8.9.7838158

[34] J. B. Mannick, D. W. Lamming, Targeting the biology of aging with mTOR inhibitors. Nat. Aging **3**, 642–660 (2023). 37142830 10.1038/s43587-023-00416-y PMC10330278

[35] M. Dadon-Freiberg, N. Chapnik, O. Froy, REV-ERBα activates the mTOR signalling pathway and promotes myotubes differentiation. Biol. Cell **112**, 213–221 (2020). 32306421 10.1111/boc.201900091

[36] Y. Hinits , Defective cranial skeletal development, larval lethality and haploinsufficiency in Myod mutant zebrafish. Dev. Biol. **358**, 102–112 (2011). 21798255 10.1016/j.ydbio.2011.07.015 PMC3360969

[37] J. Zhao , FoxO3 coordinately activates protein degradation by the Autophagic/Lysosomal and proteasomal pathways in atrophying muscle cells. Cell Metab. **6**, 472–483 (2007). 18054316 10.1016/j.cmet.2007.11.004

[38] S. D. Roy , Myotome adaptability confers developmental robustness to somitic myogenesis in response to fibre number alteration. Dev. Biol. **431**, 321–335 (2017). 28887016 10.1016/j.ydbio.2017.08.029 PMC5667637

[39] K. Ishimatsu , Size-reduced embryos reveal a gradient scaling-based mechanism for zebrafish somite formation. Development **145**, dev161257 (2018). 29769221 10.1242/dev.161257 PMC6031319

[40] G. A. Collins, A. L. Goldberg, The logic of the 26S proteasome. Cell **169**, 792–806 (2017). 28525752 10.1016/j.cell.2017.04.023 PMC5609836

[41] S. Cohen , During muscle atrophy, thick, but not thin, filament components are degraded by MuRF1-dependent ubiquitylation. J. Cell Biol. **185**, 1083–1095 (2009). 19506036 10.1083/jcb.200901052 PMC2711608

[42] B. A. Clarke , The E3 ligase MuRF1 degrades myosin heavy chain protein in dexamethasone-treated skeletal muscle. Cell Metab. **6**, 376–385 (2007). 17983583 10.1016/j.cmet.2007.09.009

[43] C. Polge , Muscle actin is polyubiquitinylated and targeted for breakdown by the E3 ligase MuRF1. FASEB J. **25**, 3790–3802 (2011). 21764995 10.1096/fj.11-180968

[44] G. Huang, F. Zhang, Q. Ye, H. Wang, The circadian clock regulates autophagy directly through the nuclear hormone receptor Nr1d1/Rev-erbalpha and indirectly via Cebpb/(C/ebpbeta) in zebrafish. Autophagy **12**, 1292–1309 (2016). 27171500 10.1080/15548627.2016.1183843 PMC4968235

[45] E. Woldt , Rev-erb-α modulates skeletal muscle oxidative capacity by regulating mitochondrial biogenesis and autophagy. Nat. Med. **19**, 1039-1046+e2 (2013). 23852339 10.1038/nm.3213 PMC3737409

[46] C. Jouffe , The circadian clock coordinates ribosome biogenesis. PLoS Biol. **11**, e1001455 (2013) 23300384 10.1371/journal.pbio.1001455 PMC3536797

[47] S. W. Chang, T. Yoshihara, S. Machida, H. Naito, Circadian rhythm of intracellular protein synthesis signaling in rat cardiac and skeletal muscles. Biochem. Biophys. Rep. **9**, 153–158 (2017). 28956001 10.1016/j.bbrep.2016.12.005 PMC5614553

[48] M. Cornu , Hepatic mTORC1 controls locomotor activity, body temperature, and lipid metabolism through FGF21. Proc. Natl. Acad. Sci. U.S.A. **111**, 11592–11599 (2014). 25082895 10.1073/pnas.1412047111 PMC4136616

[49] R. V. Khapre , Metabolic clock generates nutrient anticipation rhythms in mTOR signaling. Aging (Albany NY) **6**, 675–689 (2014). 25239872 10.18632/aging.100686 PMC4169861

[50] M. Dadon-Freiberg, N. Chapnik, O. Froy, REV-ERBα alters circadian rhythms by modulating mTOR signaling. Mol. Cell Endocrinol. **521**, 33285244 (2021). 10.1016/j.mce.2020.111108 33285244

[51] D. Ehninger , Reversal of learning deficits in a mouse model of tuberous sclerosis. Nat. Med. **14**, 843–848 (2008). 18568033 10.1038/nm1788 PMC2664098

[52] C. Alquezar , TSC1 loss increases risk for tauopathy by inducing tau acetylation and preventing tau clearance via chaperone-mediated autophagy. Sci. Adv. **7**, eabg3897 (2021). 34739309 10.1126/sciadv.abg3897 PMC8570595

[53] S. H. Kim, C. K. Speirs, L. Solnica-Krezel, K. C. Ess, Zebrafish model of tuberous sclerosis complex reveals cell-autonomous and non-cell-autonomous functions of mutant tuberin. Dis. Model Mech **4**, 255–U142 (2011). 20959633 10.1242/dmm.005587 PMC3046101

[54] R. Wu , The circadian protein period2 suppresses mTORC1 activity via recruiting Tsc1 to mTORC1 complex. Cell Metab. **29**, 653-+ (2019). 30527742 10.1016/j.cmet.2018.11.006

[55] R. Aviram, V. Dandavate, G. Manella, M. Golik, G. Asher, Ultradian rhythms of AKT phosphorylation and gene expression emerge in the absence of the circadian clock components Per1 and Per2. PLoS Biol. **19**, e3001492 (2021). 34968386 10.1371/journal.pbio.3001492 PMC8718012

[56] J. H. Um , AMPK regulates circadian rhythms in a tissue- and isoform-specific manner. PLoS One **6**, e18450 (2011). 21483791 10.1371/journal.pone.0018450 PMC3069094

[57] K. A. Lamia , AMPK regulates the circadian clock by cryptochrome phosphorylation and degradation. Science **326**, 437–440 (2009). 19833968 10.1126/science.1172156 PMC2819106

[58] A. Mayeuf-Louchart , Rev-erb-α regulates atrophy-related genes to control skeletal muscle mass. Sci. Rep. **7**, 14383 (2017). 29085009 10.1038/s41598-017-14596-2 PMC5662766

[59] Y. Nishimura , Ubiquitin E3 ligase atrogin-1 protein is regulated via the rapamycin-sensitive mTOR-S6K1 signaling pathway in C2C12 muscle cells. Am. J. Physiol. Cell Physiol. **323**, C215–C225 (2022). 35704697 10.1152/ajpcell.00384.2021

[60] J. H. Zhao, B. Zhai, S. P. Gygi, A. L. Goldberg, mTOR inhibition activates overall protein degradation by the ubiquitin proteasome system as well as by autophagy. Proc. Natl. Acad. Sci. U.S.A. **112**, 15790–15797 (2015). 26669439 10.1073/pnas.1521919112 PMC4703015

[61] M. P. S. Dekens , Light regulates the cell cycle in zebrafish. Curr. Biol. **13**, 2051–2057 (2003). 14653994 10.1016/j.cub.2003.10.022

[62] J. D. Alvarez, A. Sehgal, The thymus is similar to the testis in its pattern of circadian clock gene expression. J. Biol. Rhythms **20**, 111–121 (2005). 15834108 10.1177/0748730404274078

[63] D. Morse, N. Cermakian, S. Brancorsini, M. Parvinen, P. Sassone-Corsi, No circadian rhythms in testis: Expression is independent and developmentally regulated in the mouse. Mol. Endocrinol. **17**, 141–151 (2003). 12511614 10.1210/me.2002-0184

[64] A. A. H. Ali , Impact of targeted deletion of the circadian clock gene Bmal1 in excitatory forebrain neurons on adult neurogenesis and olfactory function. Int. J. Mol. Sci. **21**, 1394 (2020). 32092990 10.3390/ijms21041394 PMC7073072

[65] A. Malik, R. V. Kondratov, R. J. Jamasbi, M. E. Geusz, Circadian clock genes are essential for normal adult neurogenesis, differentiation, and fate determination. PLoS ONE **10**, e0139655 (2015). 26439128 10.1371/journal.pone.0139655 PMC4595423

[66] B. V. Lananna , Cell-autonomous regulation of astrocyte activation by the circadian clock protein BMAL1. Cell Rep. **25**, 1-9+e5 (2018). 30282019 10.1016/j.celrep.2018.09.015 PMC6221830

[67] N. Katoku-Kikyo , Per1/Per2-Igf2 axis-mediated circadian regulation of myogenic differentiation. J. Cell Biol. **220**, e202101057 (2021). 34009269 10.1083/jcb.202101057 PMC8138781

[68] P. Zhu , BMAL1 drives muscle repair through control of hypoxic NAD regeneration in satellite cells. Genes Dev. **36**, 149–166 (2022). 35115380 10.1101/gad.349066.121 PMC8887128

[69] C. Singleman, N. G. Holtzman, Growth and maturation in the zebrafish: A staging tool for teaching and research. Zebrafish **11**, 396–406 (2014). 24979389 10.1089/zeb.2014.0976 PMC4108942

[70] P. Gómez-Requeni, L. E. C. Conceiçao, A. E. O. Jordal, I. Ronnestad, A reference growth curve for nutritional experiments in zebrafish (Danio rerio) and changes in whole body proteome during development. Fish Physiol. Biochem. **36**, 1199–1215 (2010). 20432063 10.1007/s10695-010-9400-0

[71] E. Kague , Scleraxis genes are required for normal musculoskeletal development and for rib growth and mineralization in zebrafish. FASEB J. **33**, 9116–9130 (2019). 31100023 10.1096/fj.201802654RR PMC6662971

[72] M. Ganassi , Myogenin promotes myocyte fusion to balance fibre number and size. Nat. Commun. **9**, 4232 (2018). 30315160 10.1038/s41467-018-06583-6 PMC6185967

[73] A. J. Cruz-Jentoft, A. A. Sayer, Sarcopenia. Lancet **393**, 2636–2646 (2019). 31171417 10.1016/S0140-6736(19)31138-9

[74] L. A. Riley , The skeletal muscle circadian clock regulates titin splicing through RBM20. eLife **11**, e76478 (2022). 36047761 10.7554/eLife.76478 PMC9473687

[75] J. Y. Lim , The circadian E3 ligase FBXL21 regulates myoblast differentiation and sarcomere architecture via MYOZ1 ubiquitination and NFAT signaling. PLoS Genet. **18**, e1010574 (2022). 36574402 10.1371/journal.pgen.1010574 PMC9829178

[76] E. A. Schroder , Intrinsic muscle clock is necessary for musculoskeletal health. J. Physiol. **593**, 5387–5404 (2015). 26486627 10.1113/JP271436 PMC4704520

[77] J. Fernandez-Martinez , iMS-Bmal1(-/-) mice show evident signs of sarcopenia that are counteracted by exercise and melatonin therapies. J. Pineal Res. **76**, e12912 (2024). 37702245 10.1111/jpi.12912

[78] F. Demontis, N. Perrimon, FOXO/4E-BP signaling in *Drosophila* muscles regulates organism-wide proteostasis during aging. Cell **143**, 813–825 (2010). 21111239 10.1016/j.cell.2010.10.007 PMC3066043

[79] F. Demontis, R. Piccirillo, A. L. Goldberg, N. Perrimon, Mechanisms of skeletal muscle aging: Insights from *Drosophila* and mammalian models. Disease Models Mech. **6**, 1339–1352 (2013). 10.1242/dmm.012559 PMC3820258 24092876

[80] M. Ryzhikov , Diurnal rhythms spatially and temporally organize autophagy. Cell Rep. **26**, 1880–1892.e1886 (2019). 30759397 10.1016/j.celrep.2019.01.072 PMC6442472

[81] H. Zhang, J. Liang, N. Chen, Do not neglect the role of circadian rhythm in muscle atrophy. Ageing Res. Rev. **63**, 101155 (2020). 32882420 10.1016/j.arr.2020.101155

[82] J. A. Vitale, M. Bonato, A. La Torre, G. Banfi, The role of the molecular clock in promoting skeletal muscle growth and protecting against sarcopenia. Int. J. Mol. Sci. **20**, 4318 (2019). 31484440 10.3390/ijms20174318 PMC6747101

[83] D. Malhan, M. Yalçin, B. Schoenrock, D. Blottner, A. Relógio, Skeletal muscle gene expression dysregulation in long-term spaceflights and aging is clock-dependent. Npj Microgravity **9**, 30 (2023). 37012297 10.1038/s41526-023-00273-4 PMC10070655

[84] Y. I. Choi , Circadian rhythm disruption is associated with an increased risk of sarcopenia: A nationwide population-based study in Korea. Sci. Rep. **9**, 12015 (2019). 31427694 10.1038/s41598-019-48161-w PMC6700184

[85] Z. Ben-Moshe Livne , Genetically blocking the zebrafish pineal clock affects circadian behavior. PLoS Genet. **12**, e1006445 (2016). 27870848 10.1371/journal.pgen.1006445 PMC5147766

[86] D. C. S. Wong, J. S. O’Neill, Non-transcriptional processes in circadian rhythm generation. Curr. Opin. Physiol. **5**, 117–132 (2018). 30596188 10.1016/j.cophys.2018.10.003 PMC6302373

[87] T. W. Fieldsend , Sexual dimorphism in human muscle ageing. medRxiv [Preprint] (2025). 10.1101/2025.01.06.25319958 (Accessed 1 March 2025).

[88] T. Sellke, M. J. Bayarri, J. O. Berger, Calibration of p-values for testing precise null hypotheses. Am. Statis. **55**, 62–71 (2001).

[89] J. J. Kelu, S. M. Hughes, Data from “Muscle peripheral circadian clock drives nocturnal protein degradation via raised Ror/Rev-erb balance and prevents premature sarcopenia.” Figshare. 10.6084/m9.figshare.28841054. Deposited 22 April 2025. PMC12088385 40324095

