# Supplementary material for: Muscle peripheral circadian clock drives nocturnal protein degradation via raised Ror/Rev-erb balance and prevents premature sarcopenia
Source: Proc Natl Acad Sci U S A. 2025 May 5;122(19):e2422446122. doi: 10.1073/pnas.2422446122 (PMC12088385; doi:10.1073/pnas.2422446122)
Supplement: Supplementary file 1 — Appendix 01 (PDF) [file pnas.2422446122.sapp.pdf]

PNAS

Supporting Information for

Muscle peripheral circadian clock drives nocturnal protein degradation through raised Ror/Rev-erb balance and prevents premature sarcopenia

Jeffrey J. Kelu[1*] and Simon M. Hughes[1*]

[1] Randall Centre for Cell and Molecular Biophysics, King's College London, London, SE1 1UL, UK

[*]Correspondence: jeffrey.kelu@kcl.ac.uk; s.hughes@kcl.ac.uk

This PDF file includes:

Supplementary Materials and Methods: pg 2-13

Figures S1 to S40: pg 14-61

Tables S1 to S3: pg 62-85

SI References: pg 86-88

Supplementary Materials and Methods

Zebrafish husbandry

Zebrafish were reared at 28.5ºC on a 14/10 hours light/dark cycle, with staging and husbandry as described (1). For experiments, newly-fertilised embryos were obtained by pairwise natural spawning, cleaned and transferred within 1-2 h to incubators at 28.5ºC fitted with timed LED lighting systems on a 12/12 hours light/dark (LD) cycle, or other regimes as described in text. No more than 80 fish were present in each 100 mm dish, and chorion debris, dead or sick fish were removed daily where the light regime permitted. The intensity of light and temperature in the incubator were maintained and monitored and circadian entrainment performed by exposure to light cycles from 0-3 dpf as described previously (2). Specifically, we distinguish Zeitgeber Time (ZT) when zeitgebers such as light or feeding cycles are present from Circadian Time (CT) when, after entrainment, zeitgebers are removed and fish are reared under constant conditions. All experiments were performed in accordance with licences held under the UK Animals (Scientific Procedures) Act 1986 and later modifications and conforming to all relevant guidelines and regulations.

Zebrafish lines

All zebrafish strains used were on the AB wild-type background. *Tg(actc1b:mCherryCAAX)*^{pc22} (3), *Tg(actc1b:EGFP)*^{zf13} (4), and *Tg(CMV:EGFP-map1lc3b)*^{zf155} (5) were described previously. *Tg(actc1b:LIFEACT-mCherry)*^{kg331}, *Tg(actc1b:EGFP-2A-ΔCLK-5xMyc)*^{kg332}, *Tg(actc1b:EGFP-2A-ΔCLK-5xMyc)*^{kg333}, and *Tg(actc1b:mCherry-2A-ΔCLK-5xMyc)*^{kg334} were derived in this study as described below.

RNA extraction, cDNA preparation, and qPCR

Total RNA was extracted from a pool of 5 larvae per biological replicate (either live or snap-frozen in liquid nitrogen) using Tri-reagent (Sigma-Aldrich) via sonication on ice. In some experiments, larvae were anesthetized and then decapitated using scalpel, and RNA collected from a pool of 5-6 isolated trunk/tail per lay. RNA was isolated via phase separation using 1-bromo-3-chloropropane, precipitated using isopropanol, and washed using 75% ethanol, or purified using commercial columns following manufacturer's instructions (Qiagen). Extracted RNA was then treated with DNase (Promega or Qiagen) for 30 min at 37ºC. Purified total RNA (500 ng) was subsequently reverse transcribed in the presence of RNase inhibitor (NEB) using High-Capacity cDNA Reverse Transcription Kit (Applied Biosystems) following manufacturer's instructions using both random and oligo-dT primers (Invitrogen).

For qPCR, technical triplicates were performed on 5 ng of cDNA using Takyon Low ROX SYBR 2X MasterMix blue dTTP (Eurogentec) using either ViiA™ 7 or QuantStudio™ 7 Flex Real-Time PCR System (Thermo Fisher Scientific).

Oligonucleotide primers listed in Table S2 were customed-designed using OligoArchitect™ (Sigma-Aldrich) or Primer-BLAST (NIH), adopted from previous studies, or ordered directly from KiCqStart® SYBR® Green Primers (Sigma-Aldrich).

Molecular cloning

For all cloning procedures, high-fidelity HS VeriFi DNA Polymerase (PCRBIO), NEBuilder® HiFi DNA Assembly Master Mix, restriction enzymes, and purification kits were used according to manufacturer's instruction. Transformation was done using either NEB® 5-alpha Competent *E. coli* (High Efficiency) or Mix & Go Competent Cells - Strain DH5 Alpha (Zymo Research).

To generate *pT2-actc1b:EGFP-2A-ΔCLK-5xMyc*, a sequence containing *actc1b* promoter was first cloned from *pT2-actc1b:mCherryCAAX* (3) via PCR. The vector backbone *pT2-UAS:EGFP-2A-ΔCLK-5xMyc* (6) was linearised using AgeI and XhoI to exclude the *UAS* promoter sequence and then ligated with the *actc1b* promoter. To create *pT2-actc1b:mCherry-2A-ΔCLK-5xMyc*, the *EGFP* fragment in *pT2-actc1b:EGFP-2A-ΔCLK-5xMyc* was replaced with an *mCherry* fragment cloned from *pT2-actc1b:mCherryCAAX* (3).

To generate *pT2-actc1b:mCherry-2A-nr1d1(FL)-6xHis*, a fragment containing the full-length (FL) coding sequence of *nr1d1* (1914-bp) was first cloned from a cDNA library (prepared from wildtype AB larvae at 4 dpf) via PCR by adding a *6xHis* sequence to the 3' end. The *ΔCLK-5xMyc* fragment in *pT2-actc1b:mCherry-2A-ΔCLK-5xMyc* was then replaced with the *nr1d1(FL)-6xHis* fragment. *pT2-actc1b:mCherry-2A-nr1d1(Mut)-6xHis* that contains a large (1824-bp) in-frame deletion in the coding sequence resulted from a cloning error.

To generate *pT2-actc1b:mCherry-2A-rorca-6xHis* and *pT2-actc1b:mCherry-2A-rorcb-6xHis*, fragments containing the full-length coding sequence of *rorca* (1440-bp) and *rorcb* (1443-bp) were cloned from the cDNA library as described above via PCR by adding a *6xHis* sequence to the 3' end. The *ΔCLK-5xMyc* fragment in *pT2-actc1b:mCherry-2A-ΔCLK-5xMyc* was then replaced with the *rorca/rorcb-6xHis* fragment.

To generate *pT2-actc1b:LIFEACT-mCherry*, a *pT2-actc1b:LIFEACT-EGFP* vector was first created by replacing the *mCherryCAAX* sequence of the *pT2-actc1b:mCherryCAAX* (3) with a *LIFEACT-EGFP* fragment that was cloned from *pME-LIFEACT-EGFP* (3). The *EGFP* sequence within the *pT2-actc1b:LIFEACT-EGFP* was eventually replaced with an *mCherry* fragment cloned from *pT2-actc1b:mCherryCAAX* (3).

To generate *pCS-mCherry-p62*, a *pCS-mCherry* vector is first created by deleting the *CAAX* sequence from the *pCS-mCherryCAAX* backbone (a gift from Henry Roehl). Then a fragment containing the full-length coding sequence of *p62* (1821-bp) was cloned from the cDNA library

as described above via PCR. The *p62*-containing fragment was subsequently fused in-frame with the 3' end of the *mCherry* sequence of the the *pCS-mCherry* vector.

All sequences were processed using the software ApE or SnapGene. Primers were designed using Primer-BLAST (NIH) and NEBuilder (NEB) and are listed in Table S2. Sequence integrity of all constructs was confirmed by Sanger sequencing (Genewiz).

Transgene expression

To overexpress a transgene in myofibres mosaically, around 1 nL containing 25 pg of either *pT2-actc1b:EGFP-2A-ΔCLK-5xMyc,* *pT2-actc1b:mCherry-2A-nr1d1(FL)-6xHis*, *pT2-actc1b:mCherry-2A-nr1d1(Mut)-6xHis*), *pT2-actc1b:mCherry-2A-rorca-6xHis*, or *pT2-actc1b:mCherry-2A-rorcb-6xHis* plasmid was injected into one-cell stage wildtype AB embryos. Injected embryos were then screened for EGFP/mCherry fluorescence and low mosaicism in the trunk musculature to enable direct comparison with neighbouring fluorescence-negative control fibres before being subjected to experimental procedures. For Nr1d1 overexpression experiments, *pT2-actc1b:mCherry-2A-nr1d1(Mut)-6xHis* was used as a control whenever possible.

To overexpress *mCherry-p62* in zebrafish larvae ubiquitously, capped *mCherry-p62* mRNA was first *in vitro* transcribed from NotI-linearised *pCS-mCherry-p62* plasmid using mMESSAGE mMACHINE™ SP6 Transcription Kit (Invitrogen) and RNase inhibitor (NEB). Around 2 nL containing 900 pg of the mRNA was then injected into one-cell stage embryos. Injected embryos with strong overall mCherry fluorescence were used in experiments.

Generation of transgenic lines

Tol2-mediated transgenesis was utilised to generate various transgenic lines. Capped *Tol2* mRNA was *in vitro* transcribed as described above using the SP6 promotor of NotI-linearised *pCS2FA-transposase* plasmid (a gift from Claudia Linker). Around 1 nL containing 25 pg of either the *pT2-actc1b-EGFP-2A-ΔCLK-5xMyc*, *pT2-actc1b:mCherry-2A-ΔCLK-5xMyc*, or *pT2-actc1b:LIFEACT-mCherry* plasmid was co-injected with 25 pg of *Tol2* mRNA into one-cell stage wildtype AB embryos. Injected embryos were then screened for strong EGFP/mCherry fluorescence and high mosaicism in the trunk musculature and raised to adulthood (F0).

Transgenic F0 founders were then outcrossed with wildtype AB fish to isolate germline transmitted F1 progeny. Stable F2 lines (*kg331Tg*, *kg332Tg*, *kg333Tg*, *kg334Tg,* and *kg335Tg*) were established by outcrossing a single F1 transmitter again with wildtype AB fish, and maintained as heterozygotes thereafter. All heterozygous transgenic outcrosses produced transgenic and wildtype progeny in ~1:1 ratio, indicating a single transgene integration locus in these stable lines. Heterozygote transgenic fish showed no developmental abnormalities,

survived to adulthood, and were fertile. Experiments were performed using F2 or later generations, unless otherwise specified.

Tg(actc1b:LIFEACT-mCherry)^{kg331Tg} was the single line isolated. *Tg(actc1b:EGFP-2A-ΔCLK-5xMyc)^{kg332Tg}* and *Tg(actc1b:EGFP-2A-ΔCLK-5xMyc)^{kg333Tg}* were raised from F1 transmitters obtained from two different F0 founders; whereas *Tg(actc1b:mCherry-2A-ΔCLK-5xMyc)^{kg334Tg}* and *Tg(actc1b:mCherry-2A-ΔCLK-5xMyc)^{kg335Tg}*, were different F1 transmitters obtained from the same F0 founder.

Transgene mapping

Transgene mapping of the *Tg(actc1b:EGFP-2A-ΔCLK-5xMyc)^{kg333}* was performed as described (7). Briefly, genomic DNA was extracted from a pool of *Tg(actc1b:EGFP-2A-ΔCLK-5xMyc)^{kg333}* larvae at 4 dpf by treating with proteinase K at 200 µg/µL (Fisher BioReagents) for 3 h at 50ºC in DirectPCR Lysis Reagent (Viagen Biotech). Genomic DNA was then isolated using phenol/chloroform/isoamyl alcohol (25:24:1), precipitated using isopropanol, washed using 75% ethanol, and resuspended in MilliQ water. 1 µg of purified genomic DNA was then digested with either SalI or EcoRV for at least 3 h at 37ºC in a reaction volume of 30 µL. SalI and EcoRV were chosen because restriction sites of these enzymes are absent from the transgene. Digested genomic DNA was then heat inactivated for 20 min at 65ºC. 2 µL of digested genomic DNA was circularised overnight at 16ºC using T4 DNA Ligase (NEB). Subsequently, inverse and nested PCR were performed using high-fidelity HS VeriFi DNA Polymerase (PCRBIO) and two pairs of *Tol2* primers (7) as listed in Table S2. Amplicons were then visualised in 1% agarose by gel electrophoresis, and then gel extracted using purification kit before Sanger sequencing (Genewiz). Sequence analysis was performed using BLASTN (NIH) and Zebrafish Assembly GRCz11 (ENSEMBL), which identified a single insertion locus, in intron 3 of an uncharacterised gene, *si:ch211-222f23.6* (ENSDARG00000042880), in the minus strand of chromosome 9 (Fig. S1A). The transgene is transcribed in the same direction as the endogenous gene. Although *kg333Tg* homozygotes are viable and fertile, to minimise potential effects arising from modification of the locus, homozygotes were not used during the study.

Pharmacological Treatments

Tricaine (Sigma-Aldrich) was prepared as described (1) and used at 1× (160 mg/L), unless otherwise specified. The following compounds were dissolved in DMSO: bafilomycin A1 (MP Biomedicals; Cayman) at 500 µM, bortezomib (ApexBio) and MG132 (Alfa Aesar) at 1 mM, Nobiletin (Cayman) at 5 µM (8), rapamycin (Enzo Life Sciences), SR8278 (Cayman) and SR9011 (Sigma-Aldrich) at 5 mM (9), Terfenadine (Sigma-Aldrich) at 10 mM, Torin1 (ApexBio) at 10 mM, Myomed-205 (10-12) at 25 mM, and cycloheximide (Sigma-Aldrich) at 100 mg/mL; while MRT68921 (13) (Sigma-Aldrich) was dissolved in MilliQ water at 10 mM. All compounds were stored at -20ºC, and diluted to 200 µg/mL (cycloheximide), 250 nM (bafilomycin A1), 1 µM

(bortezomib, MRT68921, and Torin1), 3 μM (Terfenadine), 5 μM (SR8278, and SR9011), 10 μM (MG132, Nobiletin, and rapamycin), and 100 μM (Myomed-205) alone or in combination with other compounds in fish water (with or without tricaine) when used. Control groups were treated with 0-0.4% vehicle alone. None of the drugs appeared to be grossly toxic to the animals after 12-24 h treatment between 3 and 4 dpf.

Protein detection

Total protein was extracted from a pool of 10 larvae per biological replicate (either live or snap-frozen in liquid nitrogen) via sonication on ice in solution containing Tissue Extraction Reagent I (Invitrogen), cOmplete™ Protease Inhibitor Cocktail (Roche), and phenylmethylsulfonyl fluoride (PMSF). For proteasomal flux assay (Figs 2D, 3B), prior treatment with bortezomib allows the accumulation of ubiquitinated proteins, which would otherwise be degraded and recycled, and thus enhances their detection (14, 15). Addition of N-ethylmaleimide (NEM) into the solution mixtures at a final concentration of 25 mM (Figs 2D, 3B) further preserves and enhances the detection of ubiquitinated proteins by inactivating de-ubiquitinase enzymes during extraction (16). Protein extracts were then pelleted by centrifugation at 4ºC, after which the supernatant was mixed with Laemmli Sample Buffer (Bio-rad) and 2-mercaptoethanol and heated at 95ºC for 5 min, before subjecting to SDS-PAGE. Protein extract equivalent to 1-2 larvae was loaded per lane. SDS-PAGE was performed in Tris-glycine SDS running buffer using Mini-PROTEAN® TGX Precast Gels (Bio-rad); separated proteins were then wet-transferred onto nitrocellulose or PVDF membranes in Tris-glycine transfer buffer containing 10% methanol. Membranes were then incubated in Ponceau S solution briefly and imaged to quantify total protein. Ponceau S-labelled membranes were then washed in Tris-buffered saline containing 0.1% TWEEN 20 (TBST) before blocking in either 5% non-fat dry milk or 5% BSA in TBST for 30-60 min at room temperature. After blocking, membranes were sequentially incubated in primary (at 1:1000) and secondary (at 1:5000) antibodies in blocking buffer at 4ºC overnight and at room temperature for 1-2 h, respectively. Primary and secondary antibodies used were listed in Table S2. Amersham ECL Prime Western blotting detection reagent was used for HRP detection (GE Healthcare). Membranes were washed in 3-5 changes of TBST in 5-10 min after each antibody. All signal detection was performed using ChemiDoc™ Imaging System (Bio-rad) and analysed on Image Lab™ Software (Bio-rad). All replicate blots are shown in Figures S14-S22.

Proteasome activity assay

Total larval extracts were collected from a pool of 5-10 larvae per biological replicate (either live or snap-frozen in liquid nitrogen) by sonication on ice in Tissue Extraction Reagent I (Invitrogen), cleared by centrifugation at 4ºC and supernatants stored at -20ºC. To assay for proteasome activity, Proteasome-Glo Chymotrypsin-Like Assay kit (Promega) was used according to manufacturer's instructions. Briefly, larval extracts were added into reagent mixtures in 1:1 ratio (equivalent to 0.25 fish) in a reaction volume of 100 μL, in 96-well tissue

culture treated plates, flat-bottom, white, sterile (Greiner Bio-One). For control experiments, small molecules were also spiked in the reagent mixtures at a final concentration as indicated (Fig. S8A). The plate was then inserted into a Mithras LB 940 plate reader (Berthold Technologies) and automatically shaken for 2 min as programmed by the MikroWin 2000 software. After mixing, the plate was allowed to react for 10 min before luminescence detection. Background luminescence was detected in wells containing the extraction buffer alone plus the reagent mixture, and subsequently subtracted from all samples. Data were normalised to control samples.

OPP decorporation assay

OPP assay (Kelu et al., 2020) was modified to develop the de-corporation assay (Crossland et al., 2017). Briefly, a pool of larvae was treated with 50 µM Click-iT® OPP Reagent (Invitrogen) for 2 h. Then, larvae were washed with 3 changes of fish water in ~5 min to remove OPP. These OPP-incorporated larvae were then divided into four groups, A-D. For group A, larvae were fixed immediately with 2% paraformaldehyde (PFA) in phosphate-buffered saline (PBS) at room temperature for 30 min (t=0). For group B, larvae were returned to fish water and then fixed at 2 h (t=2), 6 h (t=6), and 10 h (t=10) after the OPP incorporation. For group C and D, larvae were incubated in either MG132 or bafilomycin A1 in fish water, and then fixed at t=10. To visualise OPP signals, fixed larvae were first bleached in 10% H_2O_2 and 5% formamide solution for 5-10 min to remove pigment. Bleached larvae were then washed with PBS containing 0.1% Triton X-100 (PBST) and subsequently labelled with Click-iT® Plus OPP Alexa Fluor™ 488 reaction cocktail (Invitrogen) for 1-2 h in dark in room temperature according to manufacturer's instructions. Labelled larvae were washed thoroughly with PBS, mounted and subjected to confocal imaging.

A negative control group of larvae were pretreated with the translation inhibitor cycloheximide (CHX) 1 h before OPP incorporation to reveal residual/unbound OPP (Fig. S5D). These CHX-treated larvae were fixed along with group A at t=0, and B at t=2, 6, and 10 as described above. The background OPP signals obtained from these negative control larvae were subsequently subtracted from the corresponding samples in group A and B to determine true OPP in-/de-corporation (Fig. S5E).

Whole-mount Immunostaining

Larvae were fixed with 2% PFA, washed with PBST, permeabilised with PBST containing 1% DMSO (PBDT) for an hour, blocked with PBDT containing 5% goat serum for 2 h at room temperature, then incubated in primary (A4.1025 at 1:10; 6xHis, cMyc, N1r1d, RpS6$^{Ser240/244}$-P, and Ulk^{Ser757}-P at 1:250; α-actinin, anti-GFP, and anti-mCherry/RFP at 1:500) and then secondary (at 1:1000) antibodies in blocking buffer containing 0.02% sodium azide at 4ºC for 2-3 overnights. Hoechst 33342 (at 1:5000-10000) was used as nuclear counterstain. Larvae

were washed thoroughly after each antibody incubation with PBST, and stored in PBS containing 0.02% sodium azide at 4ºC until confocal imaging.

Imaging

For wide-field imaging, samples were mounted in 0.8-1% low melting point agarose (LMPA) or 3% methylcellulose before imaging on a Leica MZ16F with iDS camera. For confocal imaging, samples were mounted in 0.8-1% LMPA and imaged on LSM 5 Exciter or LSM 880 microscopes (Carl Zeiss) equipped with 20x/1.0W objective. Samples are shown in lateral view (anterior to left, dorsal up) unless otherwise specified. For any live imaging, larvae were transiently anaesthetised with tricaine. For circadian muscle growth assays where individual larvae were scanned repeatedly, larvae were retrieved and washed immediately after each scan, and then separately housed in 24-well plates to recover and grow before the next scan. Medium in each well was changed every 12 h. To visualise cell membranes in live fish on an otherwise wildtype background, larvae were incubated in the dark in 5-10 µM BODIPY™ FL C5-Ceramide (Thermo Scientific; from 5 mM stock in DMSO) in fish water at 28°C overnight or 3 h at room temperature. Labelled larvae were washed briefly with fish water to remove excess dye before mounting and imaging. Within an experiment, all samples were imaged using identical settings.

Larval tail movement assay

We modified a previous protocol (17, 18), such that electrical instead of tactile stimuli were provided to trigger muscle contractions in zebrafish larvae. Larvae were first anesthetised and then mounted on a depression slide in 0.8-1% LMPA in a dorsal view (anterior to top and posterior to bottom). Once the agarose set, the trunk was freed from embedding by carefully removing any agarose below the level of the yolk/yolk extension transition using scalpel and forceps. A pair of platinum wires were then installed and positioned perpendicular to the anterior-posterior axis of the larvae ~1 mm apart; the set up was then flooded with fish water containing tricaine, and stabilised for 10 min before stimulation with three trains of electrical pulses (~0.5 s each, 4.5 s rest) using parameters previously described (19). Assays were performed at room temperature (~23ºC). Tail movements were recorded at 25 fps for 20 s under a Leica MZ16F with iDS camera.

Larval cardiac function assay

Larvae were removed from the incubator, mounted in medium containing either vehicle control or drug of interest, oriented in lateral view to clearly reveal the cardiac chambers, and equilibrated at room temperature (~23°C) for a minimum of 1 h prior to video recording of beating heart at 25 fps for 20 s (500 frames) under a Leica MZ16F with iDS camera. To suppress body movement, some larvae were mounted in 1.5% methylcellulose containing drug/vehicle and 0.5× tricaine (80 mg/L), a low-dose shown previously not to alter heart rate (20)). Alternatively, anaesthetized larvae constrained in 0.8% LMPA were perfused with fresh

fish water with drug/vehicle (but lacking tricaine). Heart rates under the same drug or vehicle treatment with either mounting method were indistinguishable (p=0.265).

Graphical illustrations and schematics

Graphs were plotted using GraphPad Prism and then modified in CorelDraw X7. Schematics were created either using Biorender or CorelDraw X7.

Quantification and statistical analysis

Larval myotome measurement

XY and *YZ* images were collected on somite 17 near the anal vent and subsequently processed and measured using ZEN (Carl Zeiss) or Fiji (NIH) software as previously described (2, 19).

Larval muscle tail movement measurement

Tail movements of head-embedded larvae were tracked using ZebraZoom (21). Briefly, positions/coordinates of the base and tip of each tail were manually assigned, and then unbiased tail tracking was performed on the first 450 frames (~18 s) of each video using the built-in module 'Head-embedded fish tail tracking'. Behaviour analysis was conducted on the tracked trajectories of tail movements to obtain 'maxTailAngleAmplitude' measurement. 'Tail Angle' is defined as the angle between the anterior-posterior axis of the fish and the axis formed by the base and the tip of the tail (see Fig. 1C of (21)). Thus, maxTailAngleAmplitude' represents the maximum absolute value of this angle over the entire bout (~18 s). Trajectories of the tail movements were visualised by superimposing all video frames using 'Minimum intensity projection' in Fiji (NIH).

Heart rate measurement

Rhythmic fluctuations in pixel intensity caused primarily by expulsion of red blood cells, were used to measure heart rate. Intensity profiles were extracted in Fiji (NIH) by drawing a line region of interest (ROI) across the cardiac chambers to generate an intensity profile over time (Fiji 'Z'-axis). The pracma package in *R* was used to detect local maxima (peaks) in the intensity signal using the findpeaks() function, with the minimum peak height set to the 50th percentile of the signal. The number of detected peaks over the total video duration (20 s) was used to calculate the heart rate (beats per minute, bpm) using the formula:

$$\text{Heart Rate (bpm)} = \left(\frac{\text{Number of Peaks}}{\text{Video Duration (s)}}\right) \times 60$$

Inter-beat intervals were calculated as the time difference between consecutive peaks.

Puncta counting (EGFP-LC3 and mCherry-p62) and area fraction (Ulk1^{Ser757}-P) measurement

Confocal *Z*-stacks were collected on somite 17-18 near the anal vent. All confocal stacks were rotated in Fiji (NIH) using 'Volume Viewer' and 'TransformJ' plugins to allow the selection of a

flat and equidistant *XY* plane for fair analysis. For confocal stacks obtained from live samples, the *XY* plane was selected from ~30 µm below the most superficial epithelial layer; whereas for fixed samples, the *XY* plane was instead selected from ~20 µm below surface as tissue shrinkage happened after fixation. All measurements were performed on a defined area based on the boundary of somite 17, which was manually defined using 'Polygon selections'. Thresholding was then applied to this defined area on the fluorescent channel that corresponds to the LC3/p62/Ulk1^{Ser757}-P labelling. Subsequently, an unbiased and automated analysis was performed on the thresholded LC3/p62/Ulk1^{Ser757}-P signals using 'Analyse Particles'. Particles on edges were excluded. For the analysis of mCherry-p62 puncta, a size exclusion of >10 µm^2 was applied to exclude most, if not all, nuclear signals (the size of myonuclei was empirically determined to be ~20 µm^2). Data were normalised to control samples.

Fluorescent signal (OPP and RpS6$^{Ser240/244}$-P) quantification

Confocal *Z*-stacks were collected and rotated, and analysed using Fiji (NIH) as described above. Briefly, boundary of somite 17 was defined and then 'Mean gray value' of the fluorescent signals within the defined area measured. For the analysis of OPP signals, the average 'Mean gray value' of the CHX-treated samples was subtracted from each experimental sample before log2 transformation. For the analysis of RpS6$^{Ser240/244}$-P signals, somite boundary was specifically defined to exclude the myosepta, which contain non-specific signals (see dashed lines and asterisks in Figs 6F, 7B, S10B). To obtain relative quantification, signals from each sample were divided by the mean signals of the control samples, which was set as 1.

Single myofibre measurements

To measure myofibre cross-sectional area (CSA), transverse confocal images were collected on S15-S18. The boundary of each selected myofibre, visualised by BODIPY-Ceramide staining, was manually outlined using 'Polygon selections' in Fiji (NIH). As a control, measurement was also performed on ≥2 (usually all) mCherry-negative myofibres immediately next to each mCherry-positive myofibre. Small, superficial slow fibres were excluded from the analysis.

To measure the fluorescent signals of RpS6$^{Ser240/244}$-P, the boundary of each mCherry-positive myofibre was first defined using 'Wand (tracing) tool' in Fiji (NIH) by thresholding the mCherry channel and the 'Mean gray value' of RpS6$^{Ser240/244}$-P signal measured within the defined area. As the boundary of individual mCherry-negative myofibres could not be traced, RpS6$^{Ser240/244}$-P signal was also measured in an adjacent mCherry-negative myofibre randomly-oriented region using the same area selection and the signal ratio of mCherry-positive to mCherry-negative area calculated.

To measure the area fraction of Ulk1^{Ser757}-P, the boundary of each mCherry-positive myofibre was defined, and then thresholding applied to the Ulk1^{Ser757}-P labelling within the defined

myofibre area. Subsequently, ‘Analyse Particles’ was performed on the thresholded signals and control measurements and ratios calculated as described above.

Western blot quantification

Western blot quantification was performed using Image Lab™ Software (Bio-rad). Lanes were semi-automatically defined using the ‘Lane and Bands’ tool, with individual adjustments to ensure optimal alignment. Bands within each lane were automatically detected using default settings. Signal intensities for bands/ranges and background signals were extracted from the ‘Lane Statistics’ in the ‘Analysis Table’. The ‘Adjusted Band Volume’ (for bands) and ‘Adjusted Lane Volume’ (for entire lane regions) represent the background-subtracted values used in subsequent normalisations. First, normalisation was performed against the total Ponceau S signals (22) within each corresponding lane. Next, for relative quantification, control sample values from each replicate blot were averaged, and this mean value was used as the denominator for each individual Ponceau-normalised sample. As a result, the mean normalised signal for control samples is set to 1 in each experimental series, while individual data points reflect the full variance among the Ponceau-normalised samples.

For the quantification of ubiquitinated proteins, detection was analysed across molecular weight ranges of 25 kDa and above, as the lower M_r membrane was probed for Histone H3. Notably, variability in the detection of lower molecular weight proteins (particularly in the 35-75 kDa range) was observed. This variability appeared to correlate with differences in the lot of Tissue Extraction Reagent I (Lot #1750253A versus Lot #308429000; #FNN0071, Invitrogen) used across different replicates (circle, square, triangle, diamond, and hexagon in Figures S14B and S15B, processed in chronological order). Despite these variations, the overall results remained consistent across experiments, with similar detection patterns observed and the smaller M_r bands consistently detected in the most recent replicates (diamond replicate in Figures S14B and hexagon replicate in Figure S15B).

qPCR relative quantification

ΔCt was calculated by subtracting the Ct value of the stable housekeeping gene *eef1a1l1* (2) from the target gene. ΔΔCt of each target gene was then calculated by subtracting the mean ΔCt of samples, and relative gene expression calculated as $2^{-\Delta\Delta Ct}$ (23), followed by normalisation to control samples, the mean mRNA level of which is set as 1.

Circadian detection and estimation

For detection of rhythmic components in qPCR data sets, JTK_Cycle algorithm (24) was applied using the MetaCycle package (25). For estimation of circadian parameters CircaCompare algorithm and package (26) were applied. The execution of both packages utilised the *R* programming language and RStudio.

Find Individual Motif Occurrences (FIMO) analysis

FIMO, part of the MEME Suite (https://meme-suite.org/), was used to identify RORE and RevDR2 motifs in the regulatory region of selected genes. The motif scan was focused on the genomic sequence between 5000 bp upstream of the transcription start site and the end of the intron immediately downstream of the first coding exon. The sequences of *tsc1a* (ENSDARG00000026048), *tsc1b* (ENSDARG00000057918), *tsc2* (ENSDARG00000103125), *murf1/trim63a* (ENSDARG00000028027), and *ulk1a* (ENSDARG00000062518) were downloaded from ENSEMBL Zebrafish (GRCz11) and position weight matrices corresponding to RORE (classic; WAWNTAGGTCA), RORE (variant; CWRGGNCA), and RevDR2 (AGGTCANNAGGTCA) motifs were created. The sequences and position weight matrices were uploaded to FIMO. Default settings were used: the default background model was applied, both strands of the DNA were scanned with a *p*-value cut-off of 1E-04. Resulting files were downloaded and analysed using Microsoft Excel and SnapGene.

Statistical analysis

Sample size was estimated from variance observed in previous work (2). No experiments were randomised and the investigator was not blinded to any dataset. Average values were calculated from 'n', where 'n' represents individual fish or biological replicates, as indicated in each figure or legend. All data were shown with bars representing mean ± standard error of the mean (SEM). As absolute staining intensity varies with each lay/batch/date of detection, independent biological replicates are the batch/date of extraction/blot (for Westerns) or mRNA extraction (for RT-qPCR), or individual fish (for wholemount stains) with batch ('Lay') used as a factor in the ANOVA. For myotome volume or genetic markers, independent biological replicates are defined as individual fish, but experiments were performed at least three times on fish from independent lays, as specified in figures; symbol shapes distinguish individuals from separate lays. According to previous work (2), measurements of myotome volume are normally distributed and homoscedastic. Hence, two-tailed unpaired and paired sample parametric tests (student *t*-test and ANOVA) were used. Bonferroni's post-hoc tests were performed following ANOVA with any two ('Time'/ 'Treatment'/ 'Genotype'/ 'Lay') or three ('Time', 'Treatment', and 'Genotype') factors. In common with almost all other Western/qPCR analyses published, our Western blot and qPCR experiments were analysed by parametric statistics although, because the total number of biological replicates was only 3-6, we cannot confirm normality and homoscedasticity with confidence. Numerical data were recorded in Microsoft Excel. All statistical tests and plots were performed using GraphPad Prism. *P*-values for rejection of the null hypothesis of no difference between groups are indicated above lines ending at the columns compared (for example, 1E-06 abbreviates 1×10^{-6}), and $p<0.05$ was considered significant. To further assess False Positive Risk (FPR), the Sellke, Bayarri, and Berger method (27) was applied using the *R* package 'pcal' (Table S3). For ease of reference for readers, FPR are also indicated in place of *p*-values on a duplicate set of Figures 1-6 and Supplementary Figures S1-S12 are presented as Figures S23-S40. We note that many muscle

size comparisons in which $p<0.05$ but FPR>0.05 are replicated elsewhere within the dataset or in our previous study (2), thereby reducing overall FPR.

Data Availability

Raw and derived/processed numerical data underpinning to each Figure panel are available at https://doi.org/10.18742/28816151.

Figure S1

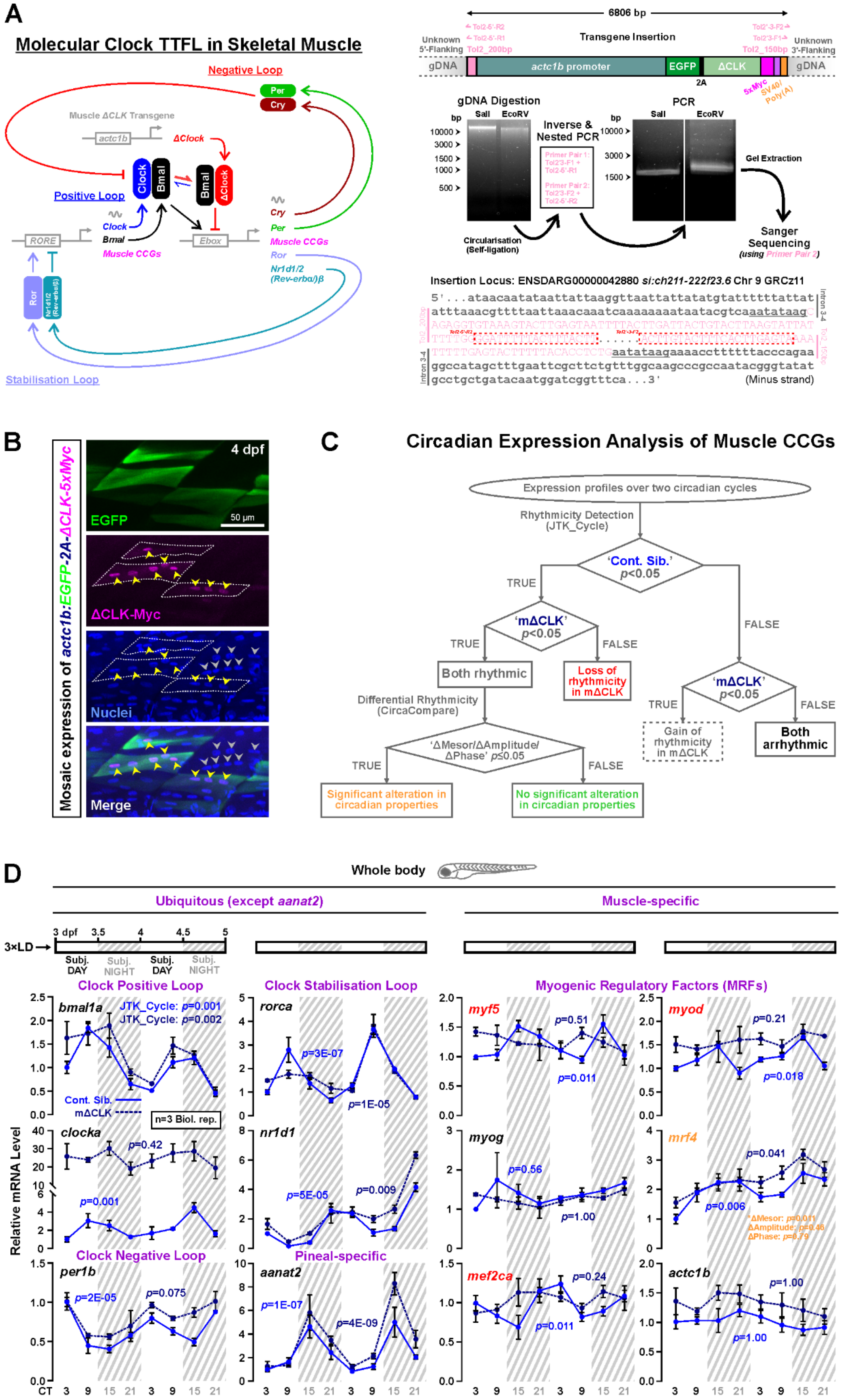

A
Molecular Clock TTFL in Skeletal Muscle
Negative Loop
Positive Loop
Stabilisation Loop
Muscle ΔCLK Transgene
Muscle CCGs
6806 bp
Transgene Insertion
gDNA Digestion
PCR
Inverse & Nested PCR
Gel Extraction
Sanger Sequencing
Insertion Locus: ENSDARG00000042880 si:ch211-222f23.6 Chr 9 GRCz11
(Minus strand)
B
Mosaic expression of actc1b:EGFP-2A-ΔCLK-5xMyc
4 dpf
EGFP
ΔCLK-Myc
Nuclei
Merge
C
Circadian Expression Analysis of Muscle CCGs
Expression profiles over two circadian cycles
Rhythmicity Detection (JTK_Cycle)
'Cont. Sib.' p<0.05
'mΔCLK' p<0.05
Both rhythmic
Loss of rhythmicity in mΔCLK
Gain of rhythmicity in mΔCLK
Both arrhythmic
Differential Rhythmicity (CircaCompare)
'ΔMesor/ΔAmplitude/ΔPhase' p≤0.05
Significant alteration in circadian properties
No significant alteration in circadian properties
D
Whole body
Ubiquitous (except aanat2)
Muscle-specific
Clock Positive Loop
Clock Stabilisation Loop
Myogenic Regulatory Factors (MRFs)
Clock Negative Loop
Pineal-specific
Relative mRNA Level

Figure S1. Muscle clock inhibition does not affect circadian rhythms in non-muscle tissues.

(A) Function of mΔCLK (left) and mapping of the mΔCLK transgene insertion in *kg333Tg* (right). The genomic sequence shows the insertion locus in the intron 3 of *si:ch211-222f23.6* gene (ENSDARG00000042880) in chromosome 9, one member of a teleost-specific multigene family with predicted similarity to nectin-like cell adhesion molecules that is expressed in a non-spatially restricted manner in zebrafish embryos and larvae. TTFL is transcription-translation feedback loop.

(B) Confocal stacks showing the immunodetection of ΔCLK-Myc in the myonuclei of EGFP (green labelling)-positive myofibres in 4 dpf larvae that were injected with *actc1b:EGFP-2A-ΔCLK-5xMyc* plasmid (see Fig. 1A) at one-cell stage. Yellow and grey arrowheads indicate Hoechst 33342 myonuclei in EGFP-positive and -negative myofibres, respectively.

(C) Flow chart of circadian expression analysis. Outcomes colour-coded for ease of reference in Figs 2B, 4B, S1D, S2B,C, S7A,B, and S12B,C.

(D) Circadian expression of core clock genes (left columns) and muscle-specific transcripts (right columns) in whole body of control and mΔCLK$^{kg333Tg/+}$ larvae. RNA collection was performed under free-run as shown in schematics, subjected to RT-qPCR, and analysed as in **C**. CT is circadian time.

Figure S2

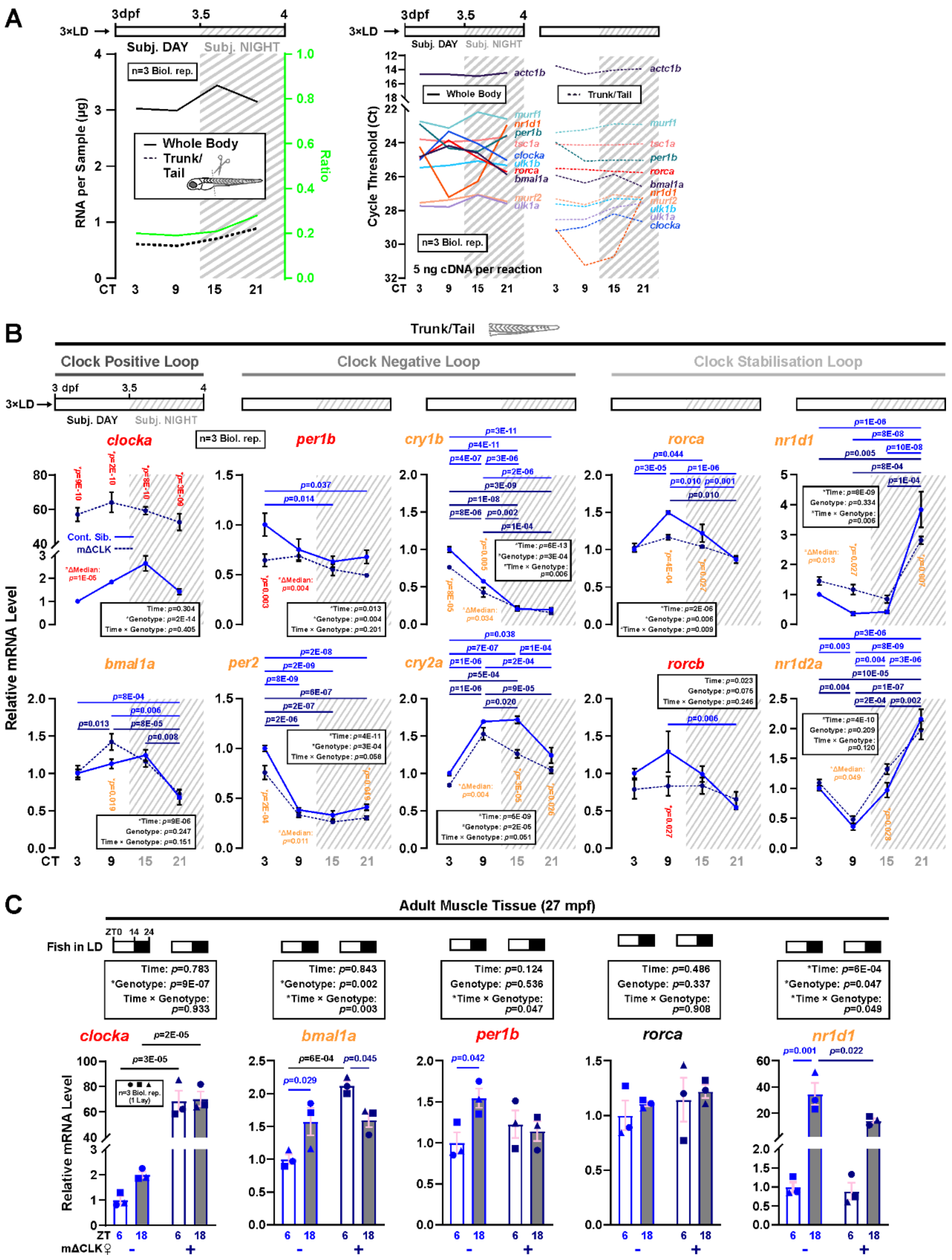

Figure S2. Muscle clock inhibition disrupts circadian rhythms in muscle tissue. **(A)** Comparison of abundance of RNA (left) and individual transcripts (right) in whole wild type larvae or trunk/tail under free run between 3 and 4 dpf. Triplicate biological (RT) and technical (qPCR) replicates; error bars omitted for clarity. Equal masses of total RNA and cDNA were added to each RT and qPCR reaction. Note the lower proportion of core clock gene transcripts

but raised proportion of some muscle transcripts in the trunk/tail compared to whole body, matching *in situ* hybridization results (28).

(B) Changes in circadian core clock gene mRNAs in trunk/tail of control and mΔCLK$^{kg333Tg/+}$ larvae. Larvae were decapitated every 6 h under free-run between 3 and 4 dpf and assayed by RT-qPCR. Statistics show two-way ANOVA (Time/Genotype) with Bonferroni's post hoc test.

(C) Diurnal expression of core clock genes in muscle tissue of control and mΔCLK at 27 mpf. Adult female fish were euthanised, and muscle tissues dissected and collected at ZT6 and 18 under LD. Statistics are two-way ANOVA (Time/Genotype) with Bonferroni's post hoc test. ZT, zeitgeber time.

Figure S3

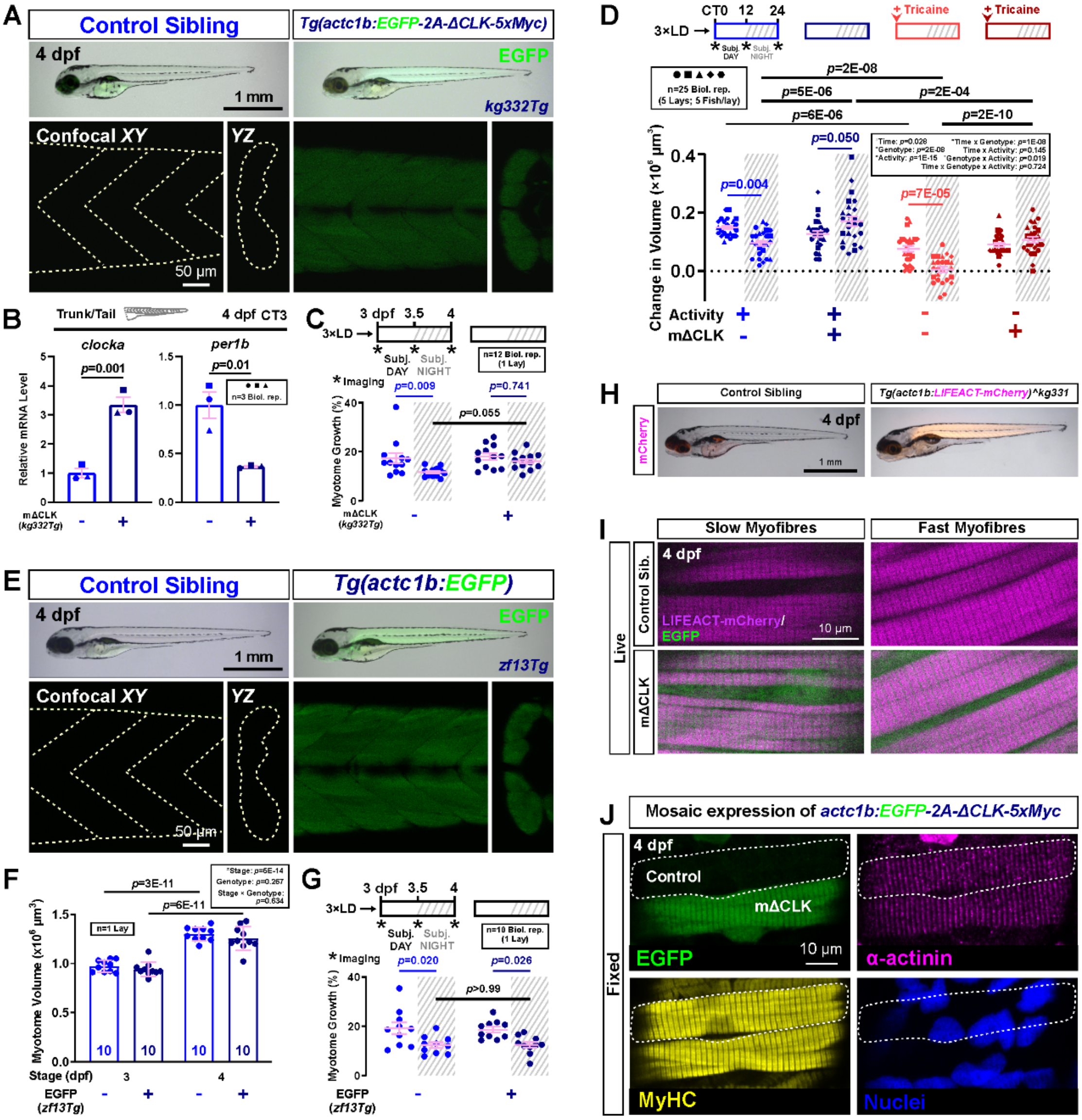

Figure S3. Muscle clock inhibition does not disrupt gross sarcomere structure.

(A) Brightfield/EGFP overlays (top) showing the gross morphology of control and mΔCLK$^{kg332Tg/+}$ siblings at 4 dpf. Single confocal XY (parasagittal) and YZ (transverse) slices of sibling fish at 4 dpf showing EGFP (bottom) in mΔCLK trunk musculature.

(B) Circadian expression of core clock genes in trunk/tail of control and mΔCLK$^{kg332Tg/+}$ siblings. Larvae were decapitated at CT3 at 4 dpf under free-run. Statistics are paired *t*-test. The expression of *Δclk* is weaker in *kg332Tg* than *kg333Tg*, but *per1b* is still suppressed.

(C) Circadian muscle growth measured with *actc1b:mCherryCAAX* in the presence or absence of ΔCLK in the muscle. Muscle growth of control and mΔCLK$^{kg332Tg/+}$ larvae was tracked each 12 h under free-run between 3 and 4 dpf. Statistics are two-way ANOVA (Time/Genotype) with Bonferroni's post hoc test.

(D) Circadian muscle growth of myotome 17 from 3-4 dpf (absolute change) measured with *actc1b:mCherryCAAX* in control and mΔCLK siblings. Muscle growth of individual larvae (3xLD entrained) was tracked every 12 h (*) under free-run conditions (constant light) between 3 and 4 dpf as shown in schematics. Sibling control and mΔCLK transgenic were either unanesthetised (active) or anesthetised (inactive, arrowhead). CT is circadian time. CT0-12 and CT12-24 are subjective (subj.) day and night, respectively. Statistics are three-way ANOVA (Time/Treatment/Genotype) with Bonferroni's post hoc test.

(E) Brightfield/EGFP overlays (top) showing the gross morphology of control and muscle EGFP *zf13Tg* siblings at 4 dpf. Single confocal XY and YZ slices of mΔCLK fish at 4 dpf showing EGFP in trunk musculature, showing that high-level EGFP has no effect on growth.

(F,G) Myotome volume (**F**) and circadian muscle growth (**G**) measured between 3 and 4 dpf in control sibling and muscle EGFP *zf13Tg* larvae under free-run. Statistics are two-way ANOVA (Stage/Genotype) with Bonferroni's post hoc test.

(H) Brightfield/mCherry overlay images showing the gross morphology of control and *actc1b:LIFEACT-mCherry kg331Tg* siblings at 4 dpf.

(I) Confocal XY (parasagittal) slices showing similar myofibrillar actin structure visualised in live sibling *actc1b:LIFEACT-mCherry*$^{kg331Tg/+}$ and *actc1b:LIFEACT-mCherry*$^{kg331Tg/+}$*; mΔCLK*$^{kg333Tg/+}$ (EGFP) larvae in superficial slow and deep fast myofibres.

(J) Confocal stacks showing the indistinguishable sarcomeric structure in EGFP-positive or -negative (dotted line) myofibres in 4 dpf larva that was injected at one-cell stage with *actc1b:EGFP-2A-ΔCLK-5xMyc* plasmid (see Fig. 1A). Sarcomeres revealed by the immunodetection of α-actinin and myosin heavy chain (MyHC). Hoechst 33342 nuclear counterstain.

Figure S4

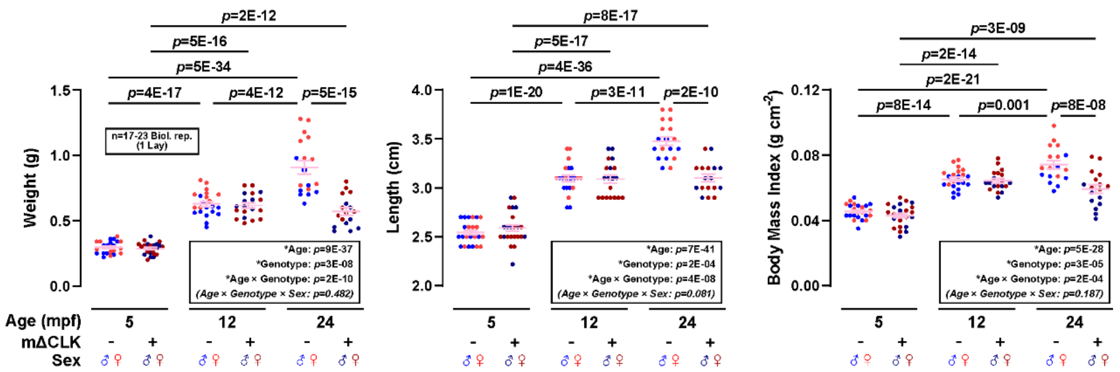

Figure S4. Muscle clock inhibition leads to premature sarcopenia.

Body weight, length, and body mass index (BMI) of control and mΔCLK siblings measured in male (blue symbols) and female (red symbols) at 5, 12, and 24 mpf. Fish were co-reared in the same tanks (e.g. density, feeding and housing conditions). Statistics are either two-way ANOVA (Age/Genotype) or three-way ANOVA (Age/Genotype/Sex) with Bonferroni's post hoc test.

Figure S5

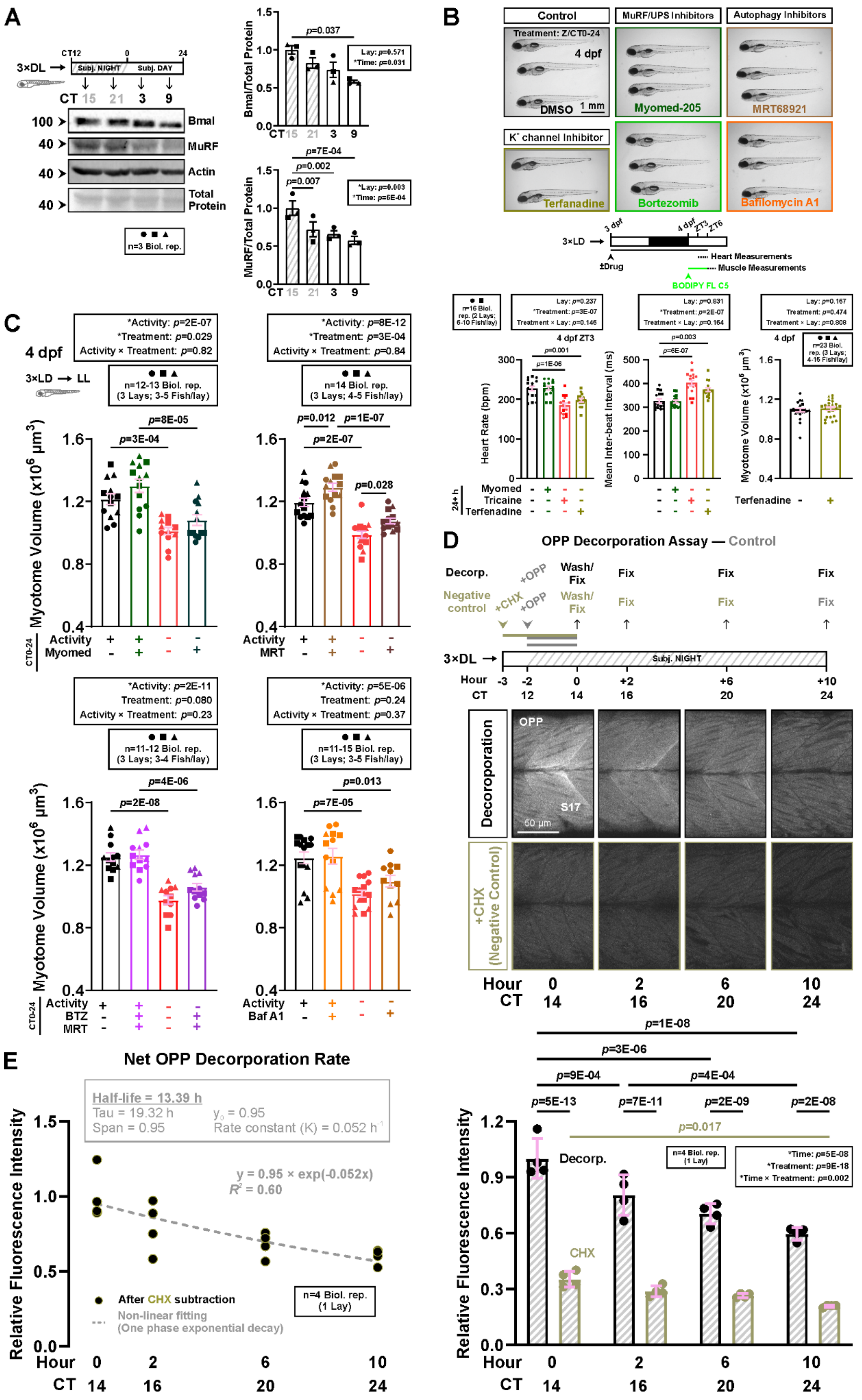

A
B
C
D
E
Bmal
MuRF
Actin
Total Protein
n=3 Biol. rep.
Control
MuRF/UPS Inhibitors
Autophagy Inhibitors
DMSO
Myomed-205
MRT68921
K+ channel Inhibitor
Terfenadine
Bortezomib
Bafilomycin A1
Heart Measurements
Muscle Measurements
BODIPY FL C5
4 dpf
Myotome Volume (x10^6 µm^3)
OPP Decorporation Assay — Control
Decoroporation
+CHX (Negative Control)
Net OPP Decorporation Rate
Half-life = 13.39 h
Tau = 19.32 h
Span = 0.95
y0 = 0.95
Rate constant (K) = 0.052 h-1
y = 0.95 × exp(-0.052x)
R2 = 0.60
After CHX subtraction
Non-linear fitting
(One phase exponential decay)
n=4 Biol. rep. (1 Lay)
Relative Fluorescence Intensity
Hour
CT

Figure S5. MuRF abundance and proteasomal clearance are higher at night.

(A) Circadian accumulation of MuRF and Bmal in zebrafish larvae under free-run after 3xDL entrainment from three replicate experiments. Statistics are two-way ANOVA (Lay/Time) with Bonferroni's post hoc test.

(B) Brightfield images taken at 4 dpf showing the gross morphology of zebrafish larvae treated between 3 and 4 dpf under free-run (CT0-24) with DMSO, Myomed, bortezomib, MRT6892, or bafilomycin A1, or under LD (ZT0-24) with Terfenadine. Cardiac output was altered by tricaine or terfenadine, but not by Myomed. Terfenadine, which reduces the heart rate, did not alter muscle size at 4 dpf following 30 h of exposure, indicating that optimal cardiac function is not essential for muscle growth at this developmental stage. Statistics are two-way ANOVA (Lay/Treatment) with Bonferroni's post hoc test.

(C) Myotome volume measured at 4 dpf in larvae that were treated under free-run between 3 and 4 dpf from CT0-24 without (active; black or light colour symbols) or with tricaine (inactive; red or dark colour symbols) and with either MuRF or autophagy inhibitors or both. Larvae were treated with 1) Myomed, 2) bafilomycin A1 (Baf A1), 3) MRT68921 (MRT), or 4) bortezomb (BTZ) in combination with MRT. Statistics are two-way ANOVA (Activity/Treatment) with Bonferroni's post hoc test.

(D,E) OPP decorporation assay schematic, assay (**D**) and rate calculation (**E**). DL-entrained larvae at 3 dpf were left untreated or treated with cycloheximide (CHX; negative control) between CT11 and 14 to stop protein translation, then exposed to OPP between CT12 and 14 to label nascent peptides. OPP-labelled peptides were then allowed to degrade overnight under free-run (decorporation). Fish were fixed at 0, 2, 6, and 10 h post OPP-washout. Single confocal XY slices (**D** top) showing OPP signals in somite 17 (S17) visualised by fluorescent Click-iT chemistry. Raw fluorescence signals normalised to Decorporation samples at 0 h (**D** bottom). Declining background signal was revealed by CHX treatment, perhaps due to residual unbound/unwashed OPP. Statistics are two-way ANOVA (Time/Treatment) with Bonferroni's post hoc test. Non-linear fits (**E**; dotted lines) measured the rate of decay of OPP fluorescence in the muscle. Background signals were subtracted from Decorporation samples (**D**) to estimate the true rate of peptide degradation.

Figure S6

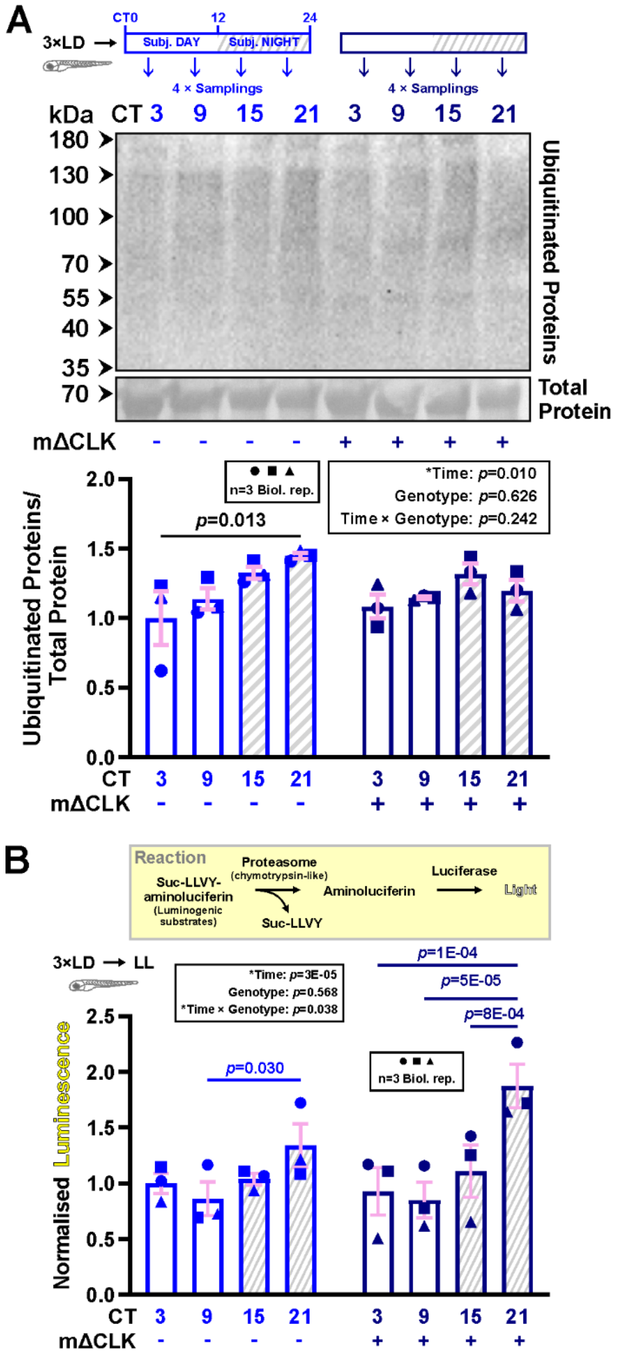

Figure S6. Ubiquitinated protein abundance and proteasome activity are higher at night. (A) Effect of muscle clock inhibition on circadian accumulation of ubiquitinated proteins in control and mΔCLK sibling larvae under free-run between 3 and 4 dpf. Ubiquitinated protein abundance was normalised to total protein. Statistics are two-way ANOVA (Time/Genotype) with Bonferroni's post hoc test. **(B)** Effect of muscle clock inhibition on circadian variations in proteasomal activity in control and mΔCLK sibling larvae under free-run between 3 and 4 dpf when subjected to chymotrypsin-like activity assay (see Reaction inset). Statistics are two-way ANOVA (Time/Genotype) with Bonferroni's post hoc test.

Figure S7

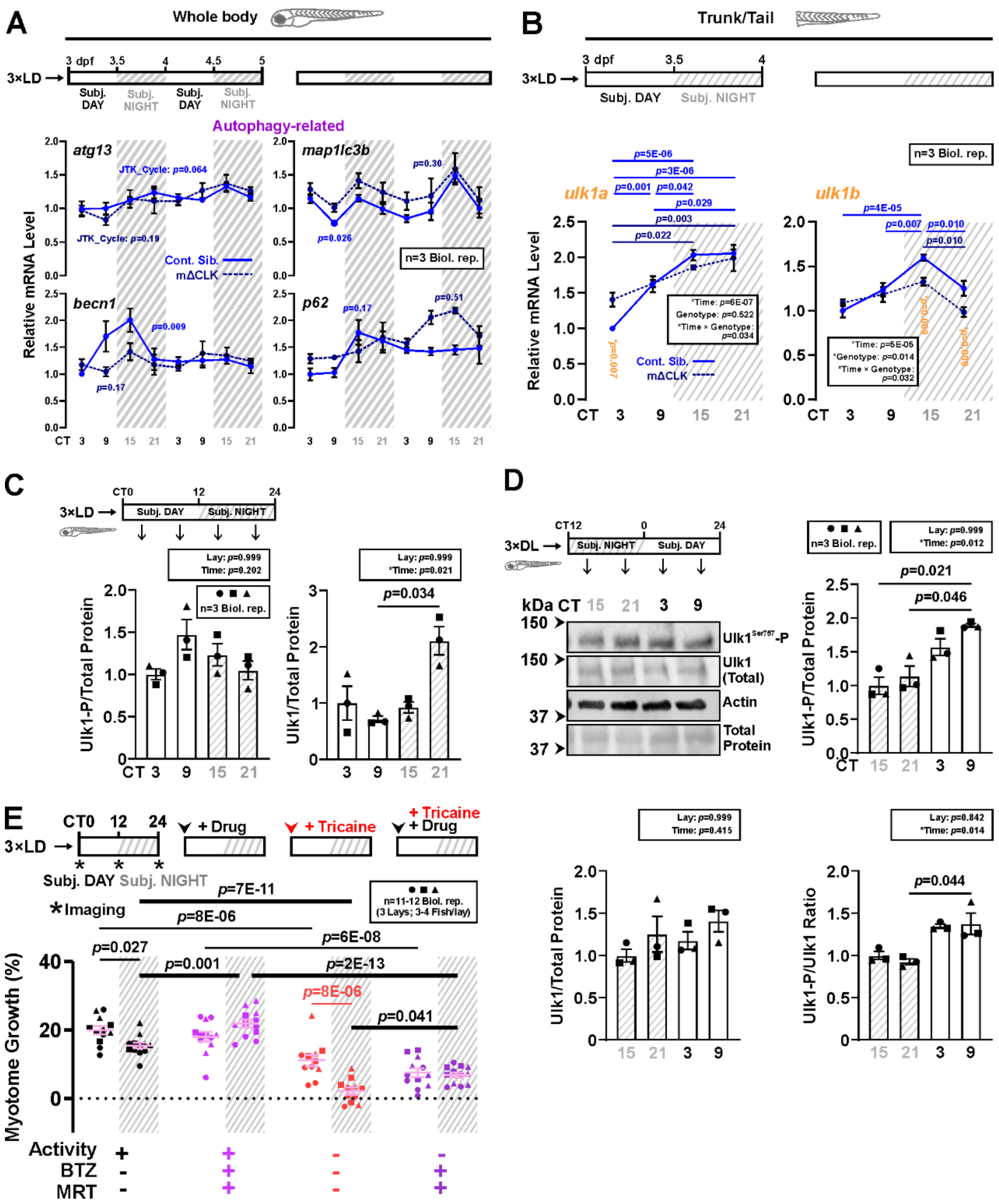

Figure S7. Circadian regulation of Ulk1 phosphorylation.

(A) Circadian mRNA levels of autophagy-related genes *atg13*, *becn1*, *map1lc3b*, and *sqstm1*/*p62* in 3xLD entrained control and mΔCLK siblings under free-run analysed by RT-qPCR (see Fig. S1C).

(B) Circadian mRNA levels of *ulk1a* and *ulk1b* in trunk/tail of control and mΔCLK siblings under free-run analysed by RT-qPCR (see Fig. S1C). Statistics are two-way ANOVA (Time/Genotype) with Bonferroni's post hoc test.

(C) Circadian oscillation of total Ulk1 and Ulk1^{Ser757} phosphorylation in zebrafish larvae under free-run after 3xLD entrainment from three replicate experiments. Statistics are two-way ANOVA (Lay/Time) with Bonferroni's post hoc test.
(D) Circadian oscillation of total Ulk1, Ulk1^{Ser757} phosphorylation, and their ratio, in zebrafish larvae under free-run after 3xDL entrainment from three replicate experiments. Statistics are two-way ANOVA (Lay/Time) with Bonferroni's post hoc test.
(E) Circadian myotome growth after autophagy and proteasome inhibition in entrained active or inactive larvae treated under free-run between 3 and 4 dpf with DMSO or bortezomib plus MRT68921. Statistics are two-way ANOVA (Time/Treatment) with Bonferroni's post hoc test.

Figure S8

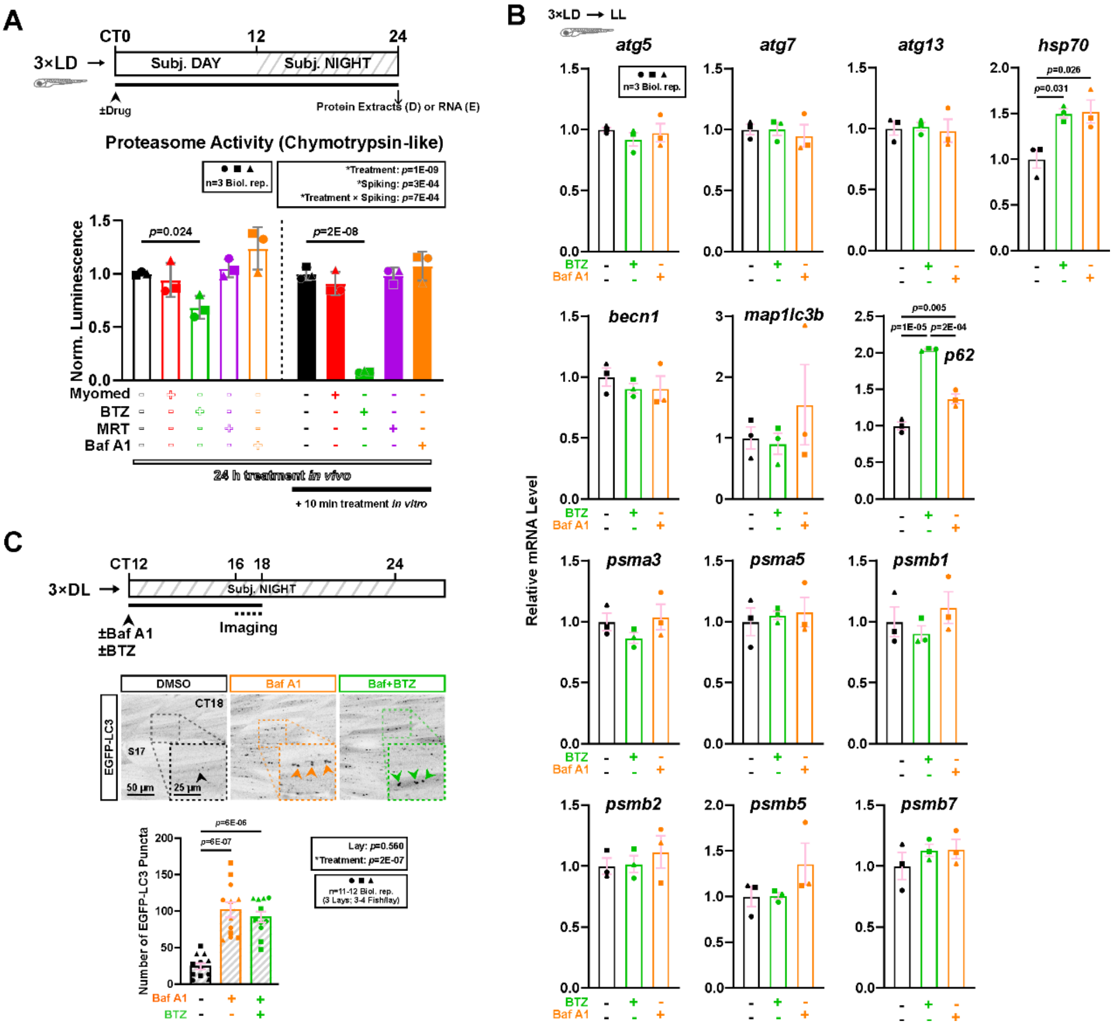

Figure S8. UPS and autophagy pathways are not interfered by inhibitors of each other. (A,B) Effects of MuRF, UPS, and autophagy inhibition on the chymotrypsin-like activity of proteasome (**A**) and mRNA levels (**B**). Entrained larvae under free-run were treated from CT0 with either DMSO, Myomed, bortezomib (BTZ), MRT68921 (MRT), or bafilomycin A1 (Baf A1) and then protein (**A**) or RNA (**B**) extracts collected at CT24. Extracts were subjected to proteasome assay (**A**, open bars) or RT-qPCR (**B**). As controls, after collection each protein extract was spiked with its drug (at the same final concentrations) 10 min before *in vitro* assay (**A**, filled bars). Statistics are two-way ANOVA (**A**; Treatment/Spiking) or one way ANOVA (**B**) with Bonferroni's post hoc test.

(C) Autophagic flux in muscle measured in *Tg(CMV:EGFP-map1lc3b)*^{zf155} entrained larvae under free-run between 3 and 4 dpf treated with DMSO, bafilomycin A1 (Baf A1), or Baf A1 and bortezemib (BTZ) at CT12 for 4-6 h. Confocal parasagittal images at CT16-18 revealed number of EGFP-LC3 puncta (arrowheads) in S17. Higher magnification shown in insets. Statistics are two-way ANOVA (Lay/Treatment) with Bonferroni's post hoc test.

Figure S9

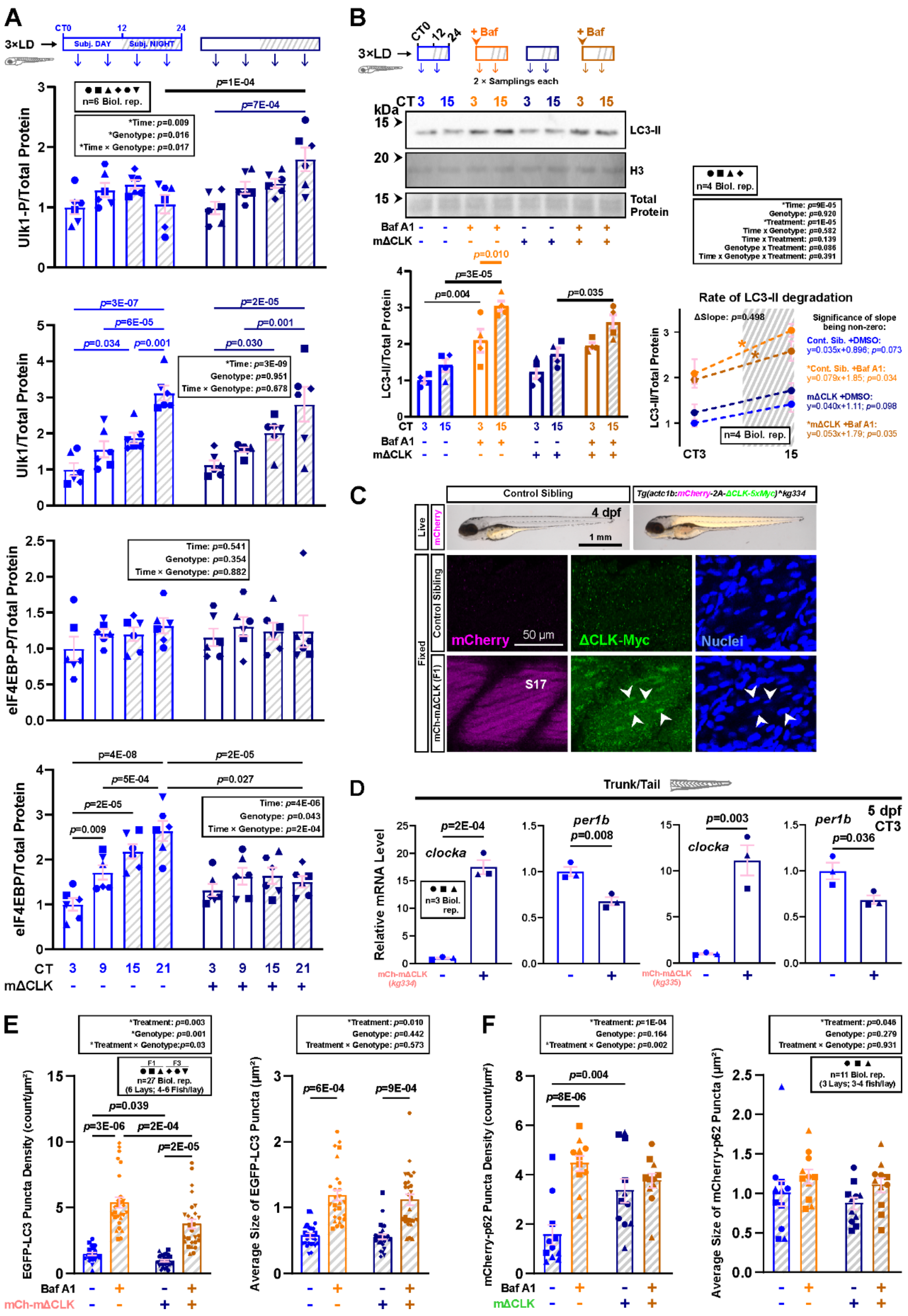
A
B
C
D
E
F
Ulk1-P/Total Protein
Ulk1/Total Protein
eIF4EBP-P/Total Protein
eIF4EBP/Total Protein
LC3-II/Total Protein
Rate of LC3-II degradation
Control Sibling
Tg(actc1b:mCherry-2A-ΔCLK-5xMyc)^kg334
mCherry
ΔCLK-Myc
Nuclei
Trunk/Tail
Relative mRNA Level
clocka
per1b
EGFP-LC3 Puncta Density (count/µm²)
Average Size of EGFP-LC3 Puncta (µm²)
mCherry-p62 Puncta Density (count/µm²)
Average Size of mCherry-p62 Puncta (µm²)

Figure S9. Muscle clock inhibition activates TORC1 signalling and reduces autophagic flux at night.

(A) Circadian oscillation of total Ulk1 and phosphorylated Ulk1^{Ser757}, and non-phosphorylated eIF4EBP and phosphorylated eIF4EBP$^{Thr37/46}$, in sibling control and mΔCLK entrained larvae under free-run between 3 and 4 dpf from six replicate experiments. Statistics are two-way ANOVA (Time/Genotype) with Bonferroni's post hoc test.

(B) Effects of muscle clock inhibition on circadian autophagic degradation of LC3-II in entrained control and mΔCLK sibling larvae under free-run between 3 and 4 dpf treated with either DMSO or bafilomycin A1 (Baf A1) at CT0, and protein analysed by Western at CT3 and CT15. Statistics are three-way ANOVA (Time/Genotype/Treatment) with Bonferroni's post hoc test. Linear regressions estimating the rate of change of LC3-II protein abundance between CT3 and CT15, in the presence or absence of Baf A1.

(C) Brightfield/mCherry overlay (top) of sibling control and mCherry-mΔCLK^{kg334Tg} at 4 dpf. After Myc-immunodetection and nuclear counterstain, single confocal parasagittal slices of mCherry-mΔCLK F1 fish S17 reveal mCherry in trunk musculature and ΔCLK-Myc in myonuclei (arrowheads).

(D) Circadian expression of core clock genes in trunk/tail of sibling control and mΔCLK *kg334Tg* or *kg335Tg*. Entrained 3xLD larvae were decapitated at CT3 at 5 dpf under free-run and analysed by RT-qPCR. Statistics are unpaired t-test.

(E) Nocturnal autophagic flux in muscle measured with *Tg(CMV:EGFP-map1lc3b)*^{zf155} in DL-entrained sibling control and mCherry-mΔCLK larvae under free-run treated at 3 dpf CT12 with either DMSO or bafilomycin A1 (Baf A1) and imaged between CT16 and 18. Confocal parasagittal images at CT16-18 visualised number and size of EGFP-LC3 puncta in S17. The number of EGFP-LC3 puncta was normalised to somite area (μm^2). Statistics are two-way ANOVA (Treatment/Genotype) with Bonferroni's post hoc test. F1/F3, filial generation.

(F) Nocturnal autophagic flux in muscle in DL-entrained sibling control and EGFP-mΔCLK larvae from embryos injected with *mCherry-p62* mRNA at one-cell stage and treated at 3 dpf CT12 under free-run with either DMSO or bafilomycin A1 (Baf A1) and imaged between CT16 and 18. Confocal parasagittal images at CT16-18 visualised number and size of mCherry-p62 puncta (arrowheads) in sarcoplasm in S17. The number of mCherry-p62 puncta was normalised to somite area (μm^2). Statistics are two-way ANOVA (Treatment/Genotype) with Bonferroni's post hoc test.

Figure S10

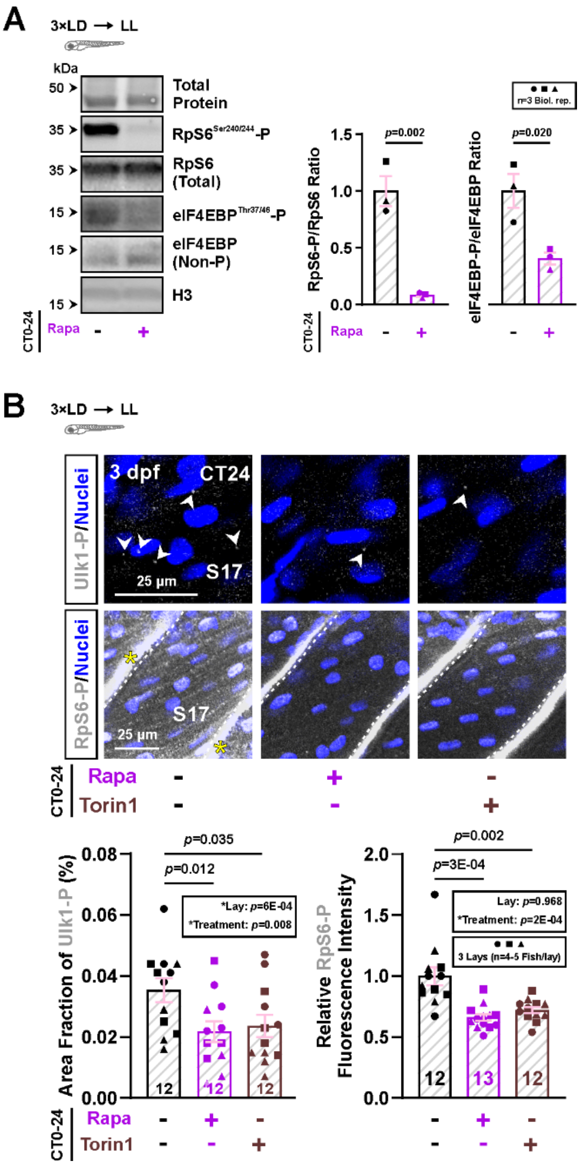

Figure S10. TORC1 signalling is inhibited by rapamycin or Torin1.

(A) TORC1 inhibition in 3xLD entrained larvae under free-run between 3 and 4 dpf treated from CT0 with either DMSO or rapamycin and protein collected at CT24. Statistics are unpaired t-test.

(B) Effects of TORC1 inhibition on phosphorylation of Ulk1^{Ser757} and RpS6$^{Ser240/244}$ in muscle. Entrained larvae under free-run between 3 and 4 dpf were treated from CT0 with either DMSO, rapamycin, or Torin1 and then fixed at CT24. After immunodetection and nuclear counterstain, phosphorylated Ulk1^{Ser757} puncta (arrowheads) and RpS6$^{Ser240/244}$ intensity were quantified in single confocal parasagittal slices in S17. Asterisks denote unspecific binding of the RpS6$^{Ser240/244}$-P antibody at the myosepta. Dashed lines define somite area for measurement by excluding the myosepta. Statistics are two-way ANOVA (Lay/Treatment) with Bonferroni's post hoc test.

Figure S11

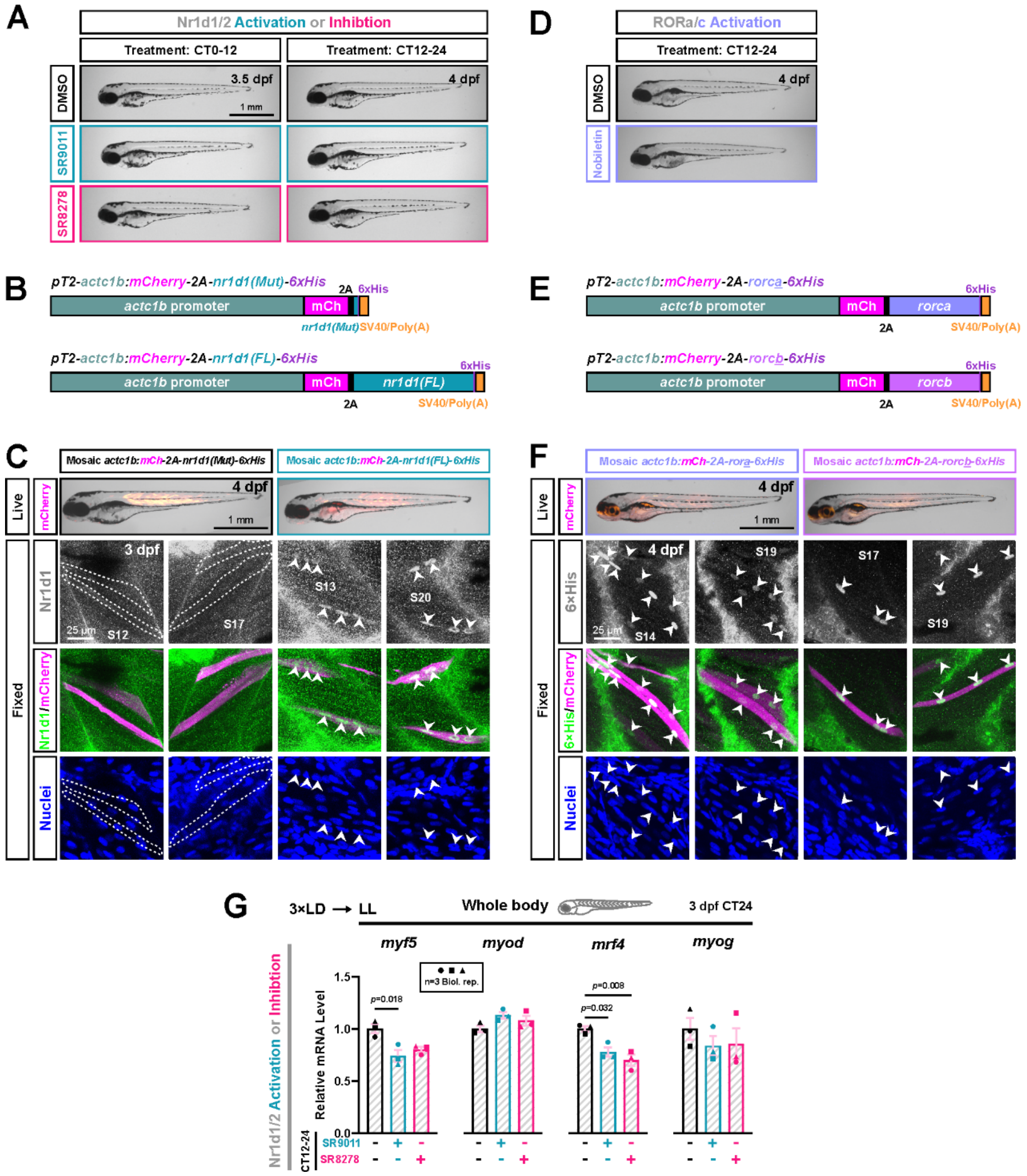

Figure S11. Nr1d1 and Rorc imbalance contributes to the phenotypes of mΔCLK fish.
(A) Brightfield images taken at 3.5 and 4 dpf showing the gross morphology of 3xLD entrained zebrafish larvae under free-run treated with DMSO, SR9011, or SR8278, either during CT0-12 or CT12-24, respectively.
(B) Design of muscle-specific mutant control (Mut) or full-length (FL) *nr1d1* expression plasmids.
(C) Brightfield/mCherry overlays (top) showing the gross morphology and mosaic expression at 4 dpf of control mutant (Mut) or full-length (FL) *nr1d1* construct in myofibres of injected larvae. Single confocal parasagittal slices showing Nr1d1 immunodetection and nuclear counterstain

in myonuclei (arrowheads) of *nr1d1(FL)*-injected larvae only, and mCherry immunodetection in sarcoplasm of all mosaically-labelled myofibres between S12-S20 at 3 dpf. Dots outline Mut myofibre boundaries.

(D) Brightfield images taken at 4 dpf showing the gross morphology of 3xLD entrained zebrafish larvae under free-run treated with DMSO or Nobiletin during CT12-24.

(E) Design of muscle-specific *rorca* and *rorcb* expression plasmids.

(F) Brightfield/mCherry overlays (top) showing the gross morphology and mosaic expression at 4 dpf of *rorca/b* plasmids in myofibres of injected larvae. Single confocal parasagittal slices showing His-tag immunodetection and nuclear counterstain in myonuclei (arrowheads) of *rorca*- and *rorcb*-injected larvae, and mCherry immunodetection in sarcoplasm of mosaically-labelled myofibres between S14-S19 at 4 dpf.

(G) Effects of Nr1d1/2 activation or inhibition on the expression of myogenic factors (*myf5*, *myod*, *mrf4*, and *myog*). Larvae under free-run from 3 dpf were treated from CT12 with either DMSO, SR9011 or SR8278 and RNA collected at CT24. Statistics are one-way ANOVA with Bonferroni's post hoc test.

Figure S12

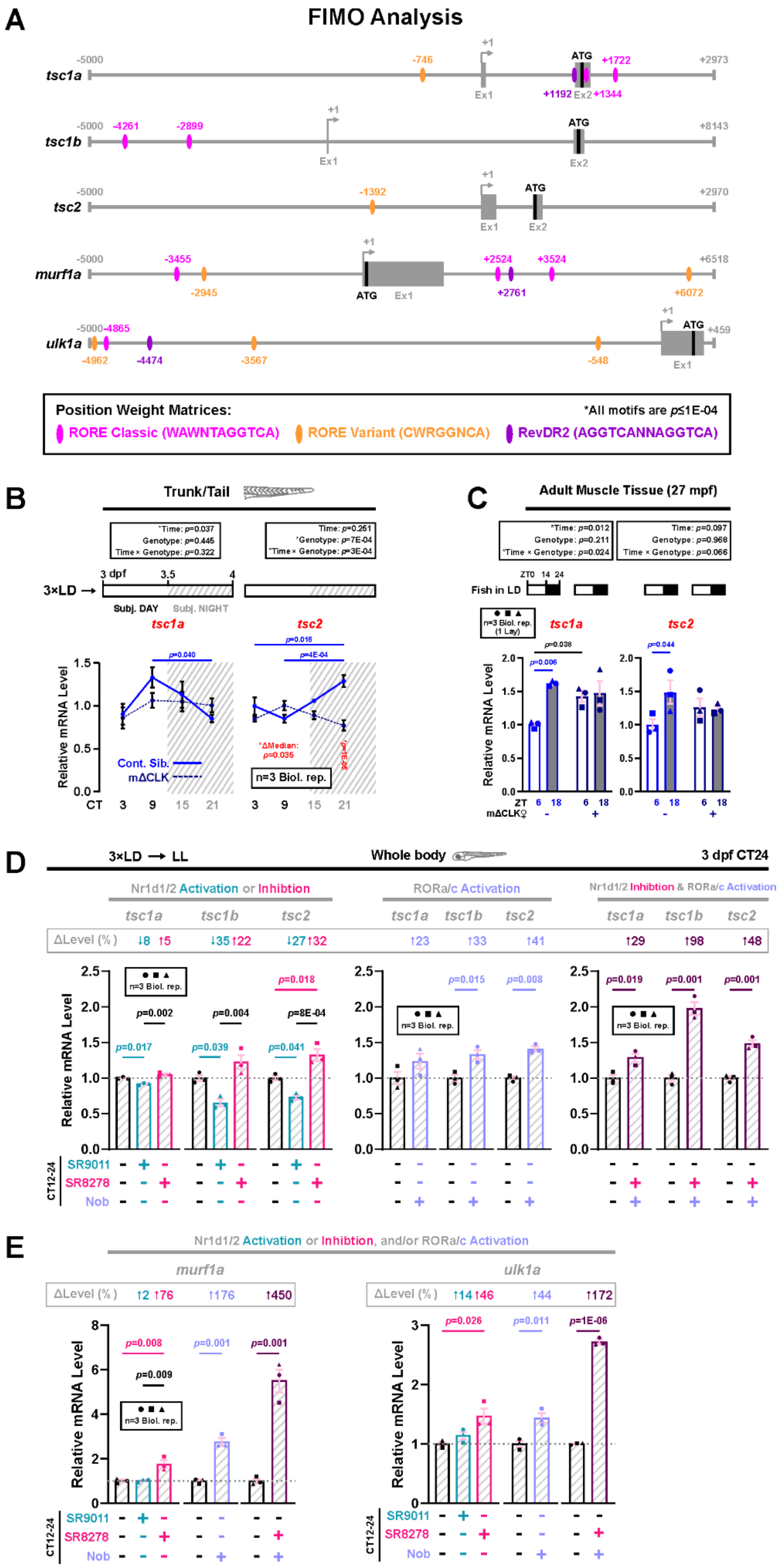

A
FIMO Analysis
tsc1a
tsc1b
tsc2
murf1a
ulk1a
Position Weight Matrices:
*All motifs are p≤1E-04
RORE Classic (WAWNTAGGTCA)
RORE Variant (CWRGGNCA)
RevDR2 (AGGTCANNAGGTCA)
B
Trunk/Tail
3×LD
Subj. DAY
Subj. NIGHT
Relative mRNA Level
Cont. Sib.
mΔCLK
n=3 Biol. rep.
C
Adult Muscle Tissue (27 mpf)
Fish in LD
D
3×LD → LL
Whole body
3 dpf CT24
Nr1d1/2 Activation or Inhibtion
RORa/c Activation
Nr1d1/2 Inhibtion & RORa/c Activation
ΔLevel (%)
SR9011
SR8278
Nob
E
Nr1d1/2 Activation or Inhibtion, and/or RORa/c Activation

Figure S12. *Tsc1/2* gene expression is regulated by the muscle peripheral clock.

(A) FIMO analysis for putative RORE and RevDR2 motifs identified (with a significance threshold of $p \leq 1E\text{-}04$) in the regulatory sequences of *tsc1a*, *tsc1b*, *tsc2*, *ulk1a*, and *murf1*. Arrows indicate the position of transcription start site (+1), whereas ATG is the position of the translation start site.

(B) Circadian expression profiles of *tsc1a* and *tsc2* in the trunk/tail of control siblings and mΔCLK siblings. Larvae were decapitated every 6 h under free-run between 3 and 4 dpf, and trunk/tail subjected to RT-qPCR (see Fig. S1C). Statistics are two-way ANOVA (Time/Genotype) with Bonferroni's post hoc test.

(C) Diurnal expression of Tsc transcripts in muscle tissue of control siblings and mΔCLK at 27 mpf. Adult female fish were euthanised at ZT6 and 18 under LD, muscle tissues dissected and subjected to RT-qPCR. Statistics are two-way ANOVA (Time/Genotype) with Bonferroni's post hoc test.

(D) Effect of Nr1d1/2 activation or inhibition, Rora/c activation, or both, on mRNA level of Tsc transcripts. At 3 dpf, entrained larvae under free-run were treated from CT12 with either DMSO, SR9011 SR8278, or Nobiletin and RNA collected at CT24 analysed by RT-qPCR. ΔLevel indicates % change from respective control. Statistics are one-way ANOVA with Bonferroni's post hoc test or unpaired *t*-tests.

(E) Effect of Nr1d1/2 inhibition, Rora/c activation or both on mRNA level of *murf1* and *ulk1a* transcripts. At 3 dpf, entrained larvae under free-run were treated from CT12 with either DMSO, SR9011 SR8278, or Nobiletin and RNA collected at CT24 analysed by RT-qPCR. ΔLevel (%) indicates change from respective control. Statistics are one-way ANOVA with Bonferroni's post hoc test or unpaired *t*-tests.

Figure S13

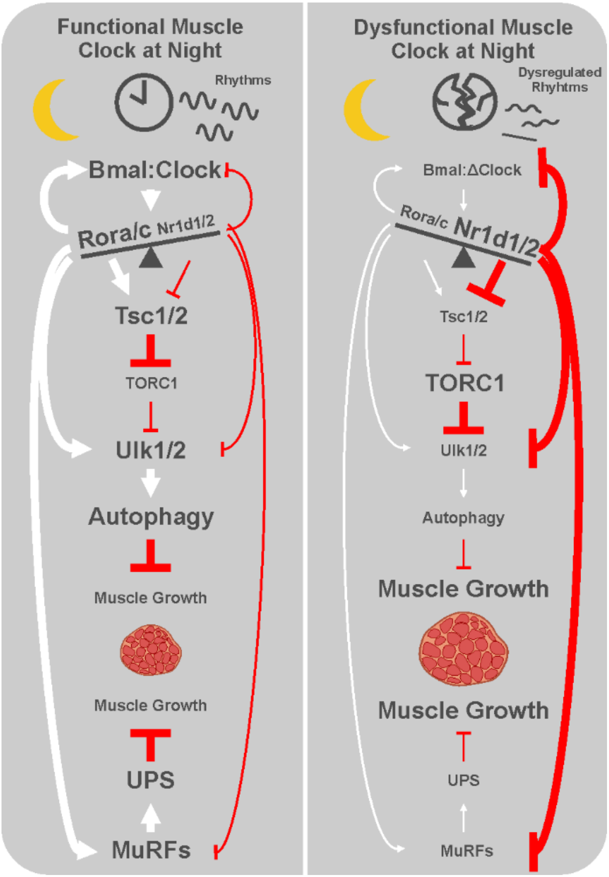

Fig. S13 Proposed model of circadian regulation of muscle growth by muscle peripheral clock. In entrained wild type larvae (left), at night the molecular circadian clock increases Rora/c transcriptional activator and lessens Nr1d1/2 (Rev-erb) transcriptional inhibitor leading to Tsc1/2 upregulation and TORC1 inhibition, thereby reducing suppression of Ulk1/2 and activating autophagy that limits muscle growth. Simultaneously at night, Rora/c:Nr1d1/2 balance activates MuRFs, targeting muscle proteins to the proteasome and limiting muscle growth. A defective intrinsic muscle clock (right, mΔCLK) swings the nocturnal Rora/c:Nr1d1/2 in favour of the transcriptional inhibitor Nr1d1/2, allowing TORC1 activation and MuRF inhibition and thereby enhancing muscle growth. The immediate consequence (not illustrated) of such aberrant growth is poor muscle function. Longer term, mΔCLK fish recover and survive well but shown reduced growth in later life.

Figure S14

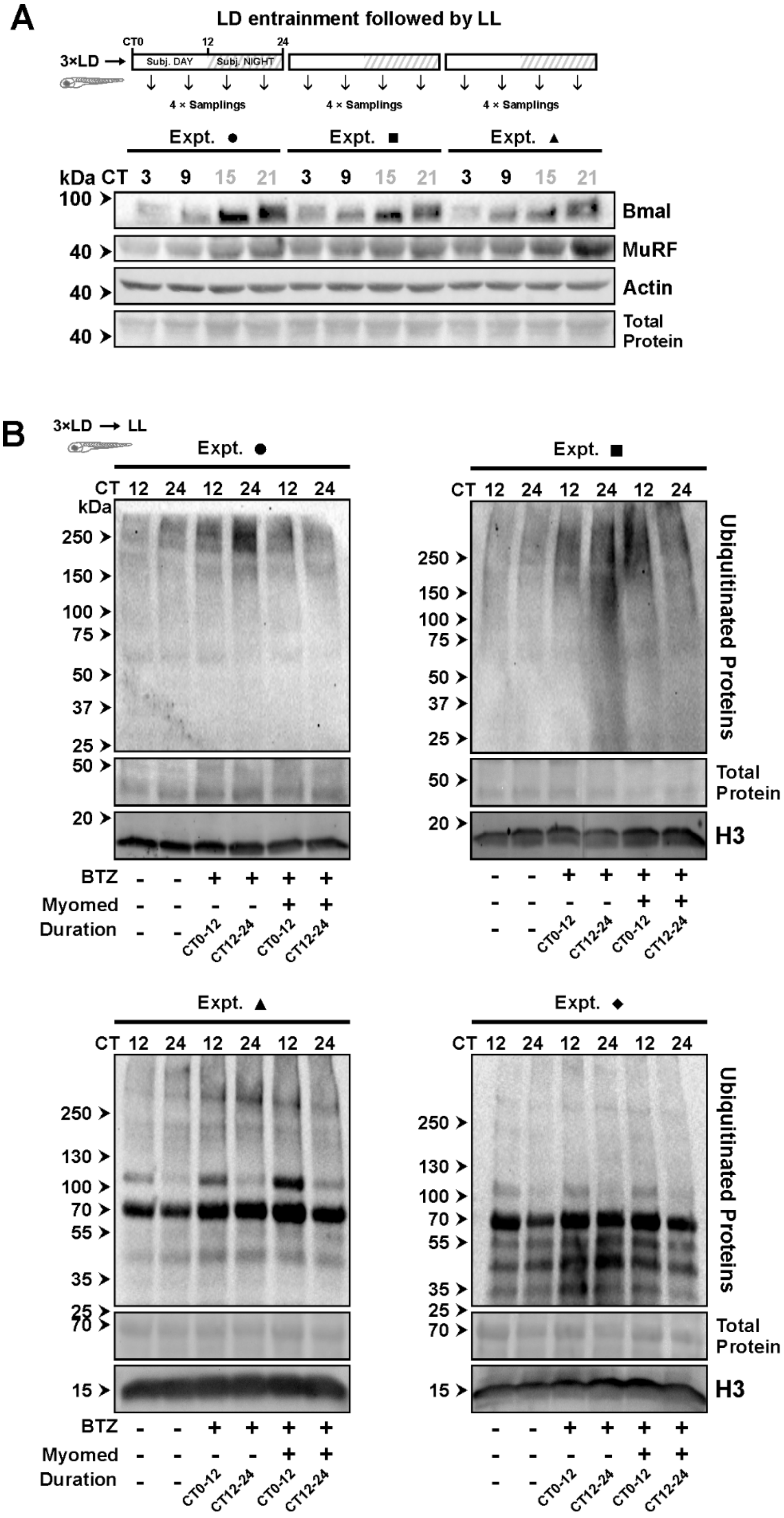

Fig. S14 Replicate blots for Fig. 2C (A) and 2D (B).

Figure S15

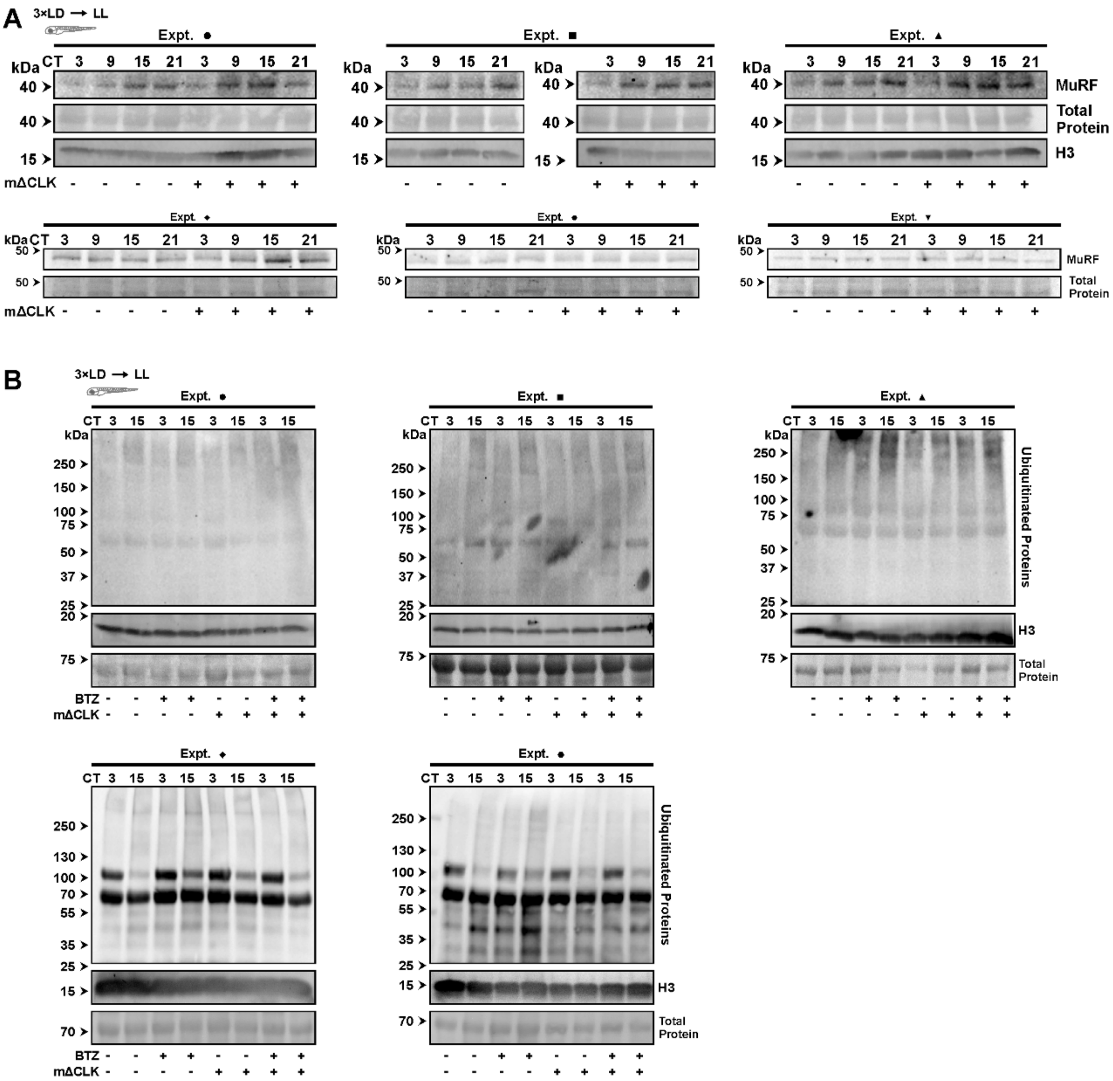

Fig. S15 Replicate blots for Fig. 3A (A) and 3B (B). Replicates Diamond, Hexagon, and Inverted Triangle in **(A)** share the same Myc-tagged ΔCLK and H3 blots as their corresponding replicates Diamond, Hexagon, and Inverted Triangle in **Figure S17A**.

Figure S16

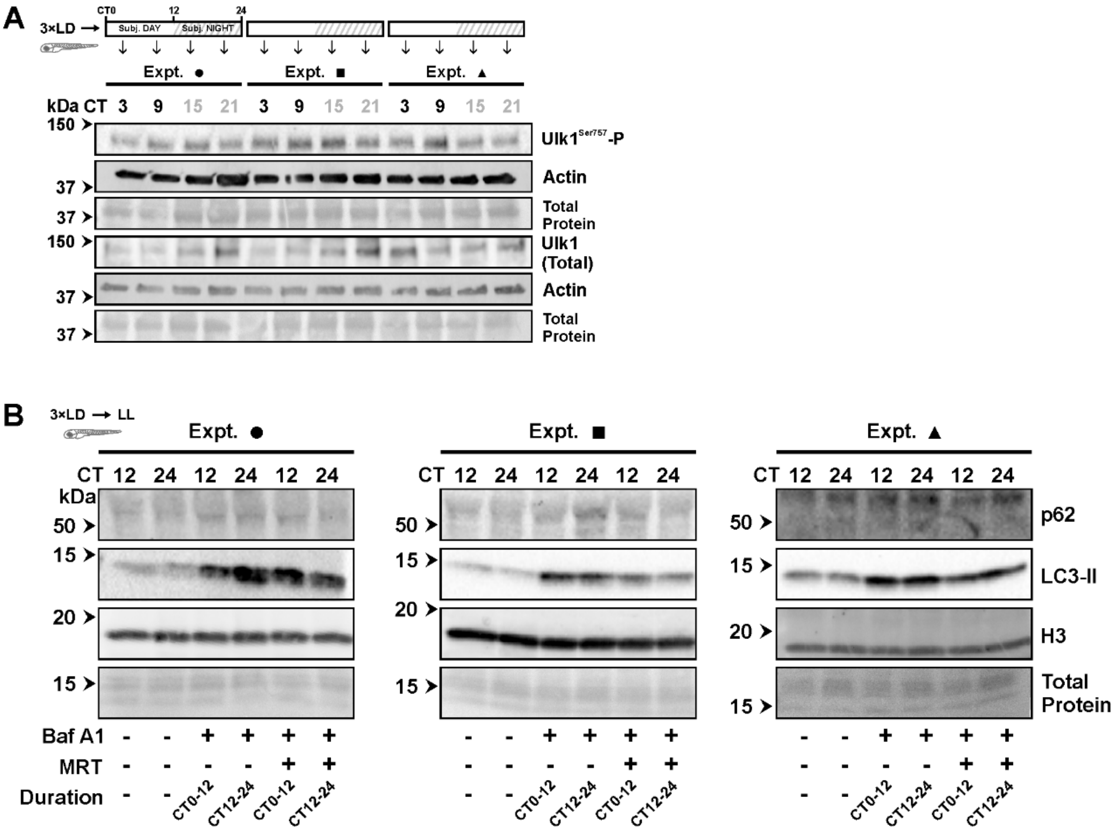

Fig. S16 Replicate blots for Fig. 4C (A) and 4D (B).

Figure S17

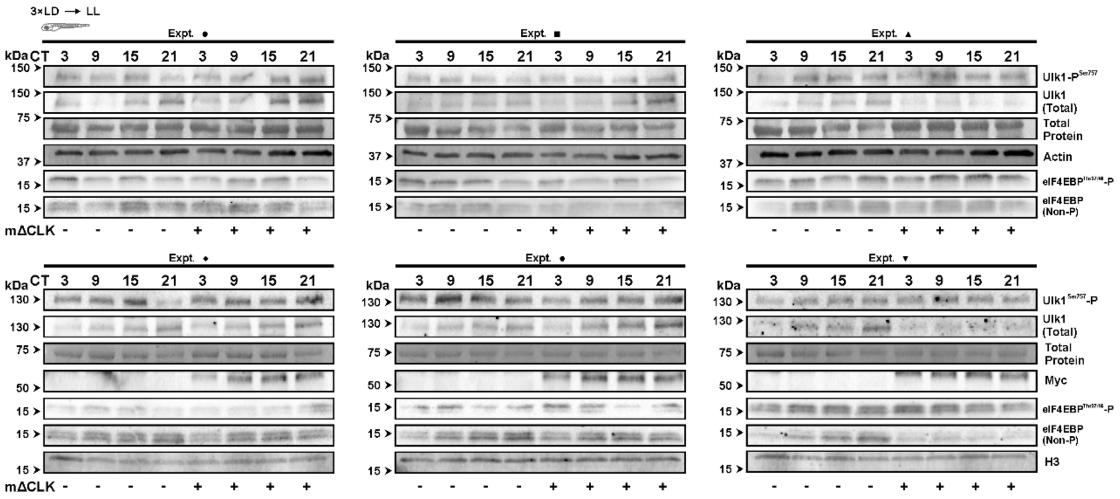

Fig. S17 Replicate blots for Fig. 5A.

Figure S18

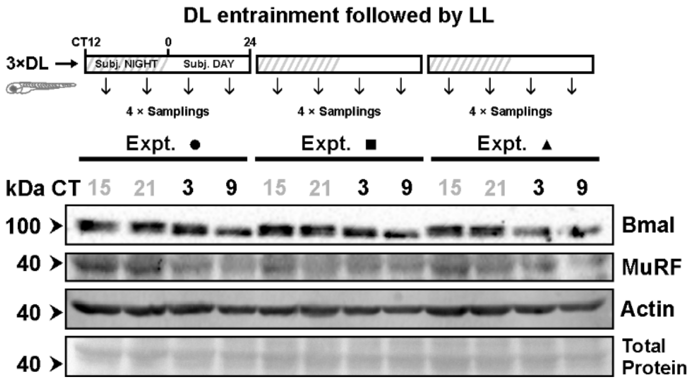

Fig. S18 Replicate blots for Fig. S5A.

Figure S19

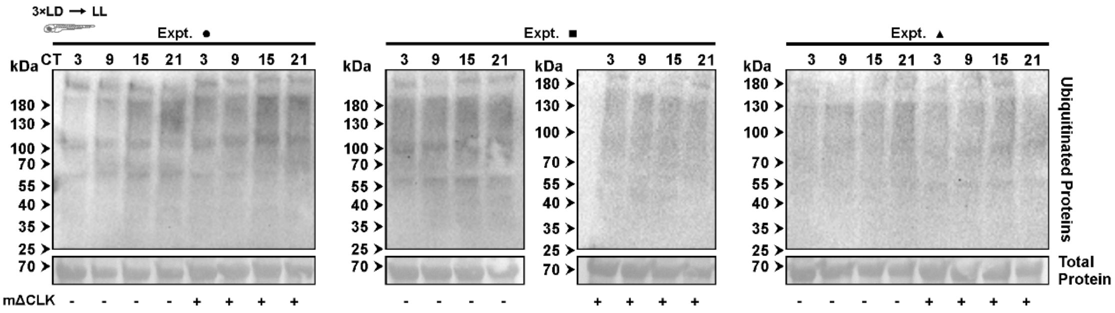

Fig. S19 Replicate blots for Fig. S6A.

Figure S20

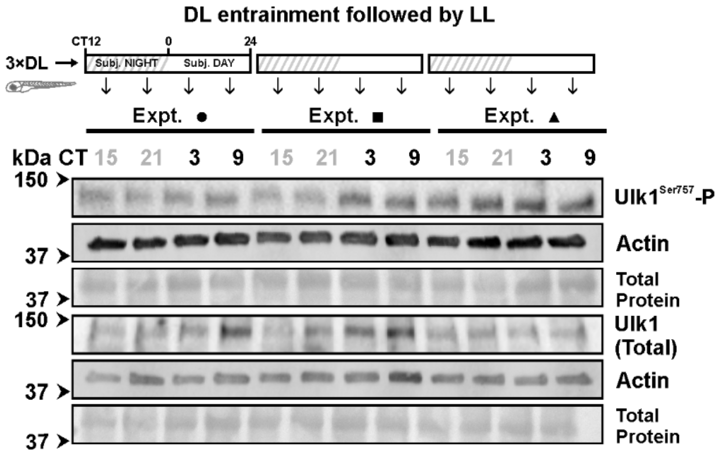

Fig. S20 Replicate blots for Fig. S7D.

Figure S21

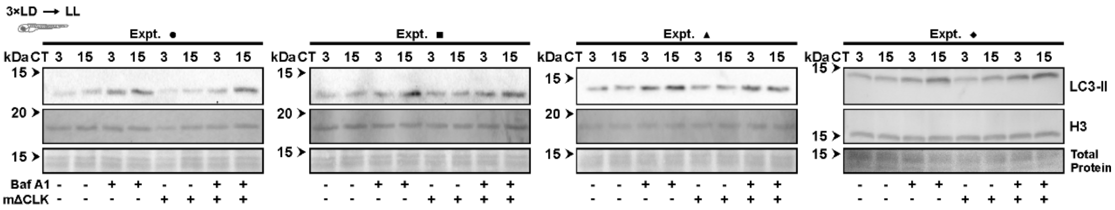

Fig. S21 Replicate blots for Fig. S9B.

Figure S22

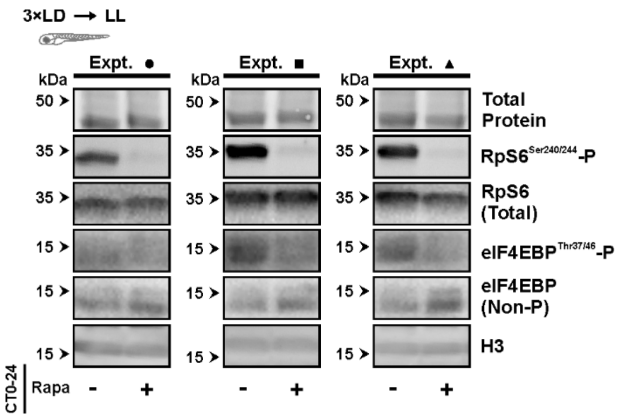

Fig. S22 Replicate blots for Fig. S10A.

Figure S23

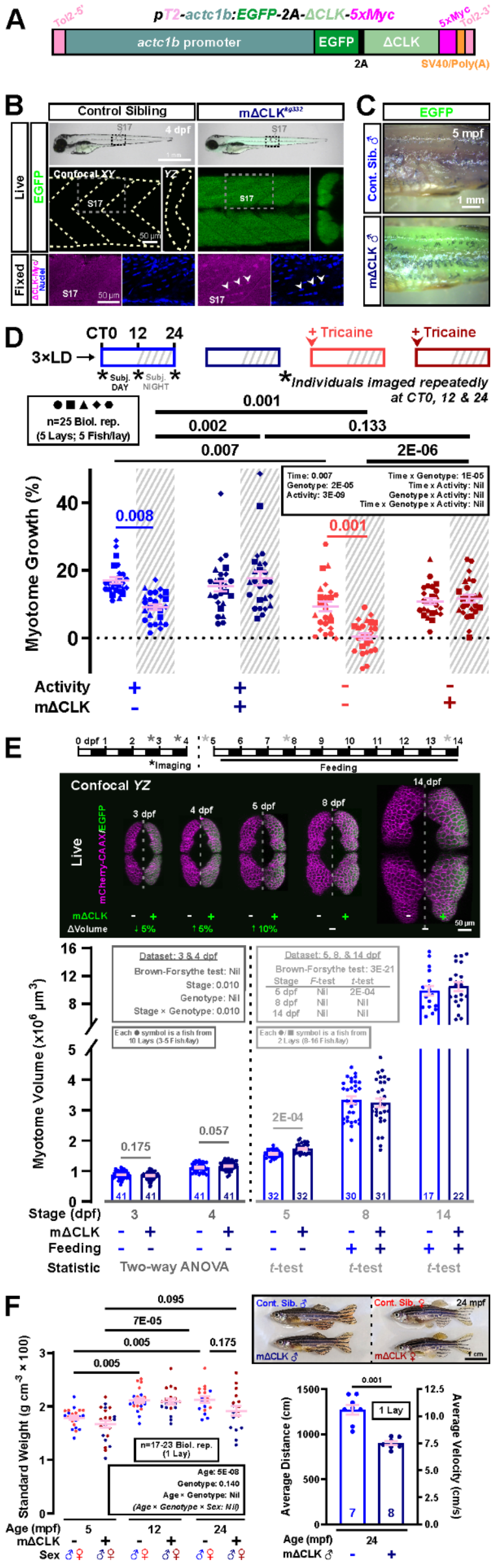

Fig. S23 False Positive Risk estimates for Fig. 1.

Figure S24

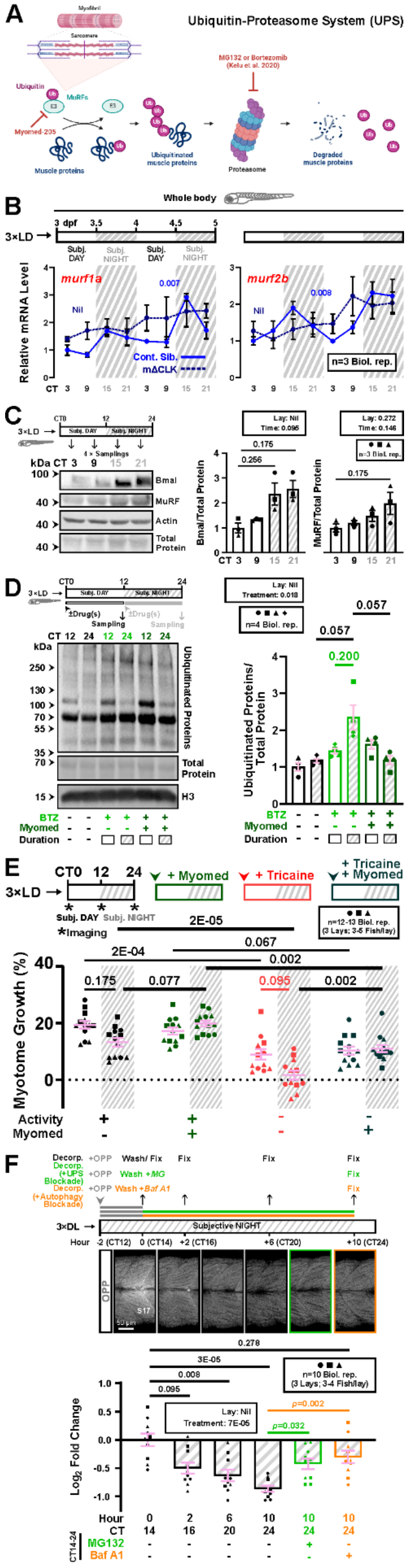

Fig. S24 False Positive Risk estimates for Fig. 2.

Figure S25

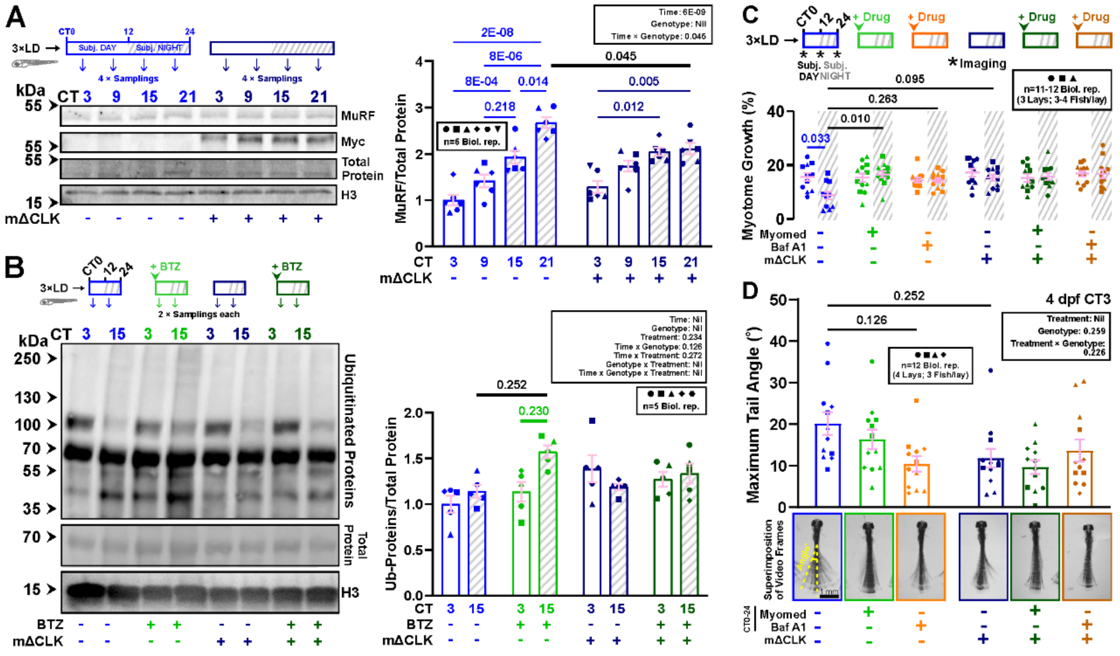

Fig. S25 False Positive Risk estimates for Fig. 3.

Figure S26

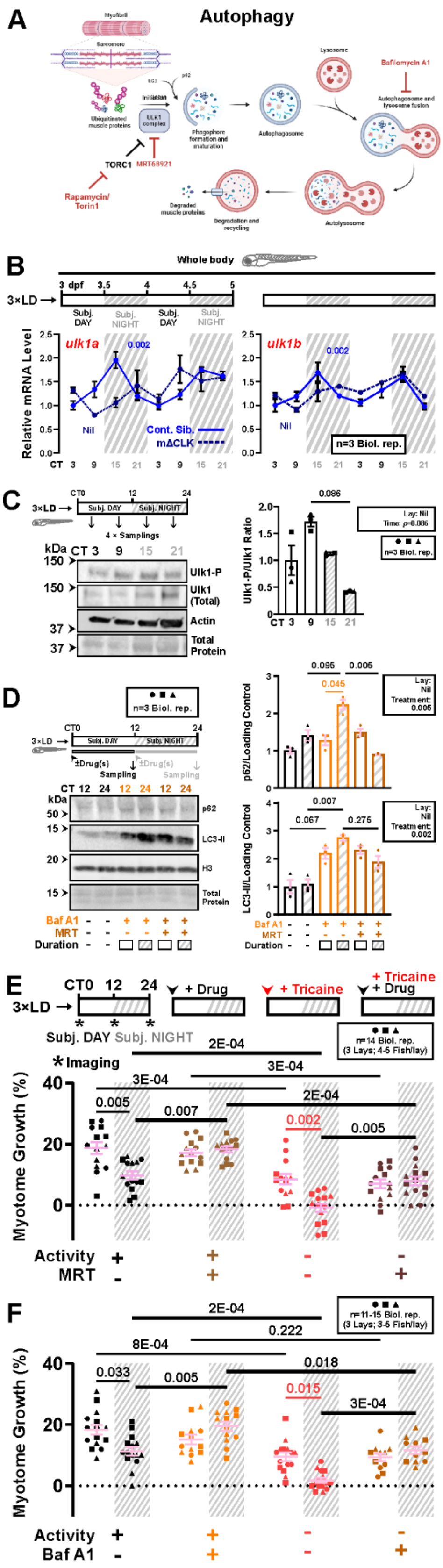

Fig. S26 False Positive Risk estimates for Fig. 4.

Figure S27

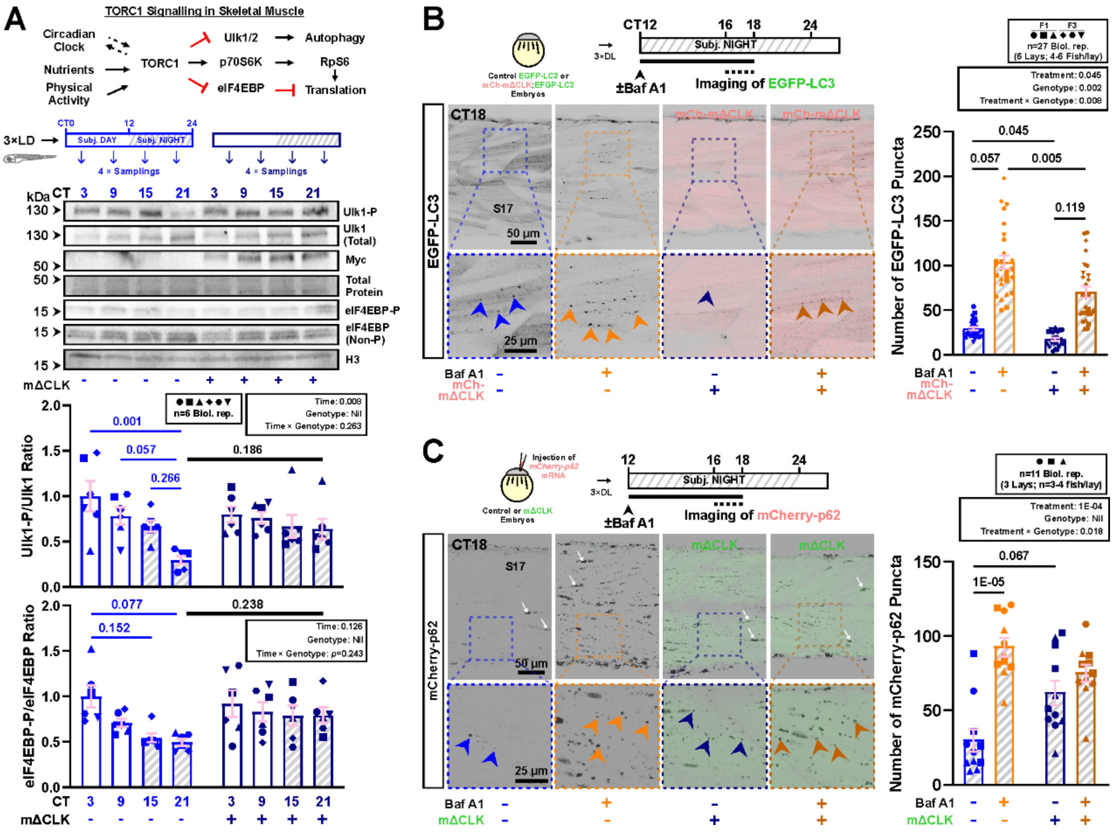

Fig. S27 False Positive Risk estimates for Fig. 5.

Figure S28

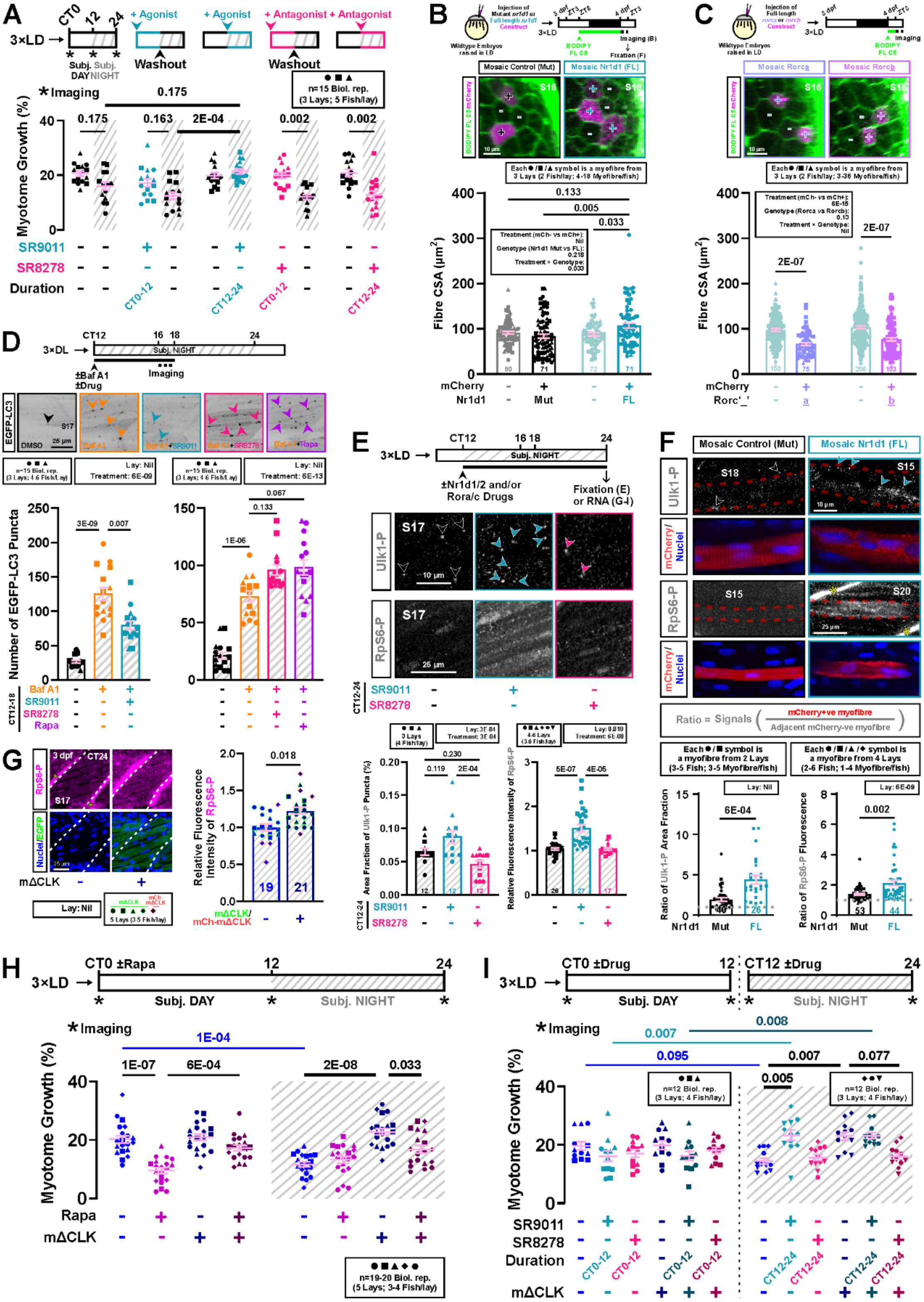

Fig. S28 False Positive Risk estimates for Fig. 6.

Figure S29

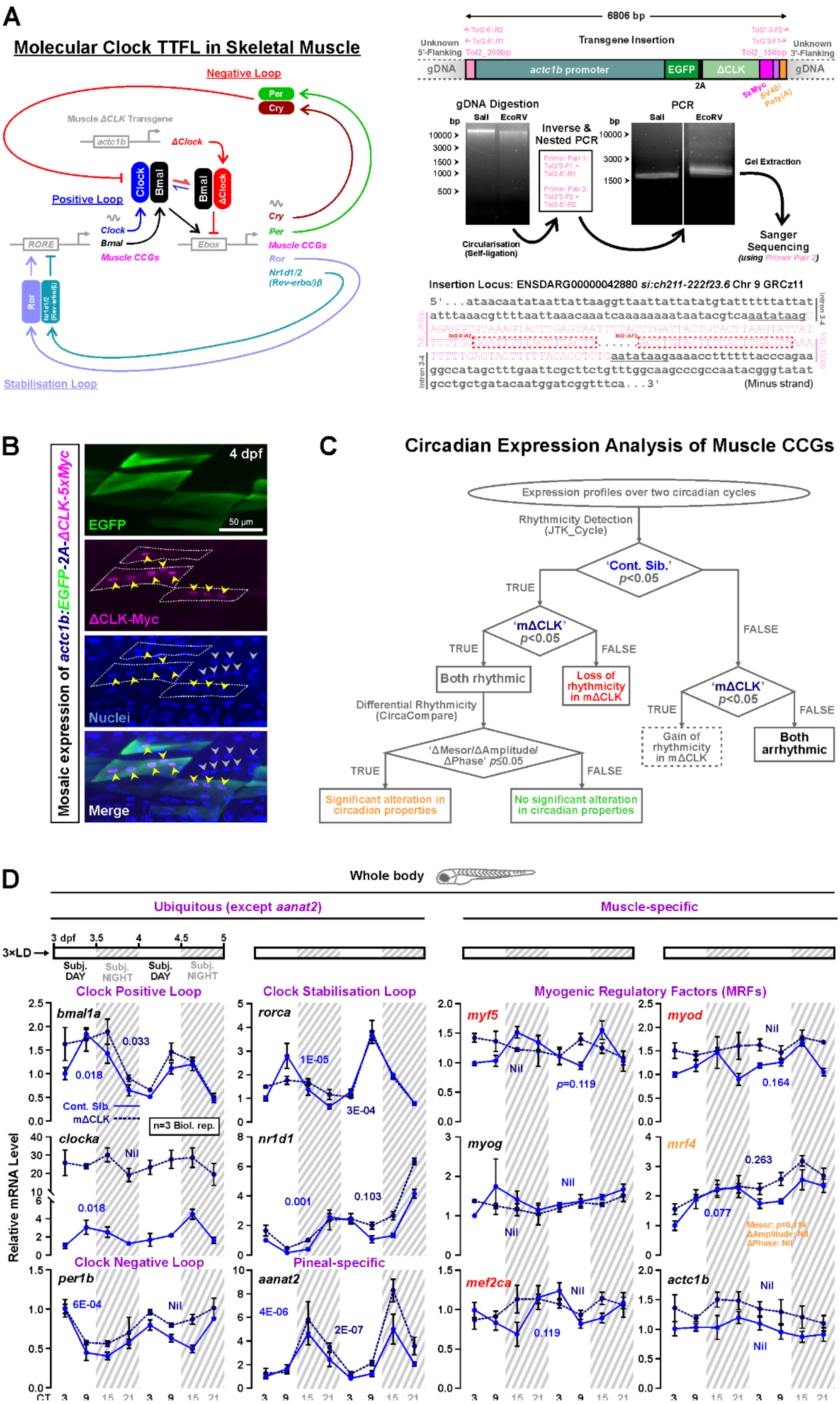

Fig. 29 False Positive Risk estimates for Fig. S1.

Figure S30

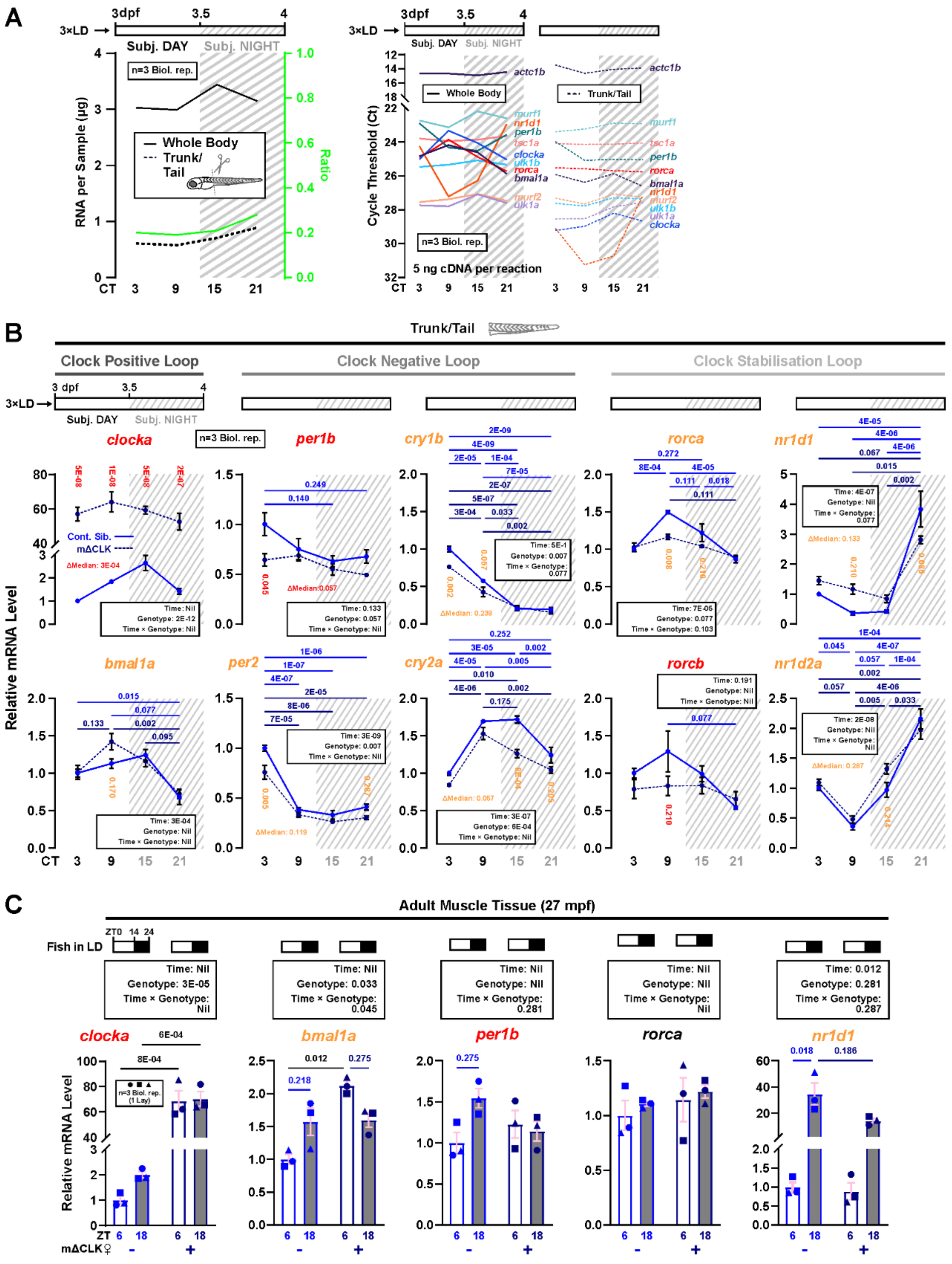

Fig. S30 False Positive Risk estimates for Fig. S2.

Figure S31

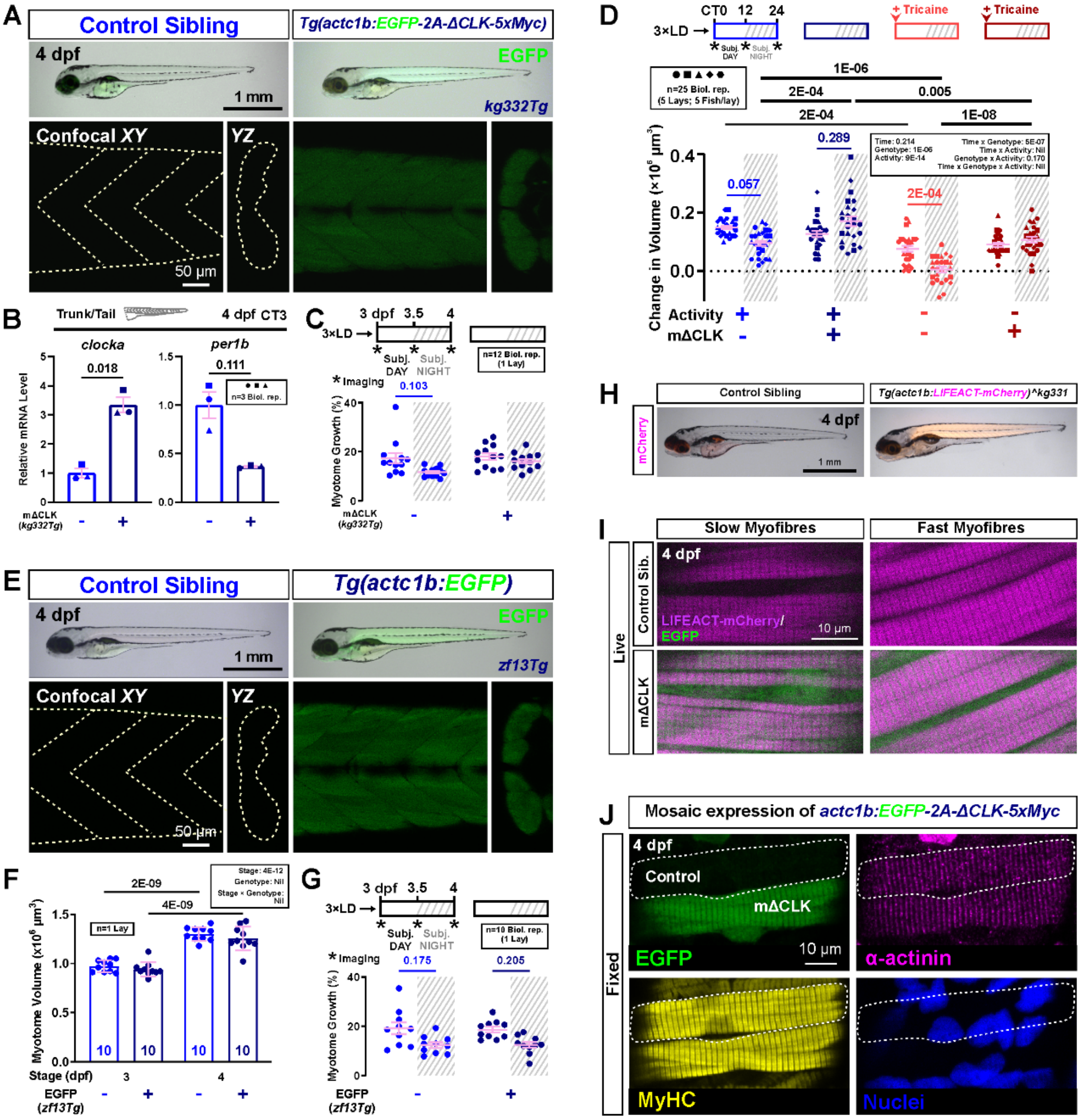

Fig. S31 False Positive Risk estimates for Fig. S3.

Figure S32

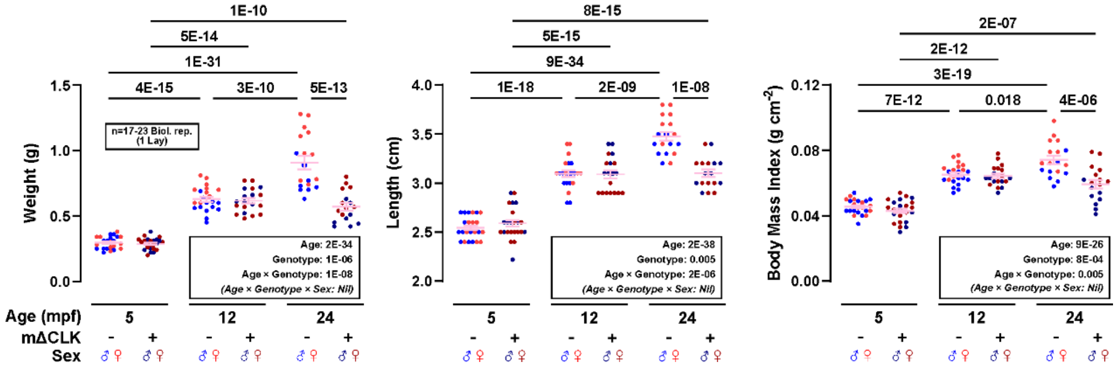

Fig. S32 False Positive Risk estimates for Fig. S4.

Figure S33

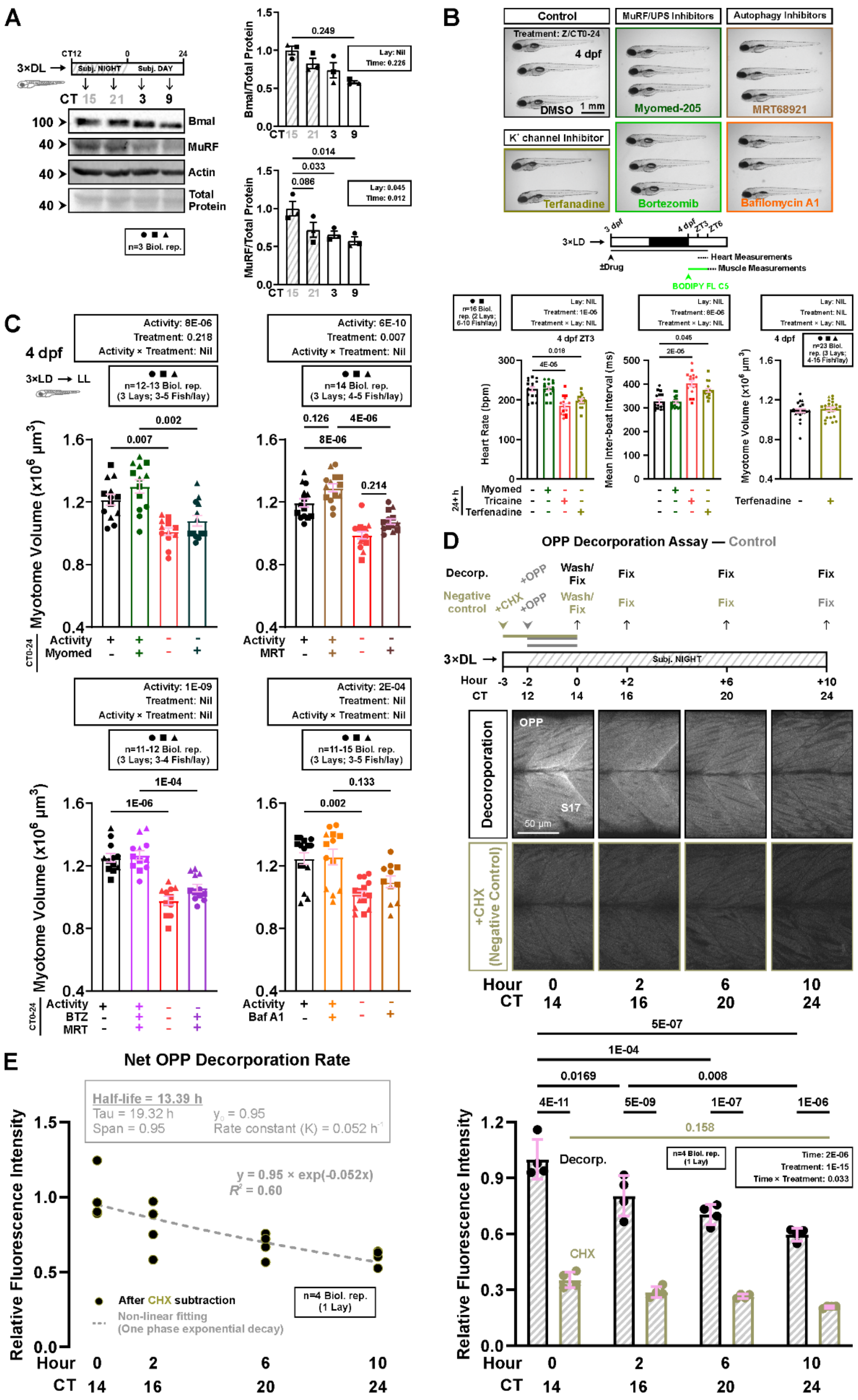

Fig. S33 False Positive Risk estimates for Fig. S5.

Figure S34

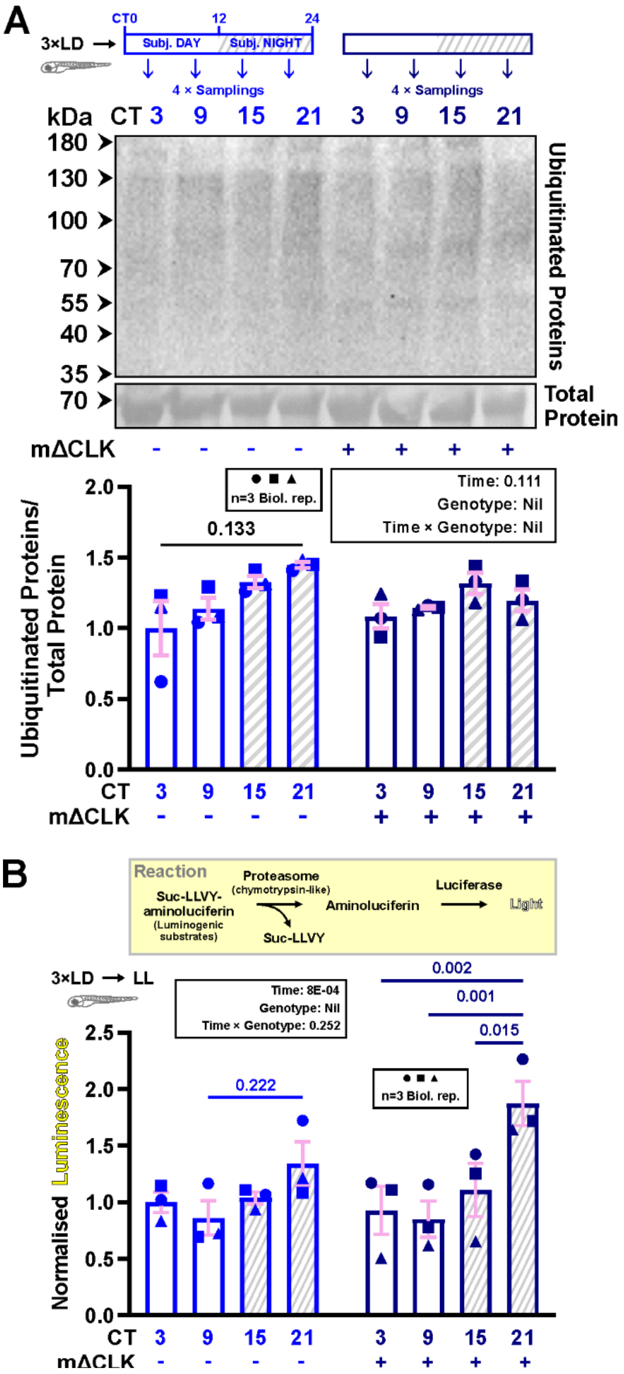

Fig. S34 False Positive Risk estimates for Fig. S6.

Figure S35

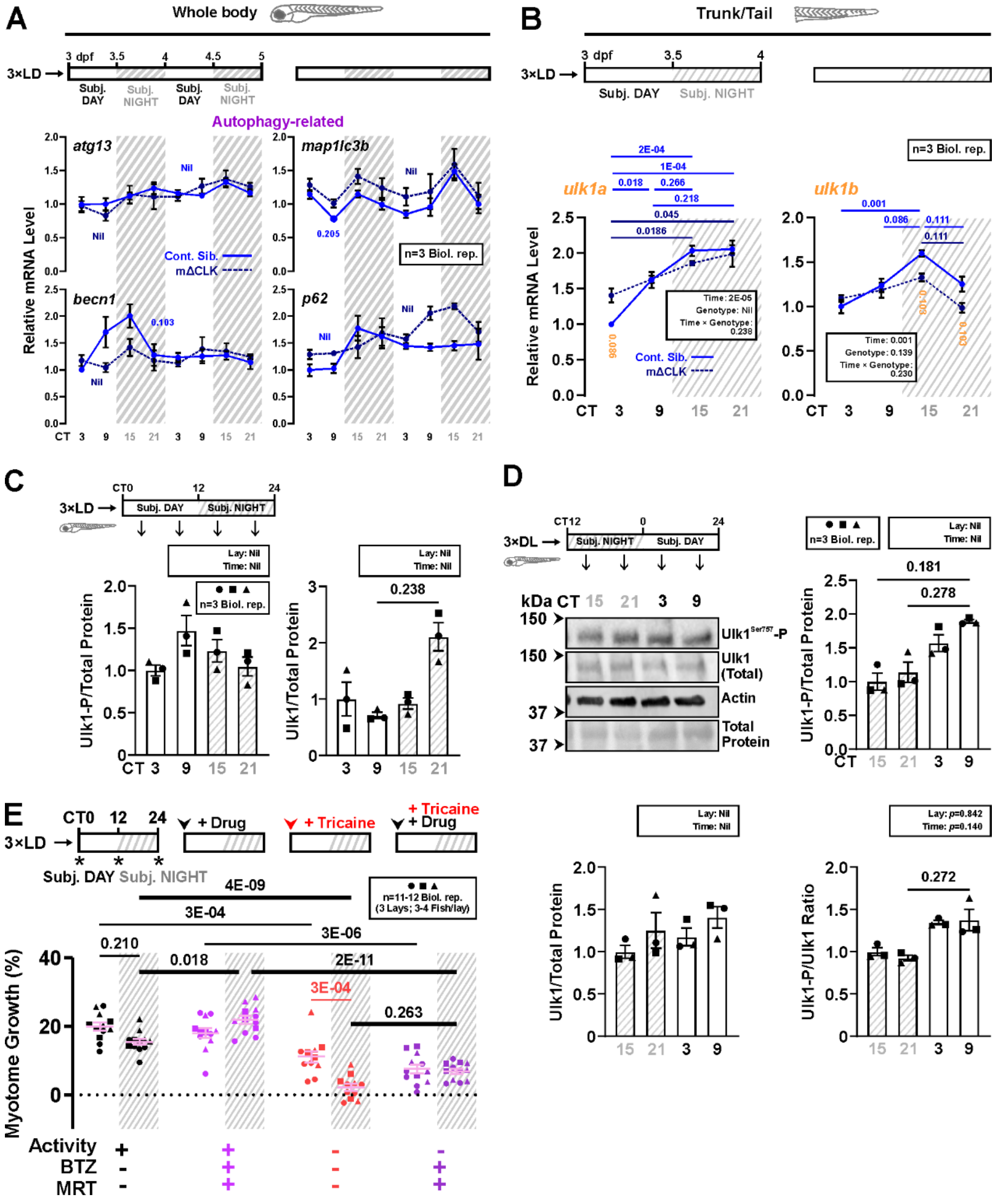

Fig. S35 False Positive Risk estimates for Fig. S7.

Figure S36

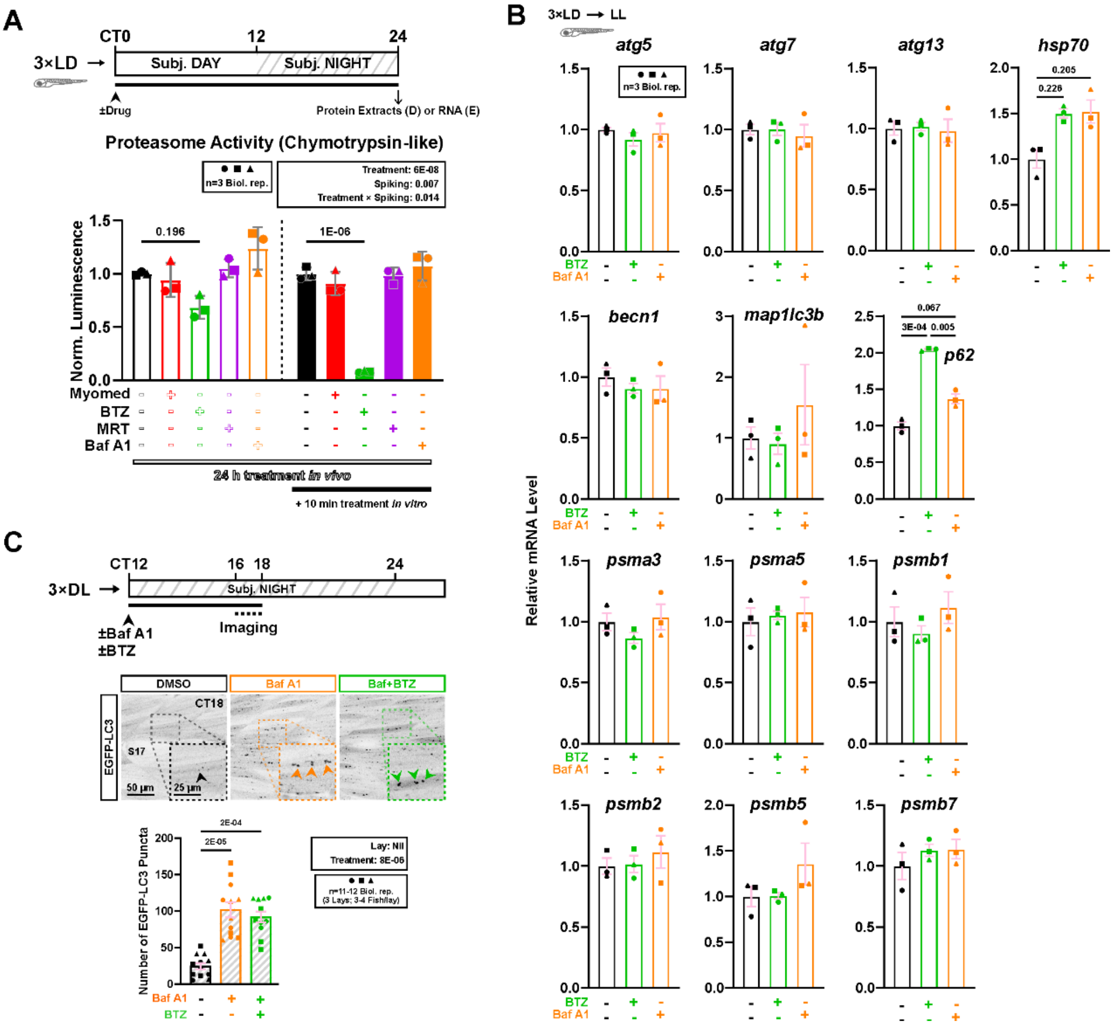

Fig. S36 False Positive Risk estimates for Fig. S8.

Figure S37

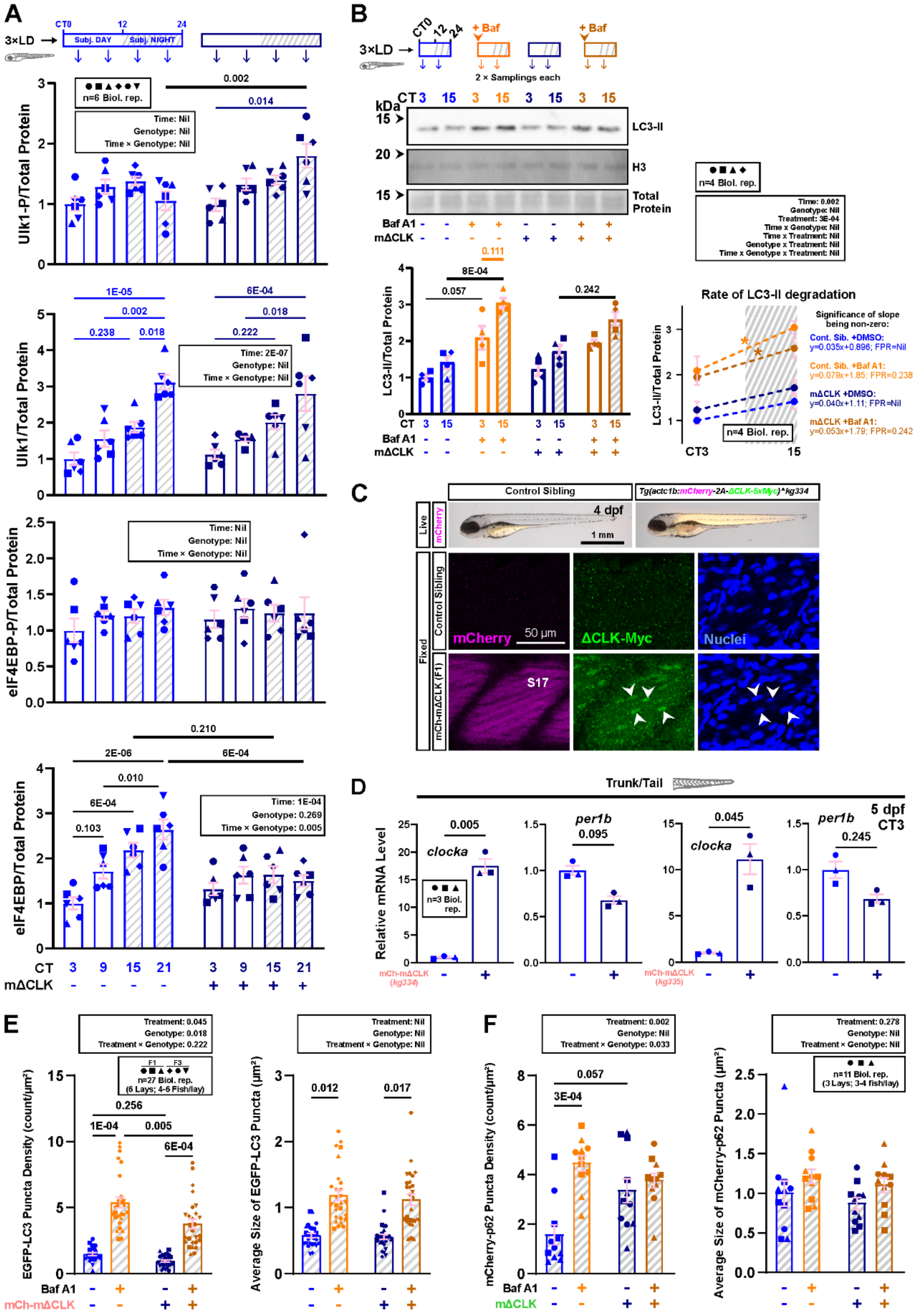

Fig. S37 False Positive Risk estimates for Fig. S9.

Figure S38

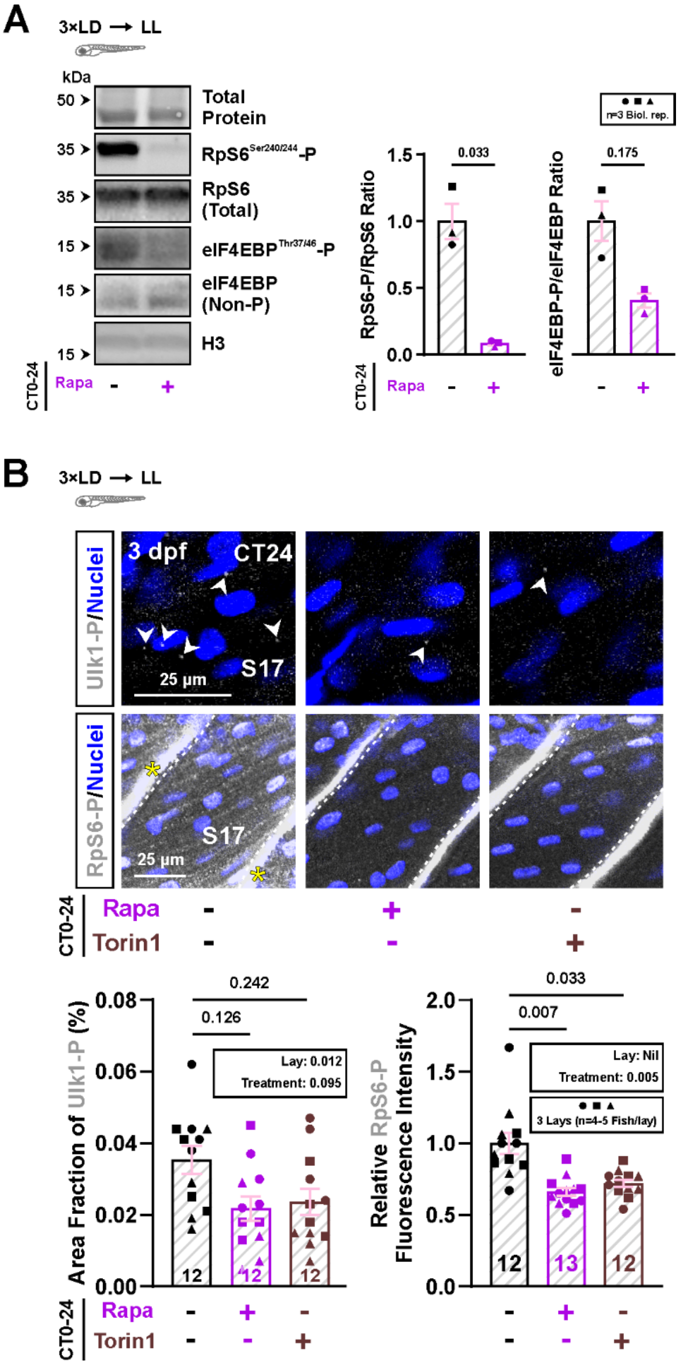

Fig. S38 False Positive Risk estimates for Fig. S10.

Figure S39

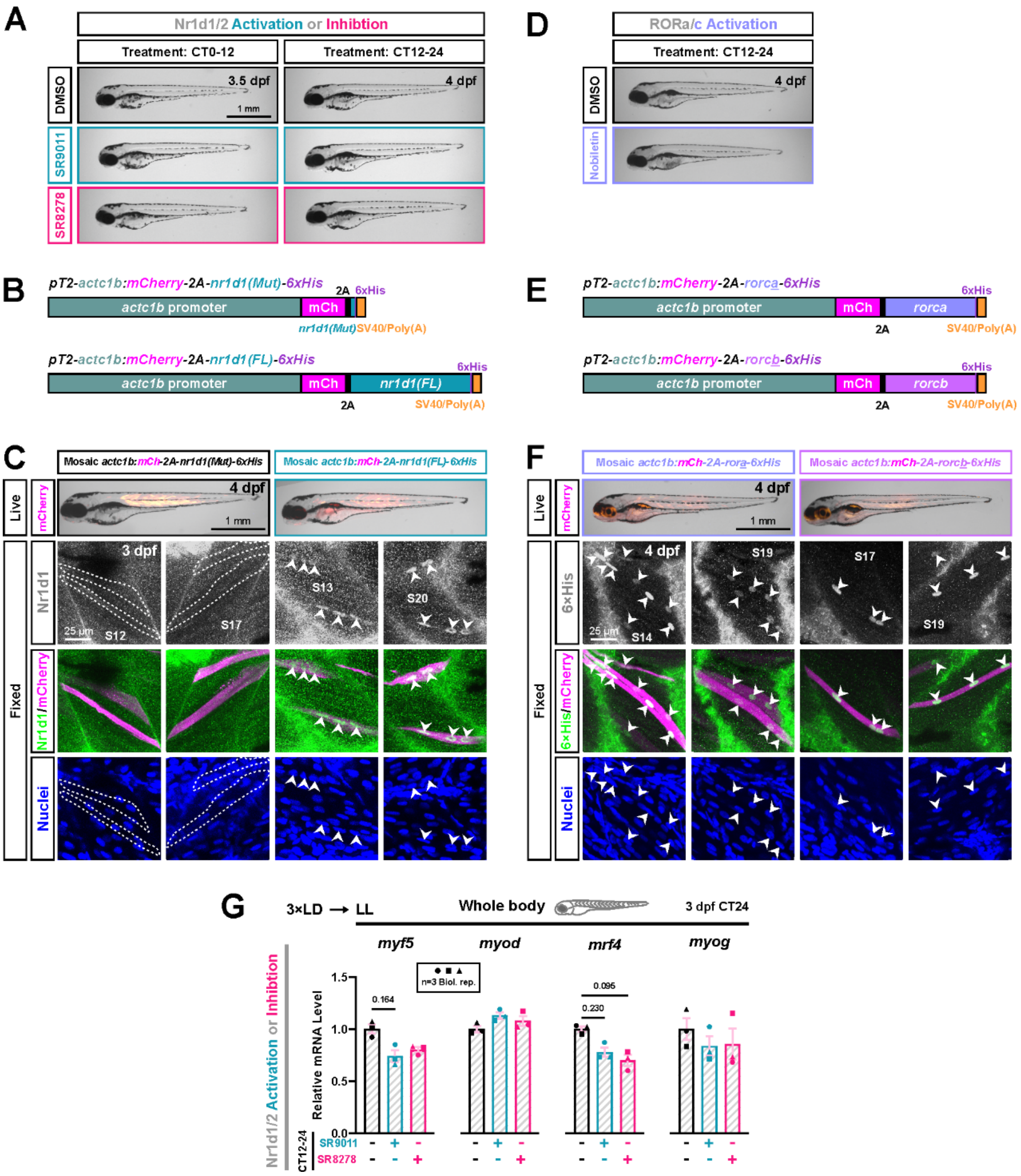

Fig. S39 False Positive Risk estimates for Fig. S11.

Figure S40

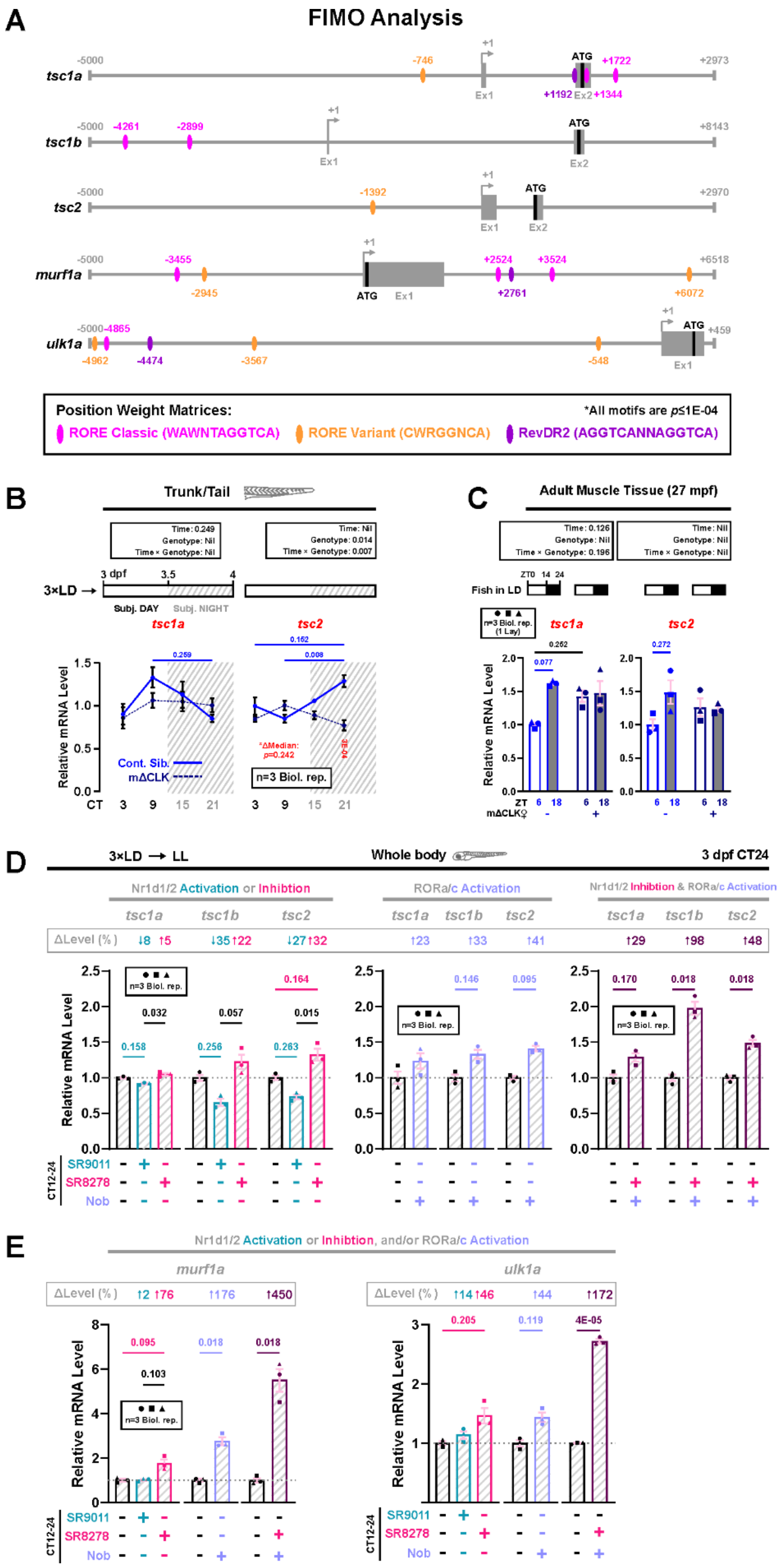

Fig. S40 False Positive Risk estimates for Fig. S12.

Table S1. List of clock mutant mouse models and their muscle phenotypes

Mutants	Transgenics	Muscle Phenotype(s)	Mechanism(s)	References
Bmal1^{tm1Bra}		Reduced muscle mass, myofibre CSA, and myofibre number *in vivo; sarcopenic-like*	-	Kondratov *et al.* (2006)
-	*Mlc1f-Cre*$^{tm1(cre)Sjb}$*; Bmal1fl/fl*^{tm1Weit}, *skeletal muscle knockout*	Reduced muscle force; increased muscle mass; reduced glucose uptake	BMAL1:CLOCK-mediated activation of *Glut* (glucose transporter) and/or regulation of Pyruvate dehydrogenase (PDH) activity	Dyar *et al.* (2014)
	Hsa-Cre-ERT2$^{97.16Mtz}$*; Bmal1fl/fl*^{tm1Weit,} *skeletal muscle inducible knockout*	Reduced glucose uptake; reduced glucose uptake		
	Mlc1f-Cre$^{tm1(cre)Sjb}$*; Bmal1fl/fl*^{tm1Weit}, *skeletal muscle knockout*	Increased lean mass to fat mass ratio; reduced neutral lipid storage, and accumulation of bioactive lipids and glucogenic amino acids; increased protein turnover	BMAL1 activation of *Dgat2* and **REV-ERBα-dependent repression** of major targets involved in lipid metabolism and **protein turnover (*MuRF-1, Atrogin-1*)**; increased oxidative stress, altered mitochondrial function, and metabolic inefficiency	Dyar et al. (2018)
	Hsa-MerCreMer^{2Kesr}*; Bmal1fl/fl*^{tm1Weit}, *skeletal muscle inducible knockout*	Reduced muscle force; increased oxidative fibre type; increased muscle fibrosis	-	Schroder *et al.* (2015)
		Reduced body weight; increased lean mass to fat mass ratio; increased fasting insulin level; increased non-fasted blood glucose level; reduced glucose uptake	BMAL1:CLOCK-mediated activation of *Glut* and *hexokinase 2 (Hk2)* and *phosphofructokinase 1 (Pfk1)*	Harfmann *et al.* (2015)
		Altered *titin* splicing; increased heterogeneity in sarcomere length	BMAL1:CLOCK-mediated activation of *Rbm20* (*tititn* splicing regulator)	Riley *et al.* (2022)
		Disrupted behavioural rhythms; reduced muscle force and mass; increased frailty; increased oxidative fibre type; reduced mitochondrial oxidative capacity	-	Fernández-Martínez *et al.* (2024)
	Hsa-Cre; Bmal1-stopFL^{tm1Saco}, *skeletal muscle reconstitution*	Reduced body weight; increased lean mass to fat mass ratio; reduced glucose uptake; glucose intolerant	-	Smith *et al.* (2023)
	Hsa-Cre; Bmal1-stopFL^{tm1Saco}, *skeletal muscle reconstitution*	Reduced body weight and myofibre CSA; increased muscle fibrosis; sarcopenic-like	-	Kumar *et al.* (2024)
ClockΔ19^{m1Jt}	-	Reduced muscle force and disrupted muscle architecture; reduced mitochondria volume and function	BMAL1:CLOCK-mediated activation of MyoD, *Pgc-1α* and *Pgc-1β*	Andrews *et al.* (2010)
Cry1$^{tm1Asn/tm1Asn}$		Promoted myoblast differentiation *in vitro* and accelerated muscle regeneration *in vivo*	-	Lowe *et al.* (2018)
Cry2$^{tm1Asn/tm1Asn}$		Suppressed myoblast differentiation *in vitro* and delayed muscle regeneration *in vivo*	CRY2-induced activation of *Ccnd1* (cell cycle entry) and *Tmem176b* (fusion), with cooperative stabilisation from Bclaf1	
Cry1$^{tm1Asn/tm1Asn}$; *Cry2*$^{tm1Asn/tm1Asn}$		Reduced lean mass, recuded soleus muscle mass, reduced myofibre cross-sectional area (CSA) in gastrocnemius/ plantaris/ soleus/ quadriceps muscle, and increased type I myofibres in plantaris muscle, *in vivo*	-	Jordan *et al.* (2017)
Per1$^{tm1Drw/tm1Drw}$		Suppressed myoblast differentiation *in vitro* and muscle regeneration *in vivo*	PER-induced activation of *Igf2* (proliferation)	Katoku-Kikyo *et al.* (2021)
Per2$^{tm1Drw/tm1Drw}$				
Per1$^{tm1Drw/tm1Drw}$; *Per2*$^{tm1Drw/tm1Drw}$				
Rev-erbα$^{-/-}$		Reduced body weight, increased CSA (type I, extensor digitorum longus muscle), myofibre type transformation transformation in soleus muscle, reduced TRα2 content, *in vivo*	REV-ERBα-mediated repression of *TRα1*/2	Pircher *et al.* (2005)
		Reduced exercise capacity, reduced muscle mitochondrial content and function, and induced autophagy, *in vivo*	REV-ERBα-mediated **repression of** mitochondrial and **autophagy gene expression**	Woldt *et al.* (2013)
		Reduced muscle mass and myofibre CSA *in vivo*	REV-ERBα-mediated **repression of atrophy-related and autophagy gene expression**	Mayeuf-Louchart *et al.* (2017)
		Promoted myoblast proliferation and differentiation, and augmented muscle regeneration and satellite cell proliferative expansion, *in vitro*	REV-ERBα-mediated repression of Wnt pathway gene expression and signalling activity	Chatterjee *et al.* (2019)
		Reduced muscle force and Ca^{2+} handling capacity *in vivo*	REV-ERBα-mediated repression of *Ryr1*, *Serca1*, *Serca2, and Mln* (Ca^{2+} signalling)	Boulinguiez *et al.* (2022)
Rev-erbα$^{+/tm1Ven}$		Increased myofibre CSA and accelerated muscle regeneration, *in vivo*	-	Welch *et al.* (2017)
		Increased body weight, lean mass, muscle force, and myofibre CSA, *in vivo*	-	Welch *et al.* (2020)
Rev-erbα$^{tm1Ven/tm1Ven}$		Increased myofibre CSA, reduced muscle regeneration, *in vivo*	REV-ERBα-mediated repression of myogenic factor expression	Welch *et al.* (2017)
		Reduced lean mass and muscle force, and increased myofibre CSA at 6-week but decreased myofibre CSA at 15-week, *in vivo*	REV-ERBα-mediated **repression of catabolic** and cellular stress **gene expression**	Welch *et al.* (2020)
-	RORαΔDE (lacking ligand-binding domain; dominant-negative), skeletal muscle overexpression	Reduced body weight, and induced hyperglycemia and glucose intolerance, *in vivo*	RORα-induced Akt2 expression and Akt signalling activity	Raichur *et al.* (2010)

References: (29-49)

Table S2. List of key resources

REAGENT or RESOURCE	SOURCE	IDENTIFIER
**Antibodies**		
6×His, mouse monoclonal IgG1	Proteintech	#66005
A4.1025 (pan myosin), mouse monoclonal IgG2a	DSHB	#A4.1025
Actin (pan), rabbit polyclonal	Sigma-Aldrich	#A2066
BMAL1, rabbit polyclonal	abcam	#ab93806
c-Myc (clone 9E10), mouse monoclonal	Bio-rad	#MCA2200GA
eIF4EBP (non-phospho; clone 87D12), rabbit monoclonal	Cell Signaling	#4923
eIF4EBP$^{Thr37/46}$-P (clone 236B4), rabbit monoclonal	Cell Signaling	#2855
GFP (cross reacts with EGFP), chicken polyclonal	abcam	#ab13970
Histone H3 protein, rabbit polyclonal	abcam	#ab1791
LC3B, rabbit polyclonal	Cell Signaling	#2775
mCherry, mouse monoclonal	Affinity Biosciences	#T0090
MuRF1/TRIM63, goat polyclonal	R&D Systems	#AF5366
NR1D1, rabbit polyclonal	Proteintech	#14506-1-AP
p62/SQSTM1, rabbit polyclonal	abcam	#ab155686
RFP (cross reacts with mCherry), rabbit polyclonal	MBL	#PM005
RpS6 ribosomal protein (total; clone 54D2), mouse monoclonal	Cell Signaling	#2317
RpS6$^{Ser240/244}$-P (clone D68F8), rabbit monoclonal	Cell Signaling	#5364
Ubiquitin (clone FK2), mouse monoclonal	Calbiochem	#ST1200
ULK1 (clone D8H5), rabbit monoclonal	Cell Signaling	#8054
Ulk1^{Ser757}-P (clone D7O6U), rabbit monoclonal	Cell Signaling	#14202
α-actinin (clone EA-53), mouse monoclonal IgG1	Sigma-Aldrich	#A7732
Secondary, Alexa Fluor™ 488 goat anti-mouse IgG1	Invitrogen	#A21121
Secondary, Alexa Fluor™ 488 goat anti-chicken IgY(H+L)	Invitrogen	#A11039
Secondary, Alexa Fluor™ 488 goat anti-mouse IgG(H+L)	Invitrogen	#A11001
Secondary, Alexa Fluor™ 488 goat anti-rabbit IgG(H+L)	Invitrogen	#A11008
Secondary, Alexa Fluor™ 555 goat anti-mouse IgG(H+L)	Invitrogen	#A21422
Secondary, Alexa Fluor™ 568 goat anti-mouse IgG2a	Invitrogen	#A21134
Secondary, Alexa Fluor™ 633 goat anti-rabbit IgG(H+L)	Invitrogen	#A21071
Secondary, Amersham™ ECL™ HRP sheep anti-mouse	GE Healthcare	#NA931
Secondary, Amersham™ ECL™ HRP donkey anti-rabbit	GE Healthcare	#NA934
Secondary, HRP rabbit anti-goat IgG(H+L)	abcam	#ab6741

**Bacterial strains**		
5-alpha Competent *E. coli* (High Efficiency)	NEB	#C2987
Mix & Go Competent Cells - Strain DH5 Alpha	Zymo Research	#T3007
Subcloning Competent *E. coli Cells*	NEB	#C2988
**Chemicals, peptides, and recombinant proteins**		
1-Bromo-3-chloropropane	Sigma-Aldrich	#B9673
2-Mercaptoethanol	Bio-rad	#161-0710
Agarose	Sigma-Aldrich	#A9539
Agarose, low gelling temperature	Sigma-Aldrich	#A9414
Ampicillin sodium	BioVision	#2484
Blotting-grade blocker, non-fat dry milk	Bio-rad	#170-6404
Bovine serum albumin (BSA)	Sigma-Aldrich	#A7906
Dimethyl sulfoxide (DMSO)	Sigma-Aldrich	#D8418
Drug, Bafilomycin A1	Cayman	#11038
Drug, Bafilomycin A1	MP Biomedicals	#0215955580
Drug, Bortezomib	ApexBio	#A2614
Drug, Cycloheximide	Sigma-Aldrich	#C4859
Drug, MG132	Alfa Aesar	#J63250
Drug, MRT68921	Sigma-Aldrich	#SML1644
Drug, Myomed-205	Labeit Lab	N/A
Drug, Nobiletin	Cayman	#15421
Drug, Rapamycin	Enzo Life Sciences	#BML-A275
Drug, SR8278	Cayman	#17000
Drug, SR9011	Sigma-Aldrich	#SML2067
Drug, Terfenadine	Sigma-Aldrich	#T9652
Drug, Torin1	ApexBio	#A8312
Ethanol	Sigma-Aldrich	#32221-M
Ethyl 3-aminobenzoate methanesulfonate salt (MS222/ Tricaine)	Sigma-Aldrich	#A5040
Formamide	Sigma-Aldrich	#47671
Hydrogen peroxide solution, 30% (w/w) in H2O, contains stabilizer	Sigma-Aldrich	#H1009
Isopropanol, 99.5+%, extra pure	Thermo Scientific	#389710025
Laemmli sample buffer (4×)	Bio-rad	#161-0747
LB Broth	Sigma-Aldrich	#3022
Luria agar base	Sigma-Aldrich	#2025
Kanamycin sulfate	Sigma-Aldrich	#60615
Methanol	Sigma-Aldrich	#34860
Methyl cellulose	Sigma-Aldrich	#M0387
N-ethylmaleimide (NEM)	Sigma-Aldrich	#E3876
Oxoid™ Phosphate buffered saline (PBS) tablets	Thermo Scientific	#BR0014G
Paraformaldehyde, 4% in PBS	Alfa Aesar	#J61899
Phenol/chloroform/isoamyl alcohol (25:24:1), stabilized, saturated with 100 mM Tris-EDTA to pH 8.0	Thermo Scientific	#327111000
Phenol red solution	Sigma-Aldrich	#P0290
Phenylmethylsulfonyl fluoride (PMSF)	Calbiochem	#52332
Ponceau S	Fisher Scientific	#BP103-10

Sodium azide solution, 10% (w/v)	Severn Biotech	#40-2010-01
Tris-glycine SDS running buffer (10×)	Cell Signaling	#4050
Tris-glycine transfer buffer (10×)	Cell Signaling	#12539
Triton™ X-100	Sigma-Aldrich	#X100
TWEEN® 20	Sigma-Aldrich	#P1379
UltraPure™ SSC buffer (20×)	Invitrogen	#15557-044
Water	Sigma-Aldrich	#W4502
**Critical commercial assays/ reagents**		
Amersham™ ECL™ Prime Western Blotting Detection Reagent	GE Healthcare	#RPN2232
BODIPY™ FL C5-Ceramide	Invitrogen	#C10456
Click-iT™ Plus OPP Alexa Fluor™ 488 Protein Synthesis Assay Kit	Invitrogen	#C10456
cOmplete™, Mini Protease Inhibitor Cocktail tablets	Roche	#04693124001
DirectPCR Lysis Reagent	Viagen Biotech	#101-T
Exo-Cip™ Rapid PCR Cleanup Kit	NEB	#E1050
High Capacity cDNA Reverse Transcription Kit	Applied Biosystems	#4368814
Hoechst 33342, trihydrochloride, trihydrate	Invitrogen	#H3570
mMESSAGE mMACHINE™ SP6 Transcription Kit	Invitrogen	#AM1340
NEBuilder® HiFi DNA Assembly Master Mix	NEB	#E2621
PCRBIO HS VeriFi™DNA Polymerase	PCR Biosystems	#PB10.42-01
Proteasome-Glo™ Chymotrypsin-Like Cell-Based Assay	Promega	#G8660
Proteinase K	Fisher BioReagents	#BP1700-50
PureLink™ Quick Gel Extraction Kit	Invitrogen	#K210012
QIAprep® Spin Miniprep Kit	Qiagen	#27106
QIAquick® PCR Purification Kit	Qiagen	#28106
Restriction enzyme, AgeI-HF	NEB	#R3552
Restriction enzyme, EcoRV-HF	NEB	#R3195
Restriction enzyme, NotI-HF	NEB	#R3189
Restriction enzyme, SalI	NEB	#R0138
Restriction enzyme, XhoI	NEB	#R0146
RNA Clean & Concentrator™-25	Zymo Research	#R1017
RNase-Free DNase Set	Qiagen	#79254
RNase Inhibitor, murine	NEB	#M0314
RNeasy™ Mini Kit	Qiagen	#74106
RQ1 RNase-Free DNase	Promega	#M6101
T4 DNA Ligase	NEB	#M0202
Takyon™ Low Rox SYBR® MasterMix dTTP Blue	Eurogentec	#UF-LSMT-B0701
Tissue Extraction Reagent I	Invitrogen	#FNN0071
Tri-reagent	Sigma-Aldrich	#T9424
**Experimental models: Organisms/strains**		
Zebrafish/ AB wildtype	KCL Fish Facility	N/A
Zebrafish/ *Tg(actc1b:mCherryCAAX)*^{pc22}	(3)	N/A

Zebrafish/ *Tg(actc1b:EGFP)*^{zf13}	(4)	N/A
Zebrafish/ *Tg(actc1b:EGFP-2A-ΔCLK-5xMyc)*^{kg332}	This paper	N/A
Zebrafish/ *Tg(actc1b:EGFP-2A-ΔCLK-5xMyc)*^{kg333}	This paper	N/A
Zebrafish/ *Tg(actc1b:LIFEACT-mCherry)*^{kg331}	This paper	N/A
Zebrafish/ *Tg(actc1b:mCherry-2A-ΔCLK-5xMyc)*^{kg334}	This paper	N/A
Zebrafish/ *Tg(actc1b:mCherry-2A-ΔCLK-5xMyc)*^{kg335}	This paper	N/A
Zebrafish/ *Tg(CMV:EGFP-map1lc3b)*^{zf155}	(5)	N/A
**Oligonucleotides**		
Cloning, actc1b-F: 5'-ggagatcacttgggcccggcGCACGCGCAGATTCCGTG-3' (to clone from *pT2-actc1b:mCherryCAAX* and then insert into *pT2-EGFP-2A-ΔCLK* backbone)	This study	N/A
Cloning, actc1b-R: 5'-cttgctcaccatggtggcgaCCTTGGTCTGTGCAGGAC-3' (to clone from *pT2-actc1b:mCherryCAAX* and then insert into *pT2-EGFP-2A-ΔCLK* backbone)		
Cloning, LIFEACT-EGFP-F: 5'-aaagcaggctccaccATGGGCGTGGCCGACCTG-3' (to clone from *pME-LIFEACT-EGFP* backbone and insert into *pT2-actc1b* backbone)	This study	N/A
Cloning, LIFEACT-EGFP-R: 5'-acaagaaagctgggtcTTACTTGTACAGCTCGTCCATGCCG-3' (to clone from *pME-LIFEACT-EGFP* backbone and insert into *pT2-actc1b* backbone)		
Cloning, mCherry-F1: 5'-cacagaccaaggtcgccaccATGGTGAGCAAGGGCGAG-3' (to clone from *pT2-actc1b:mCherryCAAX* and then insert into *pT2-actc1b:2A-ΔCLK* backbone)	This study	N/A
Cloning, mCherry-R1: 5'-aagttcgtggctccggagccCTTGTACAGCTCGTCCATGC-3' (to clone from *pT2-actc1b:mCherryCAAX* and then insert into *pT2-actc1b:2A-ΔCLK* backbone)		
Cloning, mCherry-F2: 5'-gcgaccccccgtggccaccATGGTGAGCAAGGGCGAG-3' (to clone from *pT2-actc1b:mCherryCAAX* and then insert into *pT2-actc1b:LIFEACT* backbone)	This study	N/A
Cloning, mCherry-R2: 5'-tacaagaaagctgggtcttaCTTGTACAGCTCGTCCATGC-3' (to clone		

from *pT2-actc1b:mCherryCAAX* and then insert into *pT2-actc1b:LIFEACT* backbone)		
Cloning, nr1d1-F: 5'-tggaagaaaaccccggtcctATGACTTTACTGGGGCTC-3' (to clone from cDNA library and then insert into *pT2-actc1b:mCherry-2A* backbone)	This study	N/A
Cloning, nr1d1-R: 5'-cgcccaagctctccatttcaatgatgatgatgatgatgGGCATCAATGCGGAAAGAC-3' (to clone from cDNA library and then insert into *pT2-actc1b:mCherry-2A* backbone)		
Cloning, p62-F: 5'-AGTAGGAAACGCATTTTCAC-3' (to clone from cDNA library and then insert into *pCS-mCherry* backbone)	This study	N/A
Cloning, p62-R: 5'-ACCCTCCAGGTTTATGCTTG-3' (to clone from cDNA library and then insert into *pCS-mCherry* backbone)		
Cloning, rorca-F: 5'-tggaagaaaaccccggtcctATGAGAGCTCAAATTGAAGTAATCCC-3' (to clone from cDNA library and then insert into *pT2-actc1b:mCherry-2A* backbone)	This study	N/A
Cloning, rorca-R: 5'-cgcccaagctctccatttcaatgatgatgatgatgatgGCCTTCGGTGGAGTCAGG-3' (to clone from cDNA library and then insert into *pT2-actc1b:mCherry-2A* backbone)		
Cloning, rorcb-F: 5'-tggaagaaaaccccggtcctATGAGAGCCCAAATCGAG-3' (to clone from cDNA library and then insert into *pT2-actc1b:mCherry-2A* backbone)	This study	N/A
Cloning, rorcb-R: 5'-cgcccaagctctccatttcaatgatgatgatgatgatgACTGTTGGTGGAGTCTGG-3' (to clone from cDNA library and then insert into *pT2-actc1b:mCherry-2A* backbone)		
Cloning, pCS-mCherry-F1: 5’-ctcgagcctctagagccaccATGGTGAGCAAGGGCGAG-3’ (to linearise *pCS-mCherryCAAX* backbone without *CAAX*)	This study	N/A
Cloning, pCS-mCherry-R1: 5’-GGTGGCTCTAGAGGCTCG-3’ (to linearise *pCS-mCherry* backbone)		
Cloning, pCS-mCherry-F2: 5’-caagcataaacctggagggtTACGTAG	This study	N/A

ATCCAGACATGATAAG-3’ (to linearise *pCS-mCherry* backbone)		
Cloning, pCS-mCherry-R2: 5’-gtgaaaatgcgtttcctactCTTGTACAGCTCGTCCATG-3’ (to linearise *pCS-mCherryCAAX* backbone without *CAAX*)		
Cloning, pT2-acta1b-F: 5’-GACCCAGCTTTCTTGTAC-3’ (to linearise *pT2-actc1b:mCherry*CAAX backbone without *mCherry*)	This study	N/A
Cloning, pT2-acta1b-R: 5’-GGTGGAGCCTGCTTTTTTG-3’ (to linearise *pT2-actc1b:CherryCAAX* backbone without *mCherry*)		
Cloning, pT2-acta1b:2A-ΔCLK-F: 5’-GGCTCCGGAGCCACGAAC-3’ (to linearise *pT2-actc1b:EGFP*-2A-ΔCLK backbone without *EGFP*)	This study	N/A
Cloning, pT2-acta1b:2A-ΔCLK-R: 5’-GGTGGCGACCTTGGTCTG-3’ (to linearise *pT2-actc1b:EGFP*-2A-ΔCLK backbone without *EGFP*)		
Cloning, pT2-acta1b:LIFEACT-F: 5’-TAAGACCCAGCTTTCTTGTACAAAGTGGGGGATC-3’ (to linearise *pT2-actc1b:LIFEACT-EGFP* backbone without *EGFP*)	This study	N/A
Cloning, pT2-acta1b:LIFEACT-R: 5’-GGTGGCCACGGGGGGGTC-3’ (to linearise *pT2-actc1b:LIFEACT-EGFP* backbone without *EGFP*)		
Cloning, pT2-acta1b:mCherry-2A-F: 5’-TGAAATGGAGAGCTTGGG-3’ (to linearise *pT2-actc1b:mCherry*-2A-ΔCLK backbone without ΔCLK)	This study	N/A
Cloning, pT2-acta1b:mCherry-2A-R: 5’-AGGACCGGGGTTTTCTTC-3’ (to linearise *pT2-actc1b:mCherry*-2A-ΔCLK backbone without ΔCLK)		
Mapping, Tol2-3'-F1: 5’-TTTACTCAAGTAAGATTCTAG-3’	(7)	N/A
Mapping, Tol2-3'-F2: 5’-ACTTGTACTTTCACTTGAGTA-3’	(7)	N/A
Mapping, Tol2-5'-R1: 5’-GATTTTTAATTGTACTCAAG-3’	(7)	N/A
Mapping, Tol2-5'-R2: 5’-AAGTAAAGTAAAAATCC-3’	(7)	N/A
Oligo d(T)16 primer	Invitrogen	#N8080-128
RT-qPCR, aanat2-F: 5'-AGGACGCCATCAGTGTGTTT-3'	(50)	N/A
RT-qPCR, aanat2-R: 5'-CTGGCCCAGGAAAACAAGTA-3'		

RT-qPCR, actc1b-F: 5'-TCCCAGTGGAAGCATCAGTC-3'<br>RT-qPCR, actc1b-R: 5'-TGGGGTACTTCAGGGTCAAG-3'	(51)	N/A
RT-qPCR, atg5-F: 5'-TGGAGTATCCCACCGAAGA-3'<br>RT-qPCR, atg5-R: 5'-TGCCGTGAATCATAACCTG-3'	(52)	N/A
RT-qPCR, atg7-F: 5'-ACGGTGATGCTGTTGGTCTG-3'<br>RT-qPCR, atg7-R: 5'-TTTGTCGGTGGATTTGAAGG-3'	(52)	N/A
RT-qPCR, atg13-F: 5'-GGTTGACTTTAAACCAGCAT-3'<br>RT-qPCR, atg13-R: 5'-AAACTCTCTGTAGAATGTGC-3'	KiCqStart® SYBR® Green Primers	N/A
RT-qPCR, becn1-F: 5'-AGTCGCAGACTGAAAGTGACA-3'<br>RT-qPCR, becn1-R: 5'-TCTGGCACTCGTTCTCAGTG-3'	(53)	N/A
RT-qPCR, bmal1a-F: 5'-AGGGAAACGGCCTCTTCA-3'<br>RT-qPCR, bmal1a-R: 5'-GCGTGGCAGTGATGTTTAA-3'	(54)	N/A
RT-qPCR, clocka-F: 5'-TGGAAGAAGATGAGAAGGA-3'<br>RT-qPCR, clocka-R: 5'-TTGATGAGCACATTGAACT-3'	This study	N/A
RT-qPCR, cry1b-F: 5'-TCGCCAAGTGCATAATTGGA-3'<br>RT-qPCR, cry1b-R: 5'-GTGTGTCTCCCGAGGAAGGA-3'	(53)	N/A
RT-qPCR, cry2a-F: 5'-TCTACCAACAACTGTCCCGCTAC-3'<br>RT-qPCR, cry2a-R: 5'-GCCATCCCATTTCCATTCCC-3'	(50)	N/A
RT-qPCR, ef1a-F: 5'-AGCAGCAGCTGAGGAGTGAT-3'<br>RT-qPCR, ef1a-R: 5'-CCGCATTTGTAGATCAGATGG-3'	(2)	N/A
RT-qPCR, hsp70-F: 5'-TGGTGAAGATGAAGGAGAT-3'<br>RT-qPCR, hsp70-R: 5'-ATAGGCTGGAACTGTGAT-3'	This study	N/A
RT-qPCR, map1lc3b-F: 5'-TCCAAACAAGATCCCGGTCA-3'<br>RT-qPCR, map1lc3b-R: 5'-GACCAGCAGGAAGAAAGCCT-3'	(53)	N/A
RT-qPCR, mef2ca-F: 5'-AAAACAGACAGAAGTCATGG-3'<br>RT-qPCR, mef2ca-R: 5'-CCTCTTTGTAAACGTAACCT-3'	KiCqStart® SYBR® Green Primers	N/A
RT-qPCR, mrf4-F: 5'-CAGGAGAACCCAGATCATTC-3'<br>RT-qPCR, mrf4-R: 5'-GGACTCTGAAGACTCCAAC-3'	(55)	N/A

RT-qPCR, myf5-F: 5'-ATGGCCTCAGATGAATCAAA-3'<br>RT-qPCR, myf5-R: 5'-CATTGTGCTAGCATTTTGTG-3'	(55)	N/A
RT-qPCR, myod-F: 5'-AACATTACAGTGGAGACTCTG-3'<br>RT-qPCR, myod-R: 5'-GTCATAGCTGTTCCGTCTTC-3'	(55)	N/A
RT-qPCR, myog-F: 5'-TCAGAAACACCCACAAACGCTCAC-3'<br>RT-qPCR, myog-R: 5'-GCAGGCCCAGGGGAGACACT-3'	(55)	N/A
RT-qPCR, nr1d1-F: 5’-GAAGGCTGGAACATTTGAGGTC-’3<br>RT-qPCR, nr1d1-R: 5’-GCAGACACCAGGACGACCG-’3	(56)	N/A
RT-qPCR, nr1d2a-F: 5’-GGATGGTGTTGCTGTGTAA-’3<br>RT-qPCR, nr1d2a-R: 5’-TCTGCTGGATACTTCTTCTGA-’3	This study	N/A
RT-qPCR, p62-F: 5'-GGCTAACGTGGAGTACCTAAAG-3'<br>RT-qPCR, p62-R: 5'-TTCCCTCATGCTCAACATCG-3'	(57)	N/A
RT-qPCR, per1b-F: 5’-ATCCAGACCCCAATACAAC-’3<br>RT-qPCR, per1b-R: 5’-GGGAGACTCTGCTCCTTCT-’3	(54)	N/A
RT-qPCR, per2-F: 5’-CTTCACCACACCATACAGG-’3<br>RT-qPCR, per2-R: 5’-GTCTGACGGGGACGAGTCT-’3	(58)	N/A
RT-qPCR, psma3-F: 5'-ACGGAAGGGTATTTCAGGTTG-3'<br>RT-qPCR, psma3-R: 5'-TCATACAGCTTGGACAGTACC-3'	(59)	N/A
RT-qPCR, psma5-F: 5'-AGTAAACACTTTCTCACCAGAAG-3'<br>RT-qPCR, psma5-R: 5'-TCTCCACAGCAAGACAAACTC-3'	(59)	N/A
RT-qPCR, psmb1-F: 5'-GCTGGTGAAGATTTTGCTAT-3'<br>RT-qPCR, psmb1-R: 5'-TAGTGTCTGTCAGCTTGTAG-3'	KiCqStart® SYBR® Green Primers	N/A
RT-qPCR, psmb2-F: 5'-CAGTATAATCCAGATGAAGCAC-3'<br>RT-qPCR, psmb2-R: 5'-TGGATGTATTCTGCAAACTG-3'	KiCqStart® SYBR® Green Primers	N/A

RT-qPCR, psmb5-F: 5'-ATTTCAGCATGGTGTGATAGT-3'	KiCqStart® SYBR® Green Primers	N/A
RT-qPCR, psmb5-R: 5'-CAATCACCTTCTTCACTGTC-3'		
RT-qPCR, psmb7-F: 5'-GAGCTTCATTCTCTGTCCACC-3'	(59)	N/A
RT-qPCR, psmb7-R: 5'-GCAATCGACACCTCCAAGAAC-3'		
RT-qPCR, rorca-F: 5'-GGCTGCTCATTGATGGAA-3'	This study	N/A
RT-qPCR, rorca-R: 5'-TTCTGTGCTGTATTGACTTGT-3'		
RT-qPCR, rorcb-F: 5'-GGCTGCTCATTGATGGAA-3'	This study	N/A
RT-qPCR, rorcb-R: 5'-GTTGTAGTGTAGTGAATGAGTT-3'		
RT-qPCR, trim55b-F: 5'-CAGTATTTACCAGACGCAAA-3'	(2)	N/A
RT-qPCR, trim55b-R: 5'-ACCATCATTGCTATTCCATC-3'		
RT-qPCR, trim63a-F: 5'-TTCCGATGCCCTACTTGTCG-3'	(2)	N/A
RT-qPCR, trim63a-R: 5'-TTTCAAAGGGGGCTCAAGGG-3'		
RT-qPCR, tsc1a-F: 5'-CATTAATGGCAGTCAGGAAG-3'	(55)	N/A
RT-qPCR, tsc1a-R: 5'-GAAATGAATGAGTAAGGGCG-3'		
RT-qPCR, tsc1b-F: 5'-AAGAACATTTTCCACTCGAC-3'	(55)	N/A
RT-qPCR, tsc1b-R: 5'-AAGACACAAGGTCCAATCAT-3'		
RT-qPCR, tsc2-F: 5'-AAACTAGAGTCTCAGTCCAG-3'	(55)	N/A
RT-qPCR, tsc2-R: 5'-GCATGACCACCTGATATAGA-3'		
RT-qPCR, ulk1a-F: 5'-GAAGCTCCAAAGTCCCTCCC-3'	(53)	N/A
RT-qPCR, ulk1a-R: 5'-ACTGGCTCACTGGTGAACTG-3'		
RT-qPCR, ulk1b-F: 5'-GAAAGCTCTCCGCAGGTTCT-3'	(53)	N/A
RT-qPCR, ulk1b-R: 5'-AAAGTCACGGCTGAGATGGG-3'		
**Recombinant DNA**		
pCS-mCherry	This paper	N/A
pCS-mCherryCAAX	Roehl Lab	N/A
pCS-mCherry-p62	This paper	N/A
pCS2FA-transposase	Linker Lab	N/A
pME-LIFEACT-EGFP	Currie Lab	(60)
pT2-actc1b:EGFP-2A-ΔCLK-5xMyc	This paper	N/A
pT2-actc1b:LIFEACT-EGFP	This paper	N/A
pT2-actc1b:LIFEACT-mCherry	This paper	N/A
pT2-actc1b:mCherryCAAX	Currie Lab	(60)

pT2-actc1b:mCherry-2A-ΔCLK-5xMyc	This paper	N/A
pT2-actc1b:mCherry-2A-nr1d1(FL)-6xHis	This paper	N/A
pT2-actc1b:mCherry-2A-nr1d1(Mut)-6xHis	This paper	N/A
pT2-actc1b:mCherry-2A-rorca-6xHis	This paper	N/A
pT2-actc1b:mCherry-2A-rorcb-6xHis	This paper	N/A
pT2-UAS:EGFP-2A-ΔCLK-5xMyc	Gothilf Lab	(6)
**Software and algorithms**		
Algorithm (R), CircaCompare	(26)	https://github.com/RWParsons/circacompare/
Algorithm (R), MetaCycle	(25)	https://github.com/gangwug/MetaCycle
Algorithm (R), Pcal	(27)	https://github.com/cran/pcal
Algorithm (R), pracma	Hans W. Borchers	https://github.com/cran/pracma
ApE (version 3.1.4)	(26, 61)	https://jorgensen.biology.utah.edu/wayned/ape/
BioRender	BioRender	https://www.biorender.com/
BLAST	NIH	https://blast.ncbi.nlm.nih.gov/
CorelDraw X7	CorelDraw	https://www.coreldraw.com/
ENSEMBL Zebrafish	ENSEMBL	https://www.ensembl.org/Danio_rerio/
Fiji/ImageJ (version 1.53t)	NIH	https://fiji.sc/
FIMO (version 5.5.5)	MEME Suite	https://meme-suite.org/
GraphPad Prism (version 9.4.110.2.0)	GraphPad	https://www.graphpad.com/
idTracker	idTracker	https://www.idtracker.es/
Image Lab™ (version 6.0.1 Build 34)	Bio-rad	https://www.bio-rad.com/
MATLAB (version R2019b Build 9.7.0.1190202).	MathWorks	https://www.mathworks.com/
Microsoft® Excel® for Microsoft 365 MSO (version 2202 Build 16.0.14931.20764), 64-bit	Microsoft	https://www.microsoft.com/en-us/microsoft-365/excel/
NEBuilder® Assembly Tool	NEB	https://nebuilder.neb.com/
OligoArchitect™	Sigma-Aldrich	http://www.oligoarchitect.com/
Primer-BLAST	NIH	https://www.ncbi.nlm.nih.gov/tools/primer-blast/
R (version 4.1.2)	The R Foundation	https://www.r-project.org/
RStudio (version 2021.09.1 Build 372)	Rstudio, PBC	https://posit.co/
Script (MATLAB), idTracker analysis	Michael Green	https://groups.google.com/g/idtracker-users/
SnapGene (version 7.2.0)	SnapGene	https://www.snapgene.com/
ZEN 2.3 SP1 FP1 (black), 64-bit	Carl Zeiss	https://www.zeiss.co.uk/
Zebrafish Information Network (ZFIN)	Zebrafish Information Network (ZFIN)	https://zfin.org/

ZebraZoom (version 1.33.13)	(21)	https://github.com/oliviermirat/ZebraZoom/
ZEN 2.3 SP1 FP1 (black), 64-bit	Carl Zeiss	https://www.zeiss.co.uk/
**Other**		
Camera (app version 8.7.250.494820638.44)	Google	Pixel 6
CellStar™ 96-Well, Cell Culture-Treated, Flat-Bottom Microplate, White, Sterile	Greiner Bio-One	#655083
Amersham™ Hybond™ P PVDF Membrane, 0.45 µm	GE Healthcare	#GE10600023
CellStar™ 96-Well, Cell Culture-Treated, Flat-Bottom Microplate, White, Sterile	Greiner Bio-One	#655083
ChemiDoc™ Imaging System	Bio-rad	N/A
Depression slide	Sigma-Aldrich	#BR475505
Gel Doc™ XR+ Imaing System	Bio-rad	N/A
LED Strips	Ustellar	N/A
Leica MZ16F microscope with iDS camera	Leica/ iDS	N/A
LSM 5 Exciter microscope	Carl Zeiss	N/A
LSM 880 with Airyscan microscope	Carl Zeiss	N/A
MicroAmp™ Optical 384-Well Reaction Plate with Barcode	Applied Biosystems	#4309849
MicroAmp™ Optical Adhesive Film	Applied Biosystems	#4311971
Mini-PROTEAN® TGX™ Precast Gels	Bio-rad	#456-1094 and #456-1096
Mithras LB 940 plate reader	Berthold Technologies	N/A
Platinum (Pt) wire, ø0.4 mm	Goodfellow	#PT00-WR-000142
QuantStudio™ 7 Flex Real-Time PCR System	Thermo Fisher Scientific	N/A
Soniprep 50 Sonicator	MSE	N/A
Spectrophotometer	DeNovix	#DS-11
Supported Nitrocellulose Membrane, 0.45 µm	Bio-rad	#1620094
Timer Switch	Status	#TE-10
Transilluminator	UVP	#TM-20E
ViiA™ 7 Real-Time PCR System	Thermo Fisher Scientific	N/A
Weighing Balance	Ohaus	#YA102

Table S3. False Positive Risk (FPR) estimates

Figures	Comparisons	*p*-values	
		Uncalibrated	Calibrated (FPR estimates)
1d	active_ctrl_L_vs_active_ctrl_D	4.00E-04	0.008435
1d	inactive_ctrl_L_vs_inactive_ctrl_D	5.00E-05	0.001344
1d	active_ctrl_L_vs_inactive_ctrl_L	3.00E-04	0.006572
1d	active_ctrl_D_vs_inactive_ctrl_D	4.00E-05	0.0011
1d	active_ctrl_D_vs_active_mdclk_D	6.00E-05	0.001583
1d	active_mdclk_D_vs_inactive_mdclk_D	0.013	0.133047
1d	inactive_ctrl_D_vs_inactive_mdclk_D	4.00E-08	1.85E-06
1d	anova_time	3.00E-04	0.006572
1d	anova_genotype	5.00E-07	1.97E-05
1d	anova_activity	4.00E-11	2.60E-09
1d	anova_time_genotype	3.00E-07	1.22E-05
1e	ctrl_3dpf_vs_mdclk_3dpf	0.02	0.17538
1e	ctrl_4dpf_vs_mdclk_4dpf	0.004	0.056635
1e	ctrl_5dpf_vs_mdclk_5dpf	7.00E-06	0.000226
1e	anova_3dpf_vs_4dpf_stage	5.00E-04	0.010225
1e	anova_3dpf_vs_4dpf_stage_genotype	5.00E-04	0.010225
1e	brown_forsythe_test_5dpf_vs_8dpf_vs_14dpf	2.00E-23	2.84E-21
1f_left	ctrl_5mpf_vs_ctrl_12mpf	2.00E-04	0.004609
1f_left	ctrl_5mpf_vs_ctrl_24mpf	2.00E-04	0.004609
1f_left	mdclk_5mpf_vs_mdclk_12mpf	2.00E-06	7.13E-05
1f_left	mdclk_5mpf_vs_mdclk_24mpf	0.008	0.095021
1f_left	ctrl_24mpf_vs_mdclk_24mpf	0.02	0.17538
1f_left	anova_age	8.00E-10	4.56E-08
1f_left	anova_genotype	0.014	0.139747
1f_right	ctrl_vs_mdclk	4.00E-05	0.0011
2b	ctrl_murf1a	0.006	0.077014
2b	ctrl_murf2b	4.00E-04	0.008435
2c	bmal_ct3_vs_bmal_ct15	0.039	0.255912
2c	bmal_ct3_vs_bmal_ct21	0.02	0.17538
2c	bmal_anova_time	0.008	0.095021
2c	murf_ct3_vs_murf_ct21	0.02	0.17538
2c	murf_anova_lay	0.044	0.271982
2c	murf_anova_time	0.015	0.146204
2d	dmso_ct24_vs_btz_ct24	0.004	0.056635
2d	btz_ct12_vs_btz_ct24	0.025	0.200438
2d	btz_ct24_vs_btz_myomed_ct24	0.004	0.056635
2d	anova_treatment	0.001	0.018431
2e	active_dmso_L_vs_active_dmso_D	0.02	0.17538
2e	inactive_dmso_L_vs_inactive_dmso_D	0.008	0.095021
2e	active_dmso_L_vs_inactive_dmso_L	6.00E-06	0.000196
2e	active_myomed_L_vs_inactive_myomed_L	0.005	0.067174
2e	active_dmso_D_vs_inactive_dmso_D	6.00E-07	2.34E-05
2e	active_dmso_D_vs_active_myomed_D	0.006	0.077014

2e	active_myomed_D_vs_inactive_myomed_D	1.00E-04	0.002497
2e	inactive_myomed_D_vs_inactive_myomed_D	7.00E-05	0.001817
2f	dmso_ct14_vs_dmso_ct16	0.008	0.095021
2f	dmso_ct14_vs_dmso_ct20	4.00E-04	0.008435
2f	dmso_ct14_vs_dmso_ct24	9.00E-07	3.41E-05
2f	dmso_ct14_vs_mg132_ct24	0.046	0.277986
2f	dmso_ct24_vs_mg132_ct24	0.032	0.230416
2f	dmso_ct24_vs_baf_ct24	0.002	0.032682
2f	anova_treatment	2.00E-06	7.13E-05
3a	ctrl_ct3_vs_ctrl_ct15	3.00E-05	0.000849
3a	ctrl_ct3_vs_ctrl_ct21	3.00E-10	1.79E-08
3a	ctrl_ct9_vs_ctrl_ct15	0.029	0.218197
3a	ctrl_ct9_vs_ctrl_ct21	2.00E-07	8.39E-06
3a	ctrl_ct15_vs_ctrl_ct21	7.00E-04	0.013634
3a	mdclk_ct3_vs_mdclk_ct15	6.00E-04	0.011955
3a	mdclk_ct3_vs_mdclk_ct21	2.00E-04	0.004609
3a	ctrl_ct21_vs_mdclk_ct21	0.003	0.04523
3a	anova_time	1.00E-10	6.26E-09
3a	anova_time_genotype	0.003	0.04523
3b	ctrl_dmso_ct15_vs_ctrl_btz_ct15	0.038	0.252499
3b	ctrl_btz_ct3_vs_ctrl_btz_ct15	0.032	0.230416
3b	anova_treatment	0.033	0.234303
3b	anova_time_genotype	0.012	0.126081
3b	anova_time_treatment	0.044	0.271982
3c	ctrl_dmso_L_vs_ctrl_dmso_D	0.002	0.032682
3c	ctrl_dmso_D_vs_ctrl_btz_D	5.00E-04	0.010225
3c	ctrl_dmso_D_vs_ctrl_baf_D	0.041	0.262532
3c	ctrl_dmso_D_vs_mdclk_dmso_D	0.008	0.095021
3d	ctrl_dmso_vs_ctrl_baf	0.012	0.126081
3d	ctrl_dmso_vs_mdclk_dmso	0.038	0.252499
3d	anova_genotype	0.04	0.259255
3d	anova_treatment_genotype	0.031	0.226439
4b	ctrl_ulk1a	1.00E-04	0.002497
4b	ctrl_ulk1b	9.00E-05	0.002274
4c	ct9_vs_ct21	0.007	0.086269
4c	anova_time	0.007	0.086269
4d	p62_dmso_ct24_vs_p62_baf_ct24	0.008	0.095021
4d	p62_baf_ct12_vs_p62_baf_ct24	0.003	0.04523
4d	p62_baf_ct24_vs_p62_baf_mrt_ct24	2.00E-04	0.004609
4d	p62_anova_treatment	2.00E-04	0.004609
4d	lc3_dmso_ct12_vs_lc3_baf_ct12	0.005	0.067174
4d	lc3_dmso_ct24_vs_lc3_baf_ct24	3.00E-04	0.006572
4d	lc3_baf_ct24_vs_lc3_baf_mrt_ct24	0.045	0.275012
4d	lc3_anova_treatment	7.00E-05	0.001817
4e	active_dmso_L_vs_active_dmso_D	2.00E-04	0.004609
4e	inactive_dmso_L_vs_inactive_dmso_D	1.00E-04	0.002497

4e	active_dmso_L_vs_inactive_dmso_L	1.00E-05	0.000313
4e	active_mrt_L_vs_inactive_mrt_L	1.00E-05	0.000313
4e	active_dmso_D_vs_inactive_dmso_D	5.00E-06	0.000166
4e	active_dmso_D_vs_active_mrt_D	3.00E-04	0.006572
4e	active_mrt_D_vs_inactive_mrt_D	6.00E-06	0.000196
4e	inactive_mrt_D_vs_inactive_mrt_D	2.00E-04	0.004609
4f	active_dmso_L_vs_active_dmso_D	0.002	0.032682
4f	inactive_dmso_L_vs_inactive_dmso_D	8.00E-04	0.01527
4f	active_dmso_L_vs_inactive_dmso_L	3.00E-05	0.000849
4f	active_baf_L_vs_inactive_baf_L	0.03	0.222367
4f	active_dmso_D_vs_inactive_dmso_D	6.00E-06	0.000196
4f	active_dmso_D_vs_active_baf_D	2.00E-04	0.004609
4f	active_baf_D_vs_inactive_baf_D	0.001	0.018431
4f	inactive_baf_D_vs_inactive_baf_D	1.00E-05	0.000313
5a	ulk1p_ulk1_ratio_ctrl_ct3_vs_ulk1p_ulk1_ratio_ctrl_ct21	4.00E-05	0.0011
5a	ulk1p_ulk1_ratio_ctrl_ct9_vs_ulk1p_ulk1_ratio_ctrl_ct21	0.004	0.056635
5a	ulk1p_ulk1_ratio_ctrl_ct15_vs_ulk1p_ulk1_ratio_ctrl_ct21	0.042	0.265743
5a	ulk1p_ulk1_ratio_ctrl_ct21_vs_ulk1p_ulk1_ratio_mdclk_ct21	0.022	0.185832
5a	ulk1p_ulk1_ratio_anova_time	4.00E-04	0.008435
5a	ulk1p_ulk1_ratio_anova_time_genotype	0.041	0.262532
5a	eif4ebpp_eif4ebp_ratio_ctrl_ct3_vs_eif4ebpp_eif4ebp_ratio_ctrl_ct9	0.006	0.077014
5a	eif4ebpp_eif4ebp_ratio_ctrl_ct3_vs_eif4ebpp_eif4ebp_ratio_ctrl_ct15	0.016	0.152434
5a	eif4ebpp_eif4ebp_ratio_ctrl_ct21_vs_eif4ebpp_eif4ebp_ratio_mdclk_ct21	0.034	0.238103
5a	eif4ebpp_eif4ebp_ratio_anova_time	0.012	0.126081
5a	eif4ebpp_eif4ebp_ratio_anova_genotype	0.043	0.268893
5b	ctrl_dmso_vs_ctrl_baf	0.004	0.056635
5b	mdclk_dmso_vs_mdclk_baf	0.011	0.118826
5b	ctrl_dmso_vs_mdclk_dmso	0.003	0.04523
5b	ctrl_baf_vs_mdclk_baf	2.00E-04	0.004609
5b	anova_treatment	0.003	0.04523
5b	anova_genotype	1.00E-04	0.002497
5b	anova_treatment_genotype	4.00E-04	0.008435
5c	ctrl_dmso_vs_ctrl_baf	3.00E-07	1.22E-05
5c	ctrl_dmso_vs_mdclk_dmso	0.005	0.067174
5c	anova_treatment	3.00E-06	0.000104
5c	anova_treatment_genotype	0.001	0.018431
6a	dmso_L_vs_dmso_D	0.02	0.17538
6a	sr9011_ct0to12_L_vs_sr9011_ct0to12_D	0.018	0.164276
6a	sr8278_ct0to12_L_vs_sr8278_ct0to12_D	1.00E-04	0.002497
6a	sr8278_ct12to24_L_vs_sr8278_ct12to24_D	1.00E-04	0.002497
6a	dmso_D_vs_sr9011_ct12to24_D	0.02	0.17538
6a	sr9011_ct0to12_D_vs_sr9011_ct12to24_D	7.00E-06	0.000226
6b	nr1d1_MUT_neighbour_mcherry_neg_vs_nr1d1_FL	0.013	0.133047
6b	nr1d1_MUT_vs_nr1d1_FL	2.00E-04	0.004609
6b	nr1d1_FL_mcherry_neighbour_neg_vs_nr1d1_FL	0.002	0.032682
6b	anova_genotype	0.029	0.218197

6b	anova_treatment_genotype	0.002	0.032682
6c	mcherry_neg_neighbour_own_vs_mcherry_pos_rorca	3.00E-09	1.60E-07
6c	mcherry_neg_neighbour_own_vs_mcherry_pos_rorcb	3.00E-09	1.60E-07
6c	anova_treatment	6.00E-17	6.09E-15
6c	anova_genotype	0.012	0.126081
6d_left	dmso_vs_baf	5.00E-11	3.22E-09
6d_left	baf_vs_baf_sr9011	3.00E-04	0.006572
6d_left	anova_treatment	1.00E-10	6.26E-09
6d_right	dmso_vs_baf	2.00E-08	9.64E-07
6d_right	baf_vs_baf_sr98278	0.013	0.133047
6d_right	baf_vs_baf_rapa	0.005	0.067174
6d_right	anova_treatment	7.00E-15	6.20E-13
6e_left	ulk1p_dmso_vs_ulk1p_sr9011	0.011	0.118826
6e_left	ulk1p_dmso_vs_ulk1p_sr8278	0.032	0.230416
6e_left	ulk1p_sr9011_vs_ulk1p_sr8278	7.00E-06	0.000226
6e_left	ulk1p_anova_lay	1.00E-05	0.000313
6e_left	ulk1p_anova_treatment	1.00E-05	0.000313
6e_right	rps6p_dmso_vs_rps6p_sr9011	9.00E-09	4.53E-07
6e_right	rps6p_sr9011_vs_rps6p_sr8278	1.00E-06	3.76E-05
6e_right	rps6p_anova_lay	5.00E-04	0.010225
6e_right	rps6p_anova_treatment	1.00E-09	5.63E-08
6f_left	ulk1p_nr1d1_MUT_vs_nr1d1_FL	2.00E-05	0.000588
6f_right	rps6p_nr1d1_MUT_vs_nr1d1_FL	1.00E-04	0.002497
6f_right	anova_lay	1.00E-10	6.26E-09
6g	ctrl_vs_mdclk	0.001	0.018431
6h	ctrl_dmso_L_vs_ctrl_dmso_D	3.00E-06	0.000104
6h	ctrl_dmso_L_vs_ctrl_rapa_L	2.00E-09	1.09E-07
6h	ctrl_rapa_L_vs_mdclk_rapa_L	2.00E-05	0.000588
6h	ctrl_dmso_D_vs_mdclk_dmso_D	4.00E-10	2.35E-08
6h	mdclk_dmso_D_vs_mdclk_rapa_D	0.002	0.032682
6i	ctrl_dmso_L_vs_ctrl_dmso_D	0.008	0.095021
6i	ctrl_sr9011_ct0to12_L_vs_ctrl_sr9011_ct12to24_D	3.00E-04	0.006572
6i	mdclk_sr8278_ct0to12_L_vs_mdclk_sr8278_ct12to24_D	4.00E-04	0.008435
6i	ctrl_dmso_D_vs_ctrl_sr9011_ct12to24_D	2.00E-04	0.004609
6i	ctrl_dmso_D_vs_mdclk_dmso_D	3.00E-04	0.006572
6i	mdclk_dmso_D_vs_mdclk_sr8278_ct12to24_D	0.006	0.077014
s1d	bmal1a_ctrl	0.001	0.018431
s1d	bmal1a_mdclk	0.002	0.032682
s1d	clocka_ctrl	0.001	0.018431
s1d	per1b_ctrl	2.00E-05	0.000588
s1d	rorca_ctrl	3.00E-07	1.22E-05
s1d	rorca_mdclk	1.00E-05	0.000313
s1d	nr1d1_ctrl	5.00E-05	0.001344
s1d	nr1d1_mdclk	0.009	0.103333
s1d	aanat2_ctrl	1.00E-07	4.38E-06
s1d	aanat2_mdclk	4.00E-09	2.10E-07

s1d	myf5_ctrl	0.011	0.118826
s1d	mef2ca_ctrl	0.011	0.118826
s1d	myod_ctrl	0.018	0.164276
s1d	mrf4_ctrl	0.006	0.077014
s1d	mrf4_mdclk	0.041	0.262532
s1d	mrf4_ctrl_vs_mdclk_mesor	0.011	0.118826
s2b	clocka_ctrl_ct3_vs_clocka_mdclk_ct3	9.00E-10	5.10E-08
s2b	clocka_ctrl_ct9_vs_clocka_mdclk_ct9	2.00E-10	1.21E-08
s2b	clocka_ctrl_ct15_vs_clocka_mdclk_ct15	8.00E-10	4.56E-08
s2b	clocka_ctrl_ct21_vs_clocka_mdclk_ct21	3.00E-09	1.60E-07
s2b	clocka_ctrl_vs_clocka_mdclk_median	1.00E-05	0.000313
s2b	clocka_anova_genotype	2.00E-14	1.71E-12
s2b	bmal1a_ctrl_ct3_vs_bmal1a_ctrl_ct21	8.00E-04	0.015270
s2b	bmal1a_ctrl_ct9_vs_bmal1a_ctrl_ct21	0.006	0.077014
s2b	bmal1a_mdclk_ct3_vs_bmal1a_mdclk_ct9	0.013	0.133047
s2b	bmal1a_mdclk_ct9_vs_bmal1a_mdclk_ct21	8.00E-05	0.002047
s2b	bmal1a_mdclk_ct15_vs_bmal1a_mdclk_ct21	0.008	0.095021
s2b	bmal1a_ctrl_ct9_vs_bmal1a_mdclk_ct9	0.019	0.169914
s2b	bmal1a_anova_time	9.00E-06	0.000284
s2b	per1b_ctrl_ct3_vs_per1b_ctrl_ct15	0.037	0.249015
s2b	per1b_ctrl_ct3_vs_per1b_ctrl_ct21	0.014	0.139747
s2b	per1b_ctrl_ct3_vs_per1b_mdclk_ct3	0.003	0.04523
s2b	per1b_ctrl_vs_mdclk_per1b_median	0.004	0.056635
s2b	per1b_anova_time	0.013	0.133047
s2b	per1b_anova_genotype	0.004	0.056635
s2b	per2_ctrl_ct3_vs_per2_ctrl_ct9	8.00E-09	4.05E-07
s2b	per2_ctrl_ct3_vs_per2_ctrl_ct15	2.00E-09	1.09E-07
s2b	per2_ctrl_ct3_vs_per2_ctrl_ct21	2.00E-08	9.64E-07
s2b	per2_mdclk_ct3_vs_per2_mdclk_ct9	2.00E-06	7.13E-05
s2b	per2_mdclk_ct3_vs_per2_mdclk_ct15	2.00E-07	8.39E-06
s2b	per2_mdclk_ct3_vs_per2_mdclk_ct21	6.00E-07	2.34E-05
s2b	per2_ctrl_ct3_vs_per2_mdclk_ct3	2.00E-04	0.004609
s2b	per2_ctrl_ct21_vs_per2_mdclk_ct21	0.049	0.286586
s2b	per2_ctrl_vs_mdclk_per1b_median	0.011	0.118826
s2b	per2_anova_time	4.00E-11	2.60E-09
s2b	per2_anova_genotype	3.00E-04	0.006572
s2b	cry1b_ctrl_ct3_vs_cry1b_ctrl_ct9	4.00E-07	1.60E-05
s2b	cry1b_ctrl_ct3_vs_cry1b_ctrl_ct15	4.00E-11	2.60E-09
s2b	cry1b_ctrl_ct3_vs_cry1b_ctrl_ct21	3.00E-11	1.98E-09
s2b	cry1b_ctrl_ct9_vs_cry1b_ctrl_ct15	3.00E-06	0.000104
s2b	cry1b_ctrl_ct9_vs_cry1b_ctrl_ct21	2.00E-06	7.13E-05
s2b	cry1b_mdclk_ct3_vs_cry1b_mdclk_ct9	8.00E-06	0.000255
s2b	cry1b_mdclk_ct3_vs_cry1b_mdclk_ct15	1.00E-08	5.01E-07
s2b	cry1b_mdclk_ct3_vs_cry1b_mdclk_ct21	3.00E-09	1.60E-07
s2b	cry1b_mdclk_ct9_vs_cry1b_mdclk_ct15	0.002	0.032682
s2b	cry1b_mdclk_ct9_vs_cry1b_mdclk_ct21	1.00E-04	0.002497

s2b	cry1b_ctrl_ct3_vs_cry1b_mdclk_ct3	8.00E-05	0.002047
s2b	cry1b_ctrl_ct9_vs_cry1b_mdclk_ct9	0.005	0.067174
s2b	cry1b_ctrl_vs_cry1b_mdclk_median	0.034	0.238103
s2b	cry1b_anova_time	6.00E-13	4.59E-11
s2b	cry1b_anova_genotype	3.00E-04	0.006572
s2b	cry1b_anova_time_genotype	0.006	0.077014
s2b	cry2a_ctrl_ct3_vs_cry2a_ctrl_ct9	1.00E-06	3.76E-05
s2b	cry2a_ctrl_ct3_vs_cry2a_ctrl_ct15	7.00E-07	2.70E-05
s2b	cry2a_ctrl_ct3_vs_cry2a_ctrl_ct21	0.038	0.252499
s2b	cry2a_ctrl_ct9_vs_cry2a_ctrl_ct21	2.00E-04	0.004609
s2b	cry2a_ctrl_ct15_vs_cry2a_ctrl_ct21	1.00E-04	0.002497
s2b	cry2a_mdclk_ct3_vs_cry2a_mdclk_ct9	1.00E-06	3.76E-05
s2b	cry2a_mdclk_ct3_vs_cry2a_mdclk_ct15	5.00E-04	0.010225
s2b	cry2a_mdclk_ct9_vs_cry2a_mdclk_ct15	0.02	0.17538
s2b	cry2a_mdclk_ct9_vs_cry2a_mdclk_ct21	9.00E-05	0.002274
s2b	cry2a_ctrl_ct15_vs_cry2a_mdclk_ct15	3.00E-05	0.000849
s2b	cry2a_ctrl_ct21_vs_cry2a_mdclk_ct21	0.026	0.20505
s2b	cry2a_ctrl_vs_cry2a_mdclk_median	0.004	0.056635
s2b	cry2a_anova_time	5.00E-09	2.60E-07
s2b	cry2a_anova_genotype	2.00E-05	0.000588
s2b	rorca_ctrl_ct3_vs_rorca_ctrl_ct9	3.00E-05	0.000849
s2b	rorca_ctrl_ct3_vs_rorca_ctrl_ct15	0.044	0.271982
s2b	rorca_ctrl_ct9_vs_rorca_ctrl_ct15	0.01	0.111254
s2b	rorca_ctrl_ct9_vs_rorca_ctrl_ct21	1.00E-06	3.76E-05
s2b	rorca_ctrl_ct15_vs_rorca_ctrl_ct21	0.001	0.018431
s2b	rorca_mdclk_ct9_vs_rorca_mdclk_ct21	0.01	0.111254
s2b	rorca_ctrl_ct9_vs_rorca_mdclk_ct9	4.00E-04	0.008435
s2b	rorca_ctrl_ct15_vs_rorca_mdclk_ct15	0.027	0.209543
s2b	rorca_anova_time	2.00E-06	7.13E-05
s2b	rorca_anova_genotype	0.006	0.077014
s2b	rorca_anova_time_genotype	0.009	0.103333
s2b	rorcb_anova_time	0.023	0.190836
s2b	rorcb_ctrl_ct9_vs_rorcb_ctrl_ct21	0.006	0.077014
s2b	rorcb_ctrl_ct9_vs_rorcb_mdclk_ct9	0.027	0.209543
s2b	nr1d1_ctrl_ct3_vs_nr1d1_ctrl_ct21	1.00E-06	3.76E-05
s2b	nr1d1_ctrl_ct9_vs_nr1d1_ctrl_ct21	8.00E-08	3.55E-06
s2b	nr1d1_ctrl_ct15_vs_nr1d1_ctrl_ct21	1.00E-07	4.38E-06
s2b	nr1d1_mdclk_ct3_vs_nr1d1_mdclk_ct21	0.005	0.067174
s2b	nr1d1_mdclk_ct9_vs_nr1d1_mdclk_ct21	8.00E-04	0.01527
s2b	nr1d1_mdclk_ct15_vs_nr1d1_mdclk_ct21	1.00E-04	0.002497
s2b	nr1d1_ctrl_ct9_vs_nr1d1_mdclk_ct9	0.027	0.209543
s2b	nr1d1_ctrl_ct21_vs_nr1d1_mdclk_ct21	0.007	0.086269
s2b	nr1d1_ctrl_vs_nr1d1_mdclk_median	0.013	0.133047
s2b	nr1d1_anova_time	8.00E-09	4.05E-07
s2b	nr1d1_anova_time_genotype	0.006	0.077014
s2b	nr1d2a_ctrl_ct3_vs_nr1d2a_ctrl_ct9	0.003	0.04523

s2b	nr1d2a_ctrl_ct3_vs_nr1d2a_ctrl_ct21	3.00E-06	0.000104
s2b	nr1d2a_ctrl_ct9_vs_nr1d2a_ctrl_ct15	0.004	0.056635
s2b	nr1d2a_ctrl_ct9_vs_nr1d2a_ctrl_ct21	8.00E-09	4.05E-07
s2b	nr1d2a_ctrl_ct15_vs_nr1d2a_ctrl_ct21	3.00E-06	0.000104
s2b	nr1d2a_mdclk_ct3_vs_nr1d2a_mdclk_ct9	0.004	0.056635
s2b	nr1d2a_mdclk_ct3_vs_nr1d2a_mdclk_ct21	1.00E-04	0.002497
s2b	nr1d2a_mdclk_ct9_vs_nr1d2a_mdclk_ct15	2.00E-04	0.004609
s2b	nr1d2a_mdclk_ct9_vs_nr1d2a_mdclk_ct21	1.00E-07	4.38E-06
s2b	nr1d2a_mdclk_ct15_vs_nr1d2a_mdclk_ct21	0.002	0.032682
s2b	nr1d2a_ctrl_ct15_vs_nr1d2a_mdclk_ct15	0.028	0.213924
s2b	nr1d2a_ctrl_vs_nr1d2a_mdclk_median	0.049	0.286586
s2b	nr1d2a_anova_time	4.00E-10	2.35E-08
s2c	clocka_ctrl_ct6_vs_clocka_mdclk_ct6	3.00E-05	0.000849
s2c	clocka_ctrl_ct18_vs_clocka_mdclk_ct18	2.00E-05	0.000588
s2c	clocka_anova_genotype	9.00E-07	3.41E-05
s2c	bmal1a_ctrl_ct6_vs_bmal1a_ctrl_ct18	0.029	0.218197
s2c	bmal1a_ctrl_ct6_vs_bmal1a_mdclk_ct6	6.00E-04	0.011955
s2c	bmal1a_ctrl_ct18_vs_bmal1a_mdclk_ct18	0.045	0.275012
s2c	bmal1a_anova_genotype	0.002	0.032682
s2c	bmal1a_anova_time_genotype	0.003	0.04523
s2c	per1b_ctrl_ct6_vs_per1b_ctrl_ct18	0.042	0.265743
s2c	per1b_anova_time_genotype	0.047	0.280905
s2c	nr1d1_ctrl_ct6_vs_nr1d1_ctrl_ct18	0.001	0.018431
s2c	nr1d1_ctrl_ct18_vs_nr1d1_mdclk_ct18	0.022	0.185832
s2c	nr1d1_anova_time	6.00E-04	0.011955
s2c	nr1d1_anova_genotype	0.047	0.280905
s2c	nr1d1_anova_time_genotype	0.049	0.286586
s3b	clocka_ctrl_vs_clocka_mdclk	0.001	0.018431
s3b	per1b_ctrl_vs_per1b_mdclk	0.01	0.111254
s3c	ctrl_L_vs_ctrl_D	0.009	0.103333
s3d	active_ctrl_L_vs_active_ctrl_D	0.004	0.056635
s3d	active_mdclk_L_vs_active_mdclk_D	0.05	0.28935
s3d	inactive_ctrl_L_vs_inactive_ctrl_D	7.00E-05	0.001817
s3d	active_ctrl_L_vs_inactive_ctrl_L	6.00E-06	0.000196
s3d	active_ctrl_D_vs_inactive_ctrl_D	2.00E-08	9.64E-07
s3d	active_ctrl_D_vs_active_mdclk_D	5.00E-06	0.000166
s3d	active_mdclk_D_vs_inactive_mdclk_D	2.00E-04	0.004609
s3d	inactive_ctrl_D_vs_inactive_mdclk_D	2.00E-10	1.21E-08
s3d	anova_time	0.028	0.213924
s3d	anova_genotype	2.00E-08	9.64E-07
s3d	anova_activity	1.00E-15	9.39E-14
s3d	anova_time_genotype	1.00E-08	5.01E-07
s3d	anova_genotype_activity	0.019	0.169914
s3f	ctrl_3dpf_vs_ctrl_4dpf	3.00E-11	1.98E-09
s3f	egfp_3dpf_vs_egfp_4dpf	6.00E-11	3.84E-09
s3f	anova_stage	5.00E-14	4.16E-12

s3g	ctrl_L_vs_ctrl_D	0.02	0.17538
s3g	egfp_L_vs_egfp_D	0.026	0.20505
s4_left	ctrl_5mpf_vs_ctrl_12mpf	4.00E-17	4.11E-15
s4_left	ctrl_5mpf_vs_ctrl_24mpf	5.00E-34	1.04E-31
s4_left	ctrl_12mpf_vs_ctrl_24mpf	4.00E-12	2.85E-10
s4_left	mdclk_5mpf_vs_mdclk_12mpf	5.00E-16	4.79E-14
s4_left	mdclk_5mpf_vs_mdclk_24mpf	2.00E-12	1.46E-10
s4_left	ctrl_24mpf_vs_mdclk_24mpf	5.00E-15	4.48E-13
s4_left	anova_age	9.00E-37	2.03E-34
s4_left	anova_genotype	3.00E-08	1.41E-06
s4_left	anova_age_genotype	2.00E-10	1.21E-08
s4_middle	ctrl_5mpf_vs_ctrl_12mpf	1.00E-20	1.25E-18
s4_middle	ctrl_5mpf_vs_ctrl_24mpf	4.00E-36	8.86E-34
s4_middle	ctrl_12mpf_vs_ctrl_24mpf	3.00E-11	1.98E-09
s4_middle	mdclk_5mpf_vs_mdclk_12mpf	5.00E-17	5.10E-15
s4_middle	mdclk_5mpf_vs_mdclk_24mpf	8.00E-17	8.06E-15
s4_middle	ctrl_24mpf_vs_mdclk_24mpf	2.00E-10	1.21E-08
s4_middle	anova_age	7.00E-41	1.76E-38
s4_middle	anova_genotype	2.00E-04	0.004609
s4_middle	anova_age_genotype	4.00E-08	1.85E-06
s4_right	ctrl_5mpf_vs_ctrl_12mpf	8.00E-14	6.56E-12
s4_right	ctrl_5mpf_vs_ctrl_24mpf	2.00E-21	2.59E-19
s4_right	ctrl_12mpf_vs_ctrl_24mpf	0.001	0.018431
s4_right	mdclk_5mpf_vs_mdclk_12mpf	2.00E-14	1.71E-12
s4_right	mdclk_5mpf_vs_mdclk_24mpf	3.00E-09	1.60E-07
s4_right	ctrl_24mpf_vs_mdclk_24mpf	8.00E-08	3.55E-06
s4_right	anova_age	5.00E-28	8.54E-26
s4_right	anova_genotype	3.00E-05	0.000849
s4_right	anova_age_genotype	2.00E-04	0.004609
s5a_top	bmal_ct15_vs_bmal_ct9	0.037	0.249015
s5a_top	bmal1_anova_time	0.031	0.226439
s5a_bottom	murf_ct15_vs_murf_ct21	0.007	0.086269
s5a_bottom	murf_ct15_vs_murf_ct3	0.002	0.032682
s5a_bottom	murf_ct15_vs_murf_ct9	7.00E-04	0.013634
s5a_bottom	murf_anova_lay	0.003	0.04523
s5a_bottom	murf_anova_time	6.00E-04	0.011955
s5b_left	dmso_vs_tricaine	1.00E-06	3.76E-05
s5b_left	dmso_vs_terfenadine	0.001	0.018431
s5b_left	anova_treatment	3.00E-07	1.22E-05
s5b_middle	dmso_vs_tricaine	6.00E-07	2.34E-05
s5b_middle	dmso_vs_terfenadine	0.003	0.045230
s5b_middle	anova_treatment	2.00E-07	8.39E-06
s5c_top_left	active_dmso_vs_inactive_dmso	3.00E-04	0.006572
s5c_top_left	active_myomed_vs_inactive_myomed	8.00E-05	0.002047
s5c_top_left	anova_activity	2.00E-07	8.39E-06
s5c_top_left	anova_treatment	0.029	0.218197

s5c_bottom_left	active_dmso_vs_inactive_dmso	2.00E-08	9.64E-07
s5c_bottom_left	active_btz_mrt_vs_inactive_btz_mrt	4.00E-06	0.000135
s5c_bottom_left	anova_activity	2.00E-11	1.34E-09
s5c_top_right	active_fishwater_vs_inactive_fishwater	2.00E-07	8.39E-06
s5c_top_right	active_fishwater_vs_active_mrt	0.012	0.126081
s5c_top_right	active_mrt_vs_inactive_mrt	1.00E-07	4.38E-06
s5c_top_right	inactive_fishwater_vs_inactive_mrt	0.028	0.213924
s5c_top_right	anova_activity	8.00E-12	5.56E-10
s5c_top_right	anova_treatment	3.00E-04	0.006572
s5c_bottom_ right	active_dmso_vs_inactive_dmso	7.00E-05	0.001817
s5c_bottom_ right	active_baf_vs_inactive_baf	0.013	0.133047
s5c_bottom_ right	anova_activity	5.00E-06	0.000166
s5d	dmso_ct14_vs_dmso_ct16	9.00E-04	0.016868
s5d	dmso_ct14_vs_dmso_ct20	3.00E-06	0.000104
s5d	dmso_ct14_vs_dmso_ct24	1.00E-08	5.01E-07
s5d	dmso_ct16_vs_dmso_ct24	4.00E-04	0.008435
s5d	chx_xt14_chx_ct24	0.017	0.158453
s5d	dmso_ct14_vs_chx_ct14	5.00E-13	3.85E-11
s5d	dmso_ct16_vs_chx_ct16	7.00E-11	4.45E-09
s5d	dmso_ct20_vs_chx_ct20	2.00E-09	1.09E-07
s5d	dmso_ct24_vs_chx_ct24	2.00E-08	9.64E-07
s5d	anova_time	5.00E-08	2.28E-06
s5d	anova_treatment	9.00E-18	9.60E-16
s5d	anova_time_treatment	0.002	0.032682
s6a	ctrl_ct3_vs_ctrl_ct21	0.013	0.133047
s6a	anova_time	0.01	0.111254
s6b	ctrl_ct9_vs_ctrl_ct21	0.03	0.222367
s6b	mdclk_ct3_vs_mdclk_ct21	1.00E-04	0.002497
s6b	mdclk_ct9_vs_mdclk_ct21	5.00E-05	0.001344
s6b	mdclk_ct15_vs_mdclk_ct21	8.00E-04	0.01527
s6b	anova_time	3.00E-05	0.000849
s6b	anova_time_genotype	0.038	0.252499
s7a	becn1_ctrl	0.009	0.103333
s7a	map1lc3b_ctrl	0.026	0.20505
s7b	ulk1a_ctrl_ct3_vs_ulk1a_ctrl_ct9	0.001	0.018431
s7b	ulk1a_ctrl_ct3_vs_ulk1a_ctrl_ct15	5.00E-06	0.000166
s7b	ulk1a_ctrl_ct3_vs_ulk1a_ctrl_ct21	3.00E-06	0.000104
s7b	ulk1a_ctrl_ct9_vs_ulk1a_ctrl_ct15	0.042	0.265743
s7b	ulk1a_ctrl_ct9_vs_ulk1a_ctrl_ct21	0.029	0.218197
s7b	ulk1a_mclk_ct3_vs_ulk1a_mclk_ct15	0.022	0.185832
s7b	ulk1a_mclk_ct3_vs_ulk1a_mclk_ct21	0.003	0.04523
s7b	ulk1a_ctrl_ct3_vs_ulk1a_mdclk_ct3	0.007	0.086269
s7b	ulk1a_anova_time	6.00E-07	2.34E-05
s7b	ulk1a_anova_time_genotype	0.034	0.238103
s7b	ulk1b_ctrl_ct3_vs_ulk1b_ctrl_ct15	4.00E-05	0.0011
s7b	ulk1b_ctrl_ct9_vs_ulk1b_ctrl_ct15	0.007	0.086269

s7b	ulk1b_ctrl_ct15_vs_ulk1b_ctrl_ct21	0.01	0.111254
s7b	ulk1b_mdclk_ct15_vs_ulk1b_mdclk_ct21	0.01	0.111254
s7b	ulk1b_ctrl_ct15_vs_ulk1b_mdclk_ct15	0.009	0.103333
s7b	ulk1b_ctrl_ct21_vs_ulk1b_mdclk_ct21	0.009	0.103333
s7b	ulk1b_anova_time	5.00E-05	0.001344
s7b	ulk1b_anova_genotype	0.014	0.139747
s7b	ulk1b_anova_time_genotype	0.032	0.230416
s7c_right	ulk1_ct9_ulk1_ct21	0.034	0.238103
s7c_right	ulk1_anova_time	0.021	0.180683
s7d_top_right	ulk1p_ct15_vs_ulk1p_ct9	0.021	0.180683
s7d_top_right	ulk1p_ct21_vs_ulk1p_ct9	0.046	0.277986
s7d_top_right	ulk1p_anova_time	0.012	0.126081
s7d_bottom_ right	ulk1p_ulk1_ratio_ct21_vs_ulk1p_ulk1_ratio_ct9	0.044	0.271982
s7d_bottom_ right	ulk1p_ulk1_ratio_anova_time	0.014	0.139747
s7e	active_dmso_L_vs_active_dmso_D	0.027	0.209543
s7e	inactive_dmso_L_vs_inactive_dmso_D	8.00E-06	0.000255
s7e	active_dmso_L_vs_inactive_dmso_L	8.00E-06	0.000255
s7e	active_btz_mrt_L_vs_inactive_btz_mrt_L	6.00E-08	2.71E-06
s7e	active_dmso_D_vs_inactive_dmso_D	7.00E-11	4.45E-09
s7e	active_dmso_D_vs_active_btz_mrt_D	0.001	0.018431
s7e	active_btz_mrt_D_vs_inactive_btz_mrt_D	2.00E-13	1.59E-11
s7e	inactive_btz_mrt_D_vs_inactive_btz_mrt_D	0.041	0.262532
s8a	without_spiking_dmso_vs_btz	0.024	0.195703
s8a	with_spiking_dmso_vs_btz	2.00E-08	9.64E-07
s8a	anova_treatment	1.00E-09	5.63E-08
s8a	anova_spiking	3.00E-04	0.006572
s8a	anova_treatment_spiking	7.00E-04	0.013634
s8b	p62_dmso_vs_p62_btz	1.00E-05	0.000313
s8b	p62_dmso_vs_p62_baf	0.005	0.067174
s8b	p62_btz_vs_p62_baf	2.00E-04	0.004609
s8b	hsp70_dmso_vs_p62_btz	0.031	0.226439
s8b	hsp70_dmso_vs_p62_baf	0.026	0.20505
s8c	dmso_vs_baf	6.00E-07	2.34E-05
s8c	dmso_vs_baf_btz	6.00E-06	0.000196
s8c	anova_treatment	2.00E-07	8.39E-06
s9a_1st_row	ulk1p_mdclk_ct3_vs_ulk1p_mdclk_ct21	7.00E-04	0.013634
s9a_1st_row	ulk1p_ctrl_ct21_vs_ulk1p_mdclk_ct21	1.00E-04	0.002497
s9a_1st_row	ulk1p_anova_time	0.009	0.103333
s9a_1st_row	ulk1p_anova_genotype	0.016	0.152434
s9a_1st_row	ulk1p_anova_time_genotype	0.017	0.158453
s9a_2nd_row	ulk1_ctrl_ct3_vs_ulk1_ctrl_ct15	0.034	0.238103
s9a_2nd_row	ulk1_ctrl_ct3_vs_ulk1_ctrl_ct21	3.00E-07	1.22E-05
s9a_2nd_row	ulk1_ctrl_ct9_vs_ulk1_ctrl_ct21	6.00E-05	0.001583
s9a_2nd_row	ulk1_ctrl_ct15_vs_ulk1_ctrl_ct21	0.001	0.018431
s9a_2nd_row	ulk1_mdclk_ct3_vs_ulk1_mdclk_ct15	0.03	0.222367
s9a_2nd_row	ulk1_mdclk_ct3_vs_ulk1_mdclk_ct21	2.00E-05	0.000588

s9a_2nd_row	ulk1_mdclk_ct9_vs_ulk1_mdclk_ct21	0.001	0.018431
s9a_2nd_row	ulk1_anova_time	3.00E-09	1.60E-07
s9a_4th_row	eif4ebp_ctrl_ct3_vs_eif4ebp_ctrl_ct9	0.009	0.103333
s9a_4th_row	eif4ebp_ctrl_ct3_vs_eif4ebp_ctrl_ct15	2.00E-05	0.000588
s9a_4th_row	eif4ebp_ctrl_ct3_vs_eif4ebp_ctrl_ct21	4.00E-08	1.85E-06
s9a_4th_row	eif4ebp_ctrl_ct9_vs_eif4ebp_ctrl_ct21	5.00E-04	0.010225
s9a_4th_row	eif4ebp_ctrl_ct15_vs_eif4ebp_mdclk_ct15	0.027	0.209543
s9a_4th_row	eif4ebp_ctrl_ct21_vs_eif4ebp_mdclk_ct21	2.00E-05	0.000588
s9a_4th_row	eif4ebp_anova_time	4.00E-06	0.000135
s9a_4th_row	eif4ebp_anova_genotype	0.043	0.268893
s9a_4th_row	eif4ebp_anova_genotype_time	2.00E-04	0.004609
s9b_left	ctrl_baf_ct3_vs_ctrl_baf_ct15	0.01	0.111254
s9b_left	ctrl_dmso_ct3_vs_ctrl_baf_ct3	0.004	0.056635
s9b_left	ctrl_dmso_ct15_vs_ctrl_btz_ct15	3.00E-05	0.000849
s9b_left	mdclk_dmso_ct15_vs_mdclk_btz_ct15	0.035	0.24182
s9b_left	anova_time	9.00E-05	0.002274
s9b_left	anova_treatment	1.00E-05	0.000313
s9b_right	ctrl_baf	0.034	0.238103
s9b_right	mdclk_baf	0.035	0.24182
s9d_left	clocka_ctrl_vs_clocka_mdclk	2.00E-04	0.004609
s9d_left	per1b_ctrl_vs_per1b_mdclk	0.008	0.095021
s9d_right	clocka_ctrl_vs_clocka_mdclk	0.003	0.04523
s9d_right	per1b_ctrl_vs_per1b_mdclk	0.036	0.245456
s9e_left	ctrl_dmso_vs_ctrl_baf	3.00E-06	0.000104
s9e_left	mdclk_dmso_vs_mdclk_baf	2.00E-05	0.000588
s9e_left	ctrl_dmso_vs_mdclk_dmso	0.039	0.255912
s9e_left	ctrl_baf_vs_mdclk_baf	2.00E-04	0.004609
s9e_left	anova_treatment	0.003	0.04523
s9e_left	anova_genotype	0.001	0.018431
s9e_left	anova_treatment_genotype	0.03	0.222367
s9e_right	ctrl_dmso_vs_ctrl_baf	6.00E-04	0.011955
s9e_right	mdclk_dmso_vs_mdclk_baf	9.00E-04	0.016868
s9e_right	anova_treatment	0.01	0.111254
s9f_left	ctrl_dmso_vs_ctrl_baf	8.00E-06	0.000255
s9f_left	ctrl_dmso_vs_mdclk_dmso	0.004	0.056635
s9f_left	anova_treatment	1.00E-04	0.002497
s9f_left	anova_treatment_genotype	0.002	0.032682
s9f_right	anova_treatment	0.046	0.277986
s10a_left	rps6p_rps6_ratio_dmso_vs_rps6p_rps6_ratio_rapa	0.002	0.032682
s10a_right	eif4ebpp_eif4ebp_ratio_vs_eif4ebpp_eif4ebp_rapa	0.02	0.17538
s10b_left	dmso_vs_rapa	0.012	0.126081
s10b_left	dmso_vs_torin	0.035	0.24182
s10b_left	anova_lay	6.00E-04	0.011955
s10b_left	anova_treatment	0.008	0.095021
s10b_right	dmso_vs_rapa	3.00E-04	0.006572
s10b_right	dmso_vs_torin	0.002	0.032682

s10b_right	anova_treatment	2.00E-04	0.004609
s11g	myf5_dmso_vs_myf5_sr9011	0.018	0.164276
s11g	mrf4_dmso_vs_mrf4_sr9011	0.032	0.230416
s11g	mrf4_dmso_vs_mrf4_sr8278	0.008	0.095021
s12b_left	tsc1a_ctrl_ct9_vs_tsc1a_ctrl_ct21	0.04	0.259255
s12b_left	tsc1a_anova_time	0.037	0.249015
s12b_right	tsc2_ctrl_ct3_vs_tsc2_ctrl_ct21	0.016	0.152434
s12b_right	tsc2_ctrl_ct9_vs_tsc2_ctrl_ct21	4.00E-04	0.008435
s12b_right	tsc2_ctrl_ct21_vs_tsc2_mdclk_ct21	1.00E-05	0.000313
s12b_right	tsc2_ctrl_vs_tsc2_mdclk_median	0.035	0.24182
s12b_right	tsc2_anova_genotype	7.00E-04	0.013634
s12b_right	tsc2_anova_time_genotype	3.00E-04	0.006572
s12c_left	tsc1a_ctrl_ct6_vs_tsc1a_ctrl_ct18	0.006	0.077014
s12c_left	tsc1a_ctrl_ct6_vs_tsc1a_mdclk_ct6	0.038	0.252499
s12c_left	tsc1a_time	0.012	0.126081
s12c_left	tsc1a_time_genotype	0.024	0.195703
s12c_right	tsc2_ctrl_ct6_vs_tsc2_ctrl_ct18	0.044	0.271982
s12d_left	tsc1a_dmso_vs_sr9011	0.017	0.158453
s12d_left	tsc1a_sr9011_vs_sr8278	0.002	0.032682
s12d_left	tsc1b_dmso_vs_sr9011	0.039	0.255912
s12d_left	tsc1b_sr9011_vs_sr8278	0.004	0.056635
s12d_left	tsc2_dmso_vs_sr9011	0.041	0.262532
s12d_left	tsc2_dmso_vs_sr8278	0.018	0.164276
s12d_left	tsc2_sr9011_vs_sr8278	8.00E-04	0.01527
s12d_middle	tsc1b_dmso_vs_nob	0.015	0.146204
s12d_middle	tsc2_dmso_vs_nob	0.008	0.095021
s12d_right	tsc1a_dmso_vs_sr8278_nob	0.019	0.169914
s12d_right	tsc1b_dmso_vs_sr8278_nob	0.001	0.018431
s12d_right	tsc2_dmso_vs_sr8278_nob	0.001	0.018431
s12e	murf1a_dmso_vs_murf1a_sr8278	0.008	0.095021
s12e	murf1a_sr9011_vs_murf1a_sr8278	0.009	0.103333
s12e	murf1a_dmso_vs_murf1a_nob	0.001	0.018431
s12e	murf1a_dmso_vs_murf1a_sr8278_nob	0.001	0.018431
s12e	ulk1a_dmso_vs_ulk1a_sr8278	0.026	0.20505
s12e	ulk1a_dmso_vs_ulk1a_nob	0.011	0.118826
s12e	ulk1a_dmso_vs_ulk1a_sr8278_nob	1.00E-06	3.76E-05

SI References

1. M. Westerfield, The zebrafish book. A guide for the laboratory use of zebrafish (Danio rerio). 4th ed.. (2000).
2. J. J. Kelu, T. G. Pipalia, S. M. Hughes, Circadian regulation of muscle growth independent of locomotor activity. *Proc Natl Acad Sci U S A* **117**, 31208-31218 (2020).
3. J. Berger *et al.*, Loss of Tropomodulin4 in the zebrafish mutant trage causes cytoplasmic rod formation and muscle weakness reminiscent of nemaline myopathy. *Dis Model Mech* **7**, 1407-1415 (2014).
4. S. Higashijima, H. Okamoto, N. Ueno, Y. Hotta, G. Eguchi, High-frequency generation of transgenic zebrafish which reliably express GFP in whole muscles or the whole body by using promoters of zebrafish origin. *Dev Biol* **192**, 289-299 (1997).
5. C. He, C. R. Bartholomew, W. Zhou, D. J. Klionsky, Assaying autophagic activity in transgenic GFP-Lc3 and GFP-Gabarap zebrafish embryos. *Autophagy* **5**, 520-526 (2009).
6. A. Basnakova *et al.*, The habenula clock influences response to a stressor. *Neurobiol Stress* **15**, 100403 (2021).
7. K. Kawakami *et al.*, A transposon-mediated gene trap approach identifies developmentally regulated genes in zebrafish. *Dev Cell* **7**, 133-144 (2004).
8. B. He *et al.*, The Small Molecule Nobiletin Targets the Molecular Oscillator to Enhance Circadian Rhythms and Protect against Metabolic Syndrome. *Cell Metab* **23**, 610-621 (2016).
9. D. J. Kojetin, T. P. Burris, REV-ERB and ROR nuclear receptors as drug targets. *Nat Rev Drug Discov* **13**, 197-216 (2014).
10. V. Adams *et al.*, Small-Molecule Chemical Knockdown of MuRF1 in Melanoma Bearing Mice Attenuates Tumor Cachexia Associated Myopathy. *Cells* **9** (2020).
11. T. S. Bowen *et al.*, Small-molecule inhibition of MuRF1 attenuates skeletal muscle atrophy and dysfunction in cardiac cachexia. *J Cachexia Sarcopenia Muscle* **8**, 939-953 (2017).
12. V. Adams *et al.*, Targeting MuRF1 by small molecules in a HFpEF rat model improves myocardial diastolic function and skeletal muscle contractility. *J Cachexia Sarcopenia Muscle* **13**, 1565-1581 (2022).
13. K. J. Petherick *et al.*, Pharmacological inhibition of ULK1 kinase blocks mammalian target of rapamycin (mTOR)-dependent autophagy. *J Biol Chem* **290**, 11376-11383 (2015).
14. M. Ryzhikov *et al.*, Diurnal Rhythms Spatially and Temporally Organize Autophagy. *Cell Rep* **26**, 1880-1892 e1886 (2019).
15. M. Ryzhikov, A. Eubanks, J. A. Haspel, Measuring Diurnal Rhythms in Autophagic and Proteasomal Flux. *J Vis Exp* 10.3791/60133 (2019).
16. C. H. Emmerich, P. Cohen, Optimising methods for the preservation, capture and identification of ubiquitin chains and ubiquitylated proteins by immunoblotting. *Biochem Biophys Res Commun* **466**, 1-14 (2015).
17. H. Hirata *et al.*, Connexin 39.9 protein is necessary for coordinated activation of slow-twitch muscle and normal behavior in zebrafish. *J Biol Chem* **287**, 1080-1089 (2012).
18. H. Hirata *et al.*, Zebrafish relatively relaxed mutants have a ryanodine receptor defect, show slow swimming and provide a model of multi-minicore disease. *Development* **134**, 2771-2781 (2007).
19. M. Attwaters, J. J. Kelu, T. G. Pipalia, S. M. Hughes, Real Time and Repeated Measurement of Skeletal Muscle Growth in Individual Live Zebrafish Subjected to Altered Electrical Activity. *JoVE* doi:10.3791/64063, e64063 (2022).
20. M. A. Denvir, C. S. Tucker, J. J. Mullins, Systolic and diastolic ventricular function in zebrafish embryos: influence of norepenephrine, MS-222 and temperature. *BMC Biotechnol* **8**, 21 (2008).
21. O. Mirat, J. R. Sternberg, K. E. Severi, C. Wyart, ZebraZoom: an automated program for high-throughput behavioral analysis and categorization. *Front Neural Circuits* **7**, 107 (2013).
22. I. Romero-Calvo *et al.*, Reversible Ponceau staining as a loading control alternative to actin in Western blots. *Anal Biochem* **401**, 318-320 (2010).
23. K. J. Livak, T. D. Schmittgen, Analysis of relative gene expression data using real-time quantitative PCR and the 2(T)(-Delta Delta C) method. *Methods* **25**, 402-408 (2001).

24. M. E. Hughes, J. B. Hogenesch, K. Kornacker, JTK_CYCLE: An Efficient Nonparametric Algorithm for Detecting Rhythmic Components in Genome-Scale Data Sets. *J Biol Rhythms* **25**, 372-380 (2010).
25. G. Wu, R. C. Anafi, M. E. Hughes, K. Kornacker, J. B. Hogenesch, MetaCycle: an integrated R package to evaluate periodicity in large scale data. *Bioinformatics* **32**, 3351-3353 (2016).
26. R. Parsons, R. Parsons, N. Garner, H. Oster, O. Rawashdeh, CircaCompare: a method to estimate and statistically support differences in mesor, amplitude and phase, between circadian rhythms. *Bioinformatics* **36**, 1208-1212 (2020).
27. T. Sellke, M. J. Bayarri, J. O. Berger, Calibration of p-values for testing precise null hypotheses. *American Statistician* **55**, 62-71 (2001).
28. B. Thisse, C. Thisse, Fast Release Clones: A High Throughput Expression Analysis. ZFIN Direct Data Submission (http://zfin.org).
29. R. V. Kondratov, A. A. Kondratova, V. Y. Gorbacheva, O. V. Vykhovanets, M. P. Antoch, Early aging and age-related pathologies in mice deficient in BMAL1, the core componentof the circadian clock. *Genes Dev* **20**, 1868-1873 (2006).
30. K. A. Dyar *et al.*, Muscle insulin sensitivity and glucose metabolism are controlled by the intrinsic muscle clock. *Mol Metab* **3**, 29-41 (2014).
31. K. A. Dyar *et al.*, Transcriptional programming of lipid and amino acid metabolism by the skeletal muscle circadian clock. *PLoS Biol* **16** (2018).
32. E. A. Schroder *et al.*, Intrinsic muscle clock is necessary for musculoskeletal health. *J Physiol* **593**, 5387-5404 (2015).
33. B. D. Harfmann *et al.*, Muscle-specific loss of leads to disrupted tissue glucose metabolism and systemic glucose homeostasis. *Skelet Muscle* **6** (2016).
34. B. A. Hodge *et al.*, The endogenous molecular clock orchestrates the temporal separation of substrate metabolism in skeletal muscle. *Skeletal Muscle* **5** (2015).
35. J. Fernández-Martínez *et al.*, iMS-Bmal1−/− mice show evident signs of sarcopenia that are counteracted by exercise and melatonin therapies. *Journal of Pineal Research* **76**, e12912 (2024).
36. J. G. Smith *et al.*, Liver and muscle circadian clocks cooperate to support glucose tolerance in mice. *Cell Rep* **42** (2023).
37. A. Kumar *et al.*, Brain-muscle communication prevents muscle aging by maintaining daily physiology. *Science* **384**, 563-572 (2024).
38. J. L. Andrews *et al.*, CLOCK and BMAL1 regulate MyoD and are necessary for maintenance of skeletal muscle phenotype and function. *Proc Natl Acad Sci U S A* **107**, 19090-19095 (2010).
39. M. Lowe *et al.*, Cry2 Is Critical for Circadian Regulation of Myogenic Differentiation by Bclaf1-Mediated mRNA Stabilization of Cyclin D1 and Tmem176b. *Cell Reports* **22**, 2118-2132 (2018).
40. S. D. Jordan *et al.*, CRY1/2 Selectively Repress PPARδ and Limit Exercise Capacity. *Cell Metabolism* **26**, 243-+ (2017).
41. N. Katoku-Kikyo *et al.*, Per1/Per2-Igf2 axis-mediated circadian regulation of myogenic differentiation. *Journal of Cell Biology* **220** (2021).
42. P. Pircher, P. Chomez, F. Yu, B. Vennström, L. Larsson, Aberrant expression of myosin isoforms in skeletal muscles from mice lacking the α orphan receptor gene. *American Journal of Physiology-Regulatory Integrative and Comparative Physiology* **288**, R482-R490 (2005).
43. E. Woldt *et al.*, Rev-erb-α modulates skeletal muscle oxidative capacity by regulating mitochondrial biogenesis and autophagy. *Nat Med* **19**, 1039-+ (2013).
44. A. Mayeuf-Louchart *et al.*, Rev-erb-α regulates atrophy-related genes to control skeletal muscle mass. *Sci Rep* **7** (2017).
45. S. Chatterjee *et al.*, The Nuclear Receptor and Clock Repressor Rev-erbalpha Suppresses Myogenesis. *Sci Rep* **9**, 4585 (2019).
46. A. Boulinguiez *et al.*, NR1D1 controls skeletal muscle calcium homeostasis through myoregulin repression. *JCI Insight* **7** (2022).
47. R. D. Welch, C. Billon, A. Kameric, T. P. Burris, C. A. Flaveny, Rev-erbalpha heterozygosity produces a dose-dependent phenotypic advantage in mice. *PLoS One* **15**, e0227720 (2020).
48. R. D. Welch *et al.*, Rev-Erb co-regulates muscle regeneration via tethered interaction with the NF-Y cistrome. *Molecular Metabolism* **6**, 703-714 (2017).

49. S. Raichur *et al.*, Identification and validation of the pathways and functions regulated by the orphan nuclear receptor, ROR alpha1, in skeletal muscle. *Nucleic Acids Research* **38**, 4296-4312 (2010).
50. M. Y. Wang, Z. M. Zhong, Y. B. Zhong, W. Zhang, H. Wang, The Zebrafish Period2 Protein Positively Regulates the Circadian Clock through Mediation of Retinoic Acid Receptor (RAR)-related Orphan Receptor alpha (Ror alpha). *J Biol Chem* **290**, 4367-4382 (2015).
51. T. E. Sztal, E. A. McKaige, C. Williams, A. A. Ruparelia, R. J. Bryson-Richardson, Genetic compensation triggered by actin mutation prevents the muscle damage caused by loss of actin protein. *PLoS Genet* **14** (2018).
52. S. A. Mawed, J. Zhang, F. Ren, Y. He, J. Mei, atg7 and beclin1 are essential for energy metabolism and survival during the larval-to-juvenile transition stage of zebrafish. *Aquac Fish* **7**, 359-372 (2022).
53. G. Huang, F. Zhang, Q. Ye, H. Wang, The circadian clock regulates autophagy directly through the nuclear hormone receptor Nr1d1/Rev-erbalpha and indirectly via Cebpb/(C/ebpbeta) in zebrafish. *Autophagy* **12**, 1292-1309 (2016).
54. N. Hamilton, N. Diaz-de-Cerio, D. Whitmore, Impaired light detection of the circadian clock in a zebrafish melanoma model. *Cell Cycle* **14**, 1232-1241 (2015).
55. M. Ganassi, S. Badodi, K. Wanders, P. S. Zammit, S. M. Hughes, Myogenin is an essential regulator of adult myofibre growth and muscle stem cell homeostasis. *Elife* **9** (2020).
56. H. F. Wang *et al.*, Single-cell in vivo imaging of cellular circadian oscillators in zebrafish. *PLoS Biol* **18** (2020).
57. O. Yogev, V. C. Williams, Y. Hinits, S. M. Hughes, eIF4EBP3L Acts as a Gatekeeper of TORC1 In Activity-Dependent Muscle Growth by Specifically Regulating Mef2ca Translational Initiation. *PLoS Biol* **11** (2013).
58. R. M. Ceinos *et al.*, Mutations in blind cavefish target the light-regulated circadian clock gene,. *Sci Rep* **8** (2018).
59. V. T. Nguyen *et al.*, Conservation of the Nrf2-Mediated Gene Regulation of Proteasome Subunits and Glucose Metabolism in Zebrafish. *Oxid Med Cell Longev* **2016** (2016).
60. J. Berger, P. D. Currie, Zebrafish models flex their muscles to shed light on muscular dystrophies. *Dis Model Mech* **5**, 726-732 (2012).
61. M. W. Davis, E. M. Jorgensen, ApE, A Plasmid Editor: A Freely Available DNA Manipulation and Visualization Program. *Front Bioinform* **2** (2022).
